# Supplementary material for: The Pseudomonas aeruginosa T6SS Delivers a Periplasmic Toxin that Disrupts Bacterial Cell Morphology
Source: Cell Rep. 2019 Oct 1;29(1):187–201.e7. doi: 10.1016/j.celrep.2019.08.094 (PMC6899460; doi:10.1016/j.celrep.2019.08.094)
Supplement: Document S1. Figures S1–S7 and Tables S1–S3 [file mmc1.pdf]

Cell Reports, Volume *29*

Supplemental Information

The *Pseudomonas aeruginosa* T6SS Delivers a Periplasmic Toxin that Disrupts Bacterial Cell Morphology

Thomas E. Wood, Sophie A. Howard, Andreas Förster, Laura M. Nolan, Eleni Manoli, Nathan P. Bullen, Hamish C.L. Yau, Abderrahman Hachani, Richard D. Hayward, John C. Whitney, Waldemar Vollmer, Paul S. Freemont, and Alain Filloux

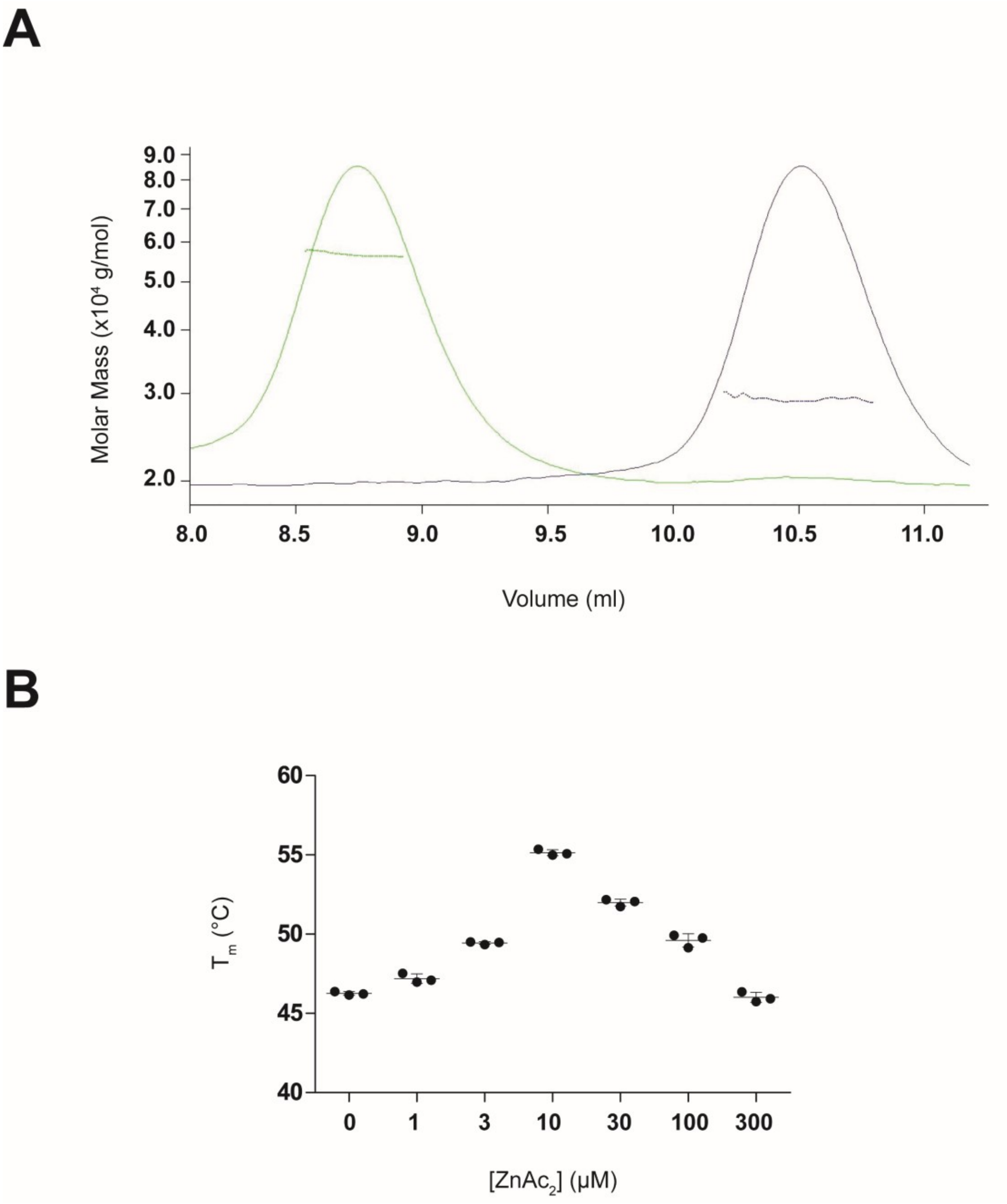

Figure S1: The VgrG2b metallopeptidase exists as monomeric and dimeric species in solution and is stabilised by zinc. Related to Figure 2.

A SEC-MALLS chromatogram of VgrG2b$_{C\text{-}ter}$ purified from *E. coli*. Solid lines show UV traces of fractions pooled from two peaks obtained after preparatory size-exclusion chromatography. Dashed lines across the peaks show the MALLS-derived apparent masses. The calculated weight-average molecular masses are 56.7 kD (left peak) and 29.1 kD (right peak) corresponding to the dimeric and monomeric species, respectively.

B Thermostability of the purified VgrG2b$_{C\text{-}ter}$ domain in the presence of different concentrations of zinc ions as determined by calculation of the melting temperature using differential scanning fluorimetry. Individual points represent the mean of three technical replicates with error bars showing the SEM (n = 3 technical replicates).

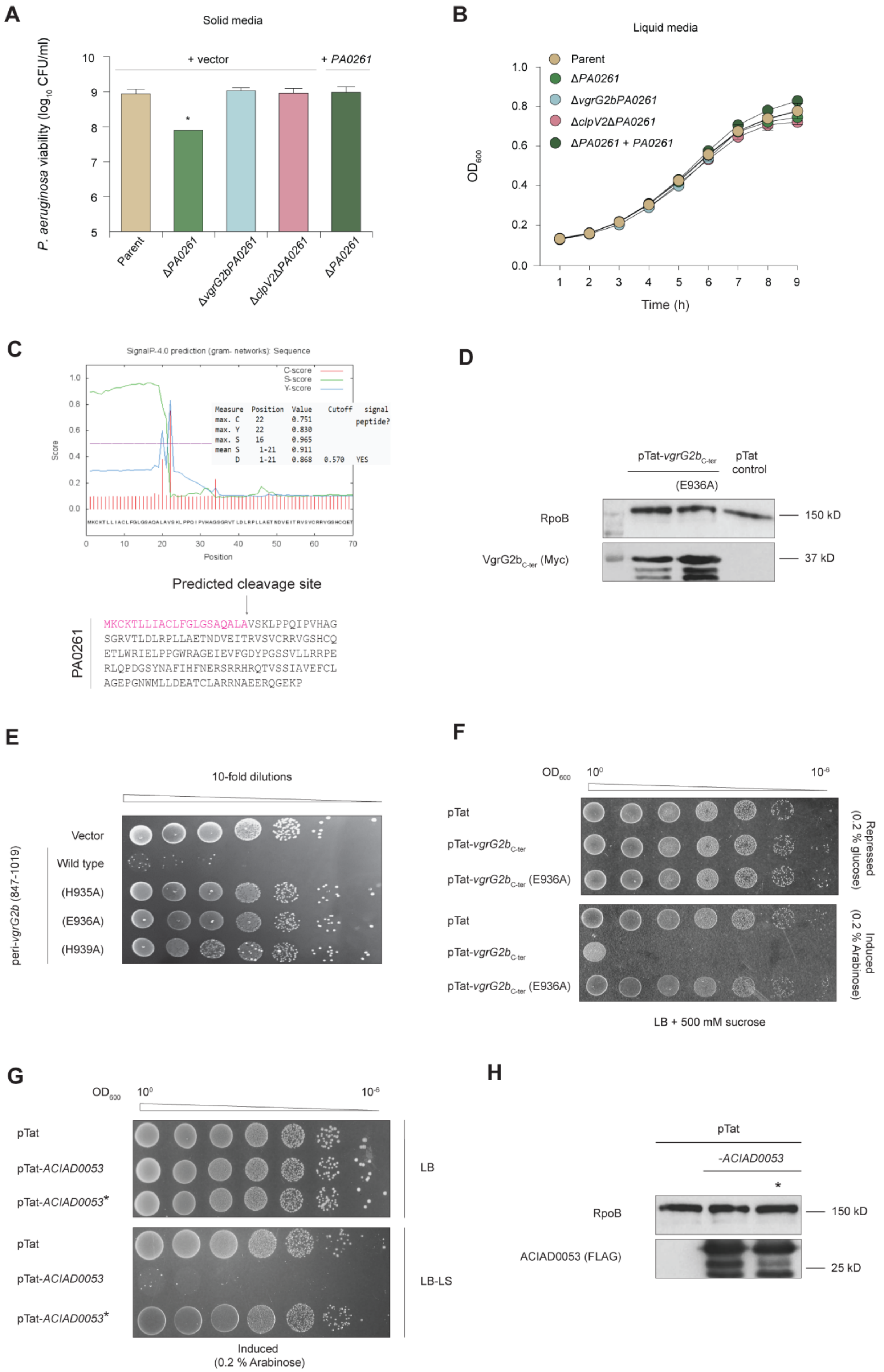
A
Solid media
P. aeruginosa viability (log_{10} CFU/ml)
+ vector
+ PA0261
10
9
8
7
6
5
*
Parent
ΔPA0261
ΔvgrG2bPA0261
ΔclpV2ΔPA0261
ΔPA0261
B
Liquid media
Parent
ΔPA0261
ΔvgrG2bPA0261
ΔclpV2ΔPA0261
ΔPA0261 + PA0261
OD_{600}
1.0
0.8
0.6
0.4
0.2
0.0
1
2
3
4
5
6
7
8
9
Time (h)
C
SignalP-4.0 prediction (gram- networks): Sequence
C-score
S-score
Y-score
Score
Position
Measure Position Value Cutoff signal peptide?
max. C 22 0.751
max. Y 22 0.830
max. S 16 0.965
mean S 1-21 0.911
D 1-21 0.868 0.570 YES
Predicted cleavage site
PA0261
MKCKTLLIACLFGLGSAQALAVSKLPPQIPVHAG
SGRVTLDLRPLLAETNDVEITRVSVCRRVGSHCQ
ETLWRIELPPGWRAGEIEVFGDYPGSSVLLRRPE
RLQPDGSYNAFIHFNERSRRHRQTVSSIAVEFCL
AGEPGNWMLLDEATCLARRNAEERQGEKP
D
pTat-vgrG2b$_{C-ter}$
(E936A)
pTat control
RpoB
VgrG2b$_{C-ter}$ (Myc)
150 kD
37 kD
E
10-fold dilutions
peri-vgrG2b (847-1019)
Vector
Wild type
(H935A)
(E936A)
(H939A)
F
OD_{600}
10^0
10^{-6}
pTat
pTat-vgrG2b$_{C-ter}$
pTat-vgrG2b$_{C-ter}$ (E936A)
Repressed (0.2 % glucose)
Induced (0.2 % Arabinose)
LB + 500 mM sucrose
G
OD_{600}
10^0
10^{-6}
pTat
pTat-ACIAD0053
pTat-ACIAD0053*
LB
LB-LS
Induced (0.2 % Arabinose)
H
pTat
-ACIAD0053
*
RpoB
ACIAD0053 (FLAG)
150 kD
25 kD

Figure S2: Characterisation of the VgrG2b-PA0261 effector-immunity pair family. Related to Figure 4.
A Self-intoxication assay of *P. aeruginosa* on solid media. Viability of the indicated strains assessed by enumeration of colony forming units (CFU). Here, the parental strain is *P. aeruginosa* PA14Δ*rsmA*Δ*amrZ*. A statistically significant difference in recovered prey *versus* the parental strain was determined using a one-way ANOVA followed by Dunnett's multiple comparisons test (n = 3 biological replicates; * $p < 0.05$).
B Self-intoxication assay of *P. aeruginosa* in liquid culture. Viability was measured by monitoring turbidity (OD_{600}) in a 96-well format. The parental strain is the same as in panel **A**. Mean values are plotted from three biologically independent experiments, with error bars representing the standard deviation (n = 3).
C The putative N-terminal signal peptide of PA0261. The upper panel shows the prediction output of the SignalP 4.1 server. The lower panel indicates the predicted signal peptide (in pink) and putative signal peptidase cleavage site within the sequence of the PA0261 polypeptide.
D Immunoblot showing that both active and inactive $VgrG2b_{C\text{-}ter}$ variants are produced in *E. coli* from the pTat plasmids. Both isoforms are detected with anti-myc antibodies to a C-terminal tag. RpoB is a loading control.
E *E. coli* periplasmic toxicity assay showing the requirement of the metallopeptidase catalytic triad. VgrG2b metallopeptidase constructs (residues 847-1019) were targeted to the periplasm by an N-terminal PelB signal peptide. Strains were serially diluted on solid media under inducing conditions. Image is representative of three independent experiments.
F Plating efficiency of *E. coli* harbouring pTat, pTat-$vgrG2b_{C\text{-}ter}$ or pTat-$vgrG2b_{C\text{-}ter}$ (E936A) in hypertonic conditions (LB agar with 500 mM sucrose). Expression from the plasmid is repressed with 0.2% glucose or induced with 0.2% arabinose.
G Targeting the *A. baylyi* $VgrG2b_{C\text{-}ter}$ metallopeptidase homologue ACIAD0053 to the *E. coli* periplasm using the TorA Tat-dependent signal peptide of the pTat vector. ACIAD0053* is the catalytic mutant of the effector where the HEMGH motif (residues 102-106) has been mutated to AAMGA. Plating efficiency of ten-fold serially diluted *E. coli* cultures on LB agar or low-salt LB agar (LB-LS) containing 0.2% arabinose inducer are shown. Image is representative of three independent experiments.
H Immunoblot detecting the FLAG-tagged ACIAD0053 toxin and its inactive AAMGA variant (designated by the asterisk). RpoB is a loading control.

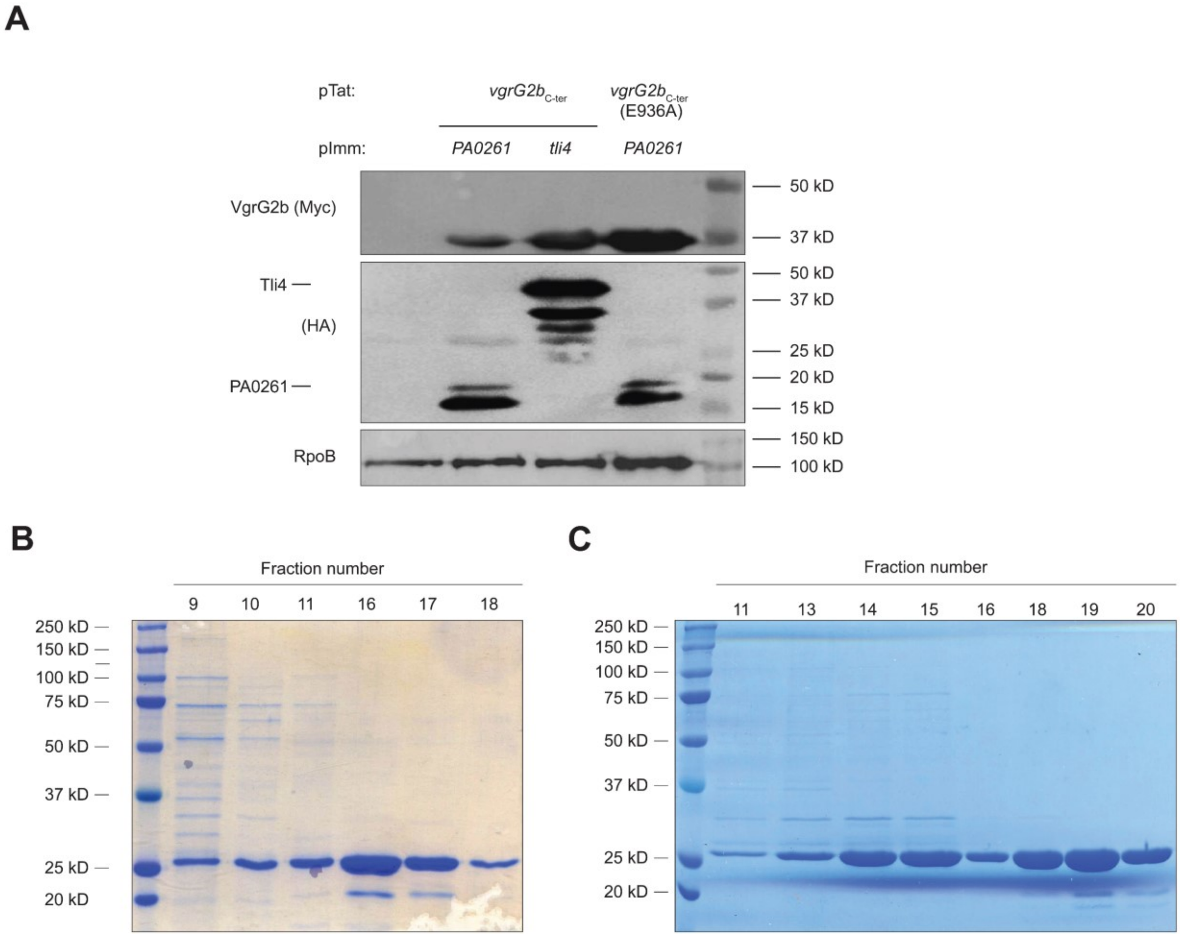

Figure S3: Production of immunity proteins and purification of VgrG2b$_{C\text{-}ter}$. Related to Figure 5.

A Immunoblot showing the production of the Myc-tagged VgrG2b$_{C\text{-}ter}$ effector and HA-tagged immunity proteins expressed in *E. coli*. RpoB acts as a loading control.

B and **C** SDS-PAGE analysis of recombinant VgrG2b$_{C\text{-}ter}$ (**B**) and VgrG2b$_{C\text{-}ter}$ (E936A) (**C**) elution after size-exclusion chromatography following Ni^{2+}-NTA affinity chromatography. Proteins were visualised by Coomassie staining. Fraction numbers are shown for each lane.

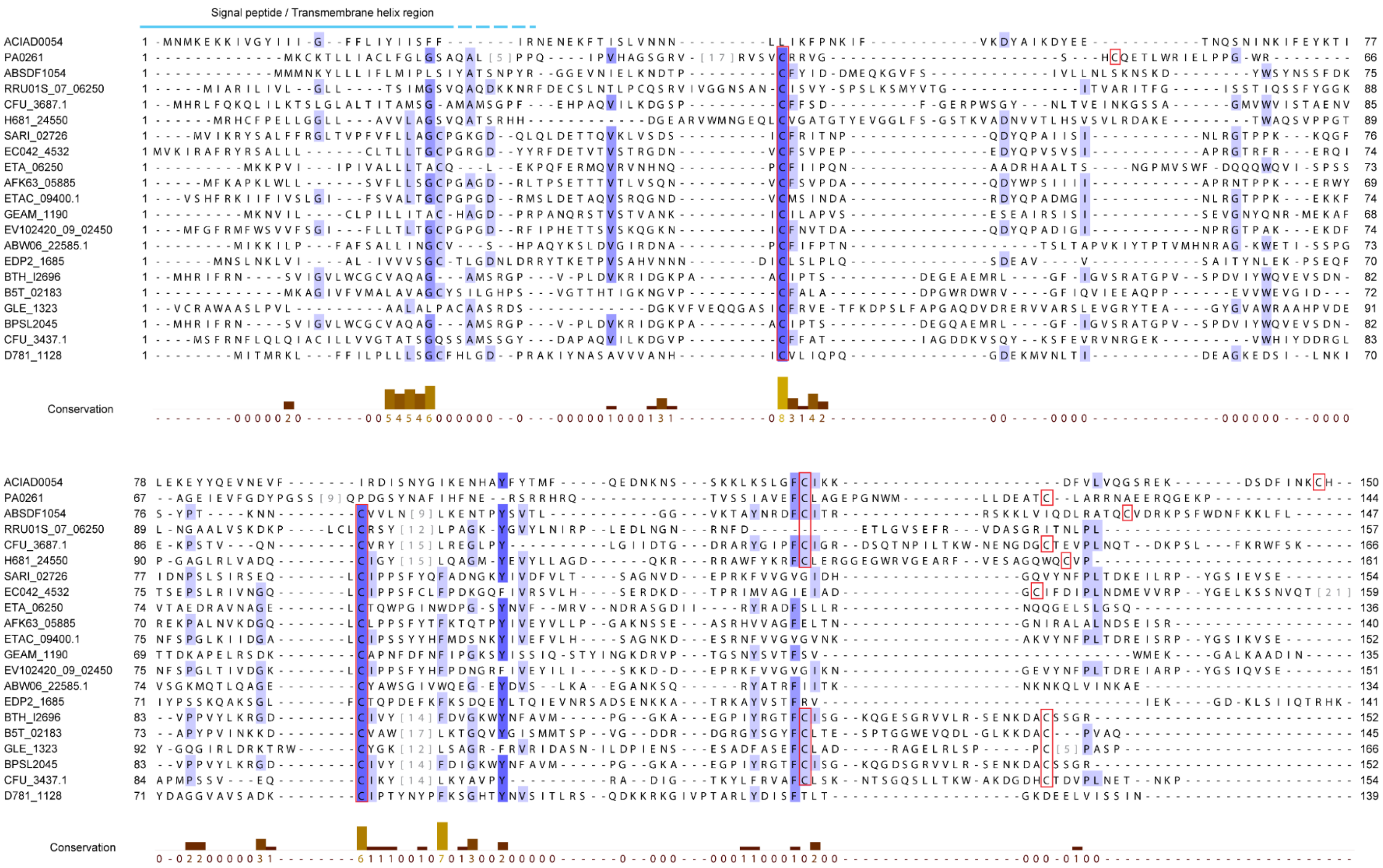

Figure S4: Alignment of immunity proteins in the PA0261 family. Related to Figure 5.

Multiple sequence alignment of putative immunity proteins for metallopeptidase effectors of the VgrG2b$_{C\text{-}ter}$ family. Due to low sequence homology, the alignment was manually curated after initial alignment by MAFFT. Omitted residues are shown in grey and cysteine residues are highlighted with red boxes. Darker coloration of the residues reflects the increasing conservation of identity, with the scores for each position shown below the alignment.

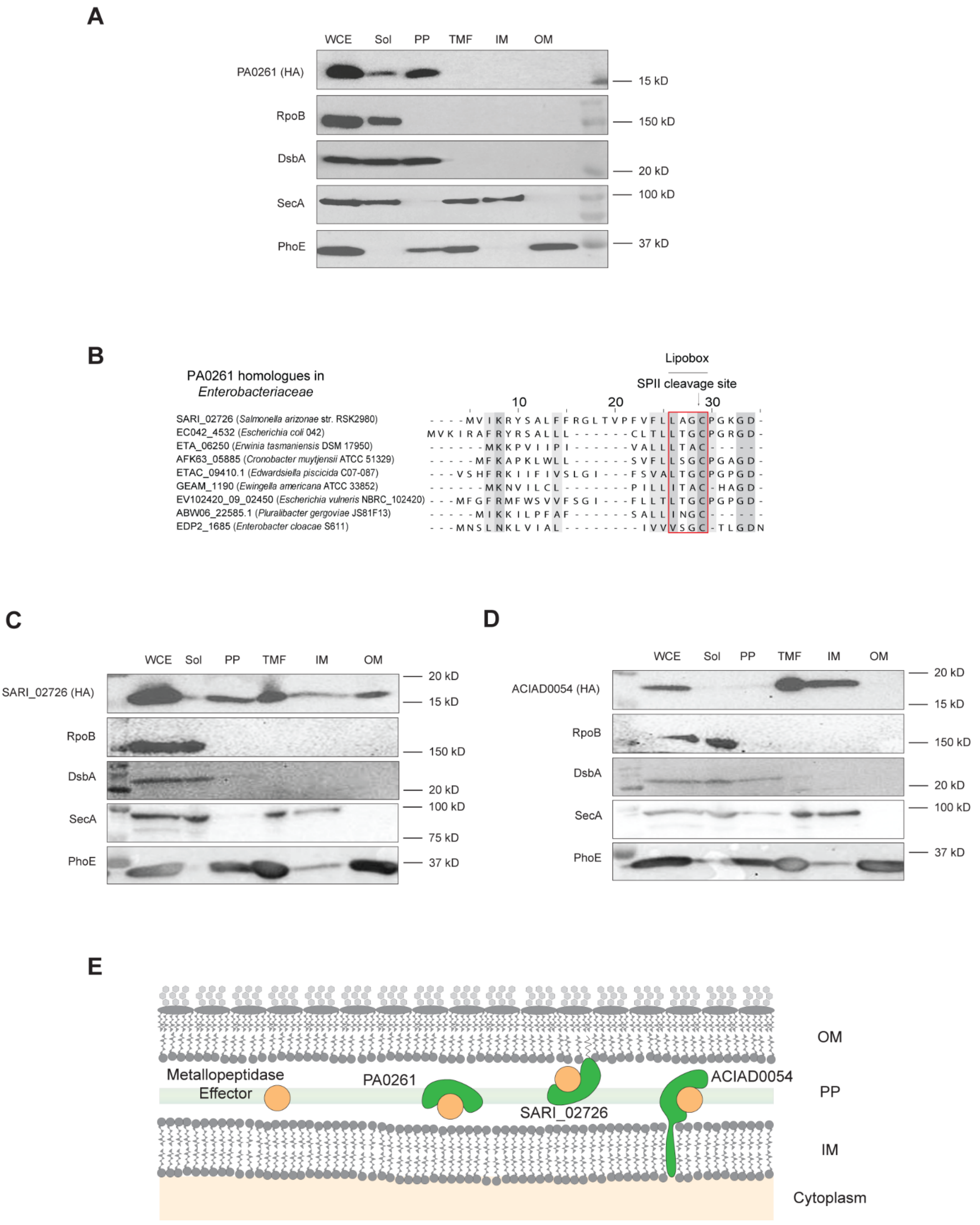
A
WCE
Sol
PP
TMF
IM
OM
PA0261 (HA)
RpoB
DsbA
SecA
PhoE
15 kD
150 kD
20 kD
100 kD
37 kD
B
PA0261 homologues in
Enterobacteriaceae
Lipobox
SPII cleavage site
10
20
30
SARI_02726 (Salmonella arizonae str. RSK2980)
EC042_4532 (Escherichia coli 042)
ETA_06250 (Erwinia tasmaniensis DSM 17950)
AFK63_05885 (Cronobacter muytjensii ATCC 51329)
ETAC_09410.1 (Edwardsiella piscicida C07-087)
GEAM_1190 (Ewingella americana ATCC 33852)
EV102420_09_02450 (Escherichia vulneris NBRC_102420)
ABW06_22585.1 (Pluralibacter gergoviae JS81F13)
EDP2_1685 (Enterobacter cloacae S611)
C
SARI_02726 (HA)
75 kD
D
ACIAD0054 (HA)
E
OM
PP
IM
Cytoplasm
Metallopeptidase
Effector
PA0261
SARI_02726
ACIAD0054

Figure S5: Members of the PA0261 immunity protein family utilise diverse mechanisms to access the periplasmic compartment. Related to Figure 5.

A Immunoblot analysis of the subcellular localisation of HA-tagged PA0261. RpoB is used as a marker of the soluble fraction (Sol), DsbA for the periplasm (PP), SecA for the inner membrane (IM) and PhoE for the outer membrane (OM). WCE: whole cell extract; TMF: total membrane fraction. Immunoblots are representative of three independent experiments.

B Multiple sequence alignment of the N-terminal region of PA0261 orthologues within *Enterobacteriaceae*. The red box demarcates a putative lipobox sequence and the predicted signal peptidase II (SPII) cleavage site is marked. Light to dark grey shading indicates an increasing level of residue conservation.

C and **D** Immunoblot of fractionated *E. coli* cells producing HA-tagged PA0261 orthologues from *S. arizonae* (SARI_02726; **C**) and *A. baylyi* (ACIAD0054; **D**). See panel **A** for details of fraction markers.

E Model showing the subcellular localisation of the three metallopeptidase immunity proteins PA0261, SARI_02726 and ACIAD0054. These immunity determinants, depicted in green, are a soluble periplasmic protein, an outer membrane lipoprotein and an inner membrane protein, respectively. The periplasmic VgrG2b$_{C\text{-}ter}$-family metallopeptidase is represented as an orange circle. Immunoblots are representative of three independent experiments.

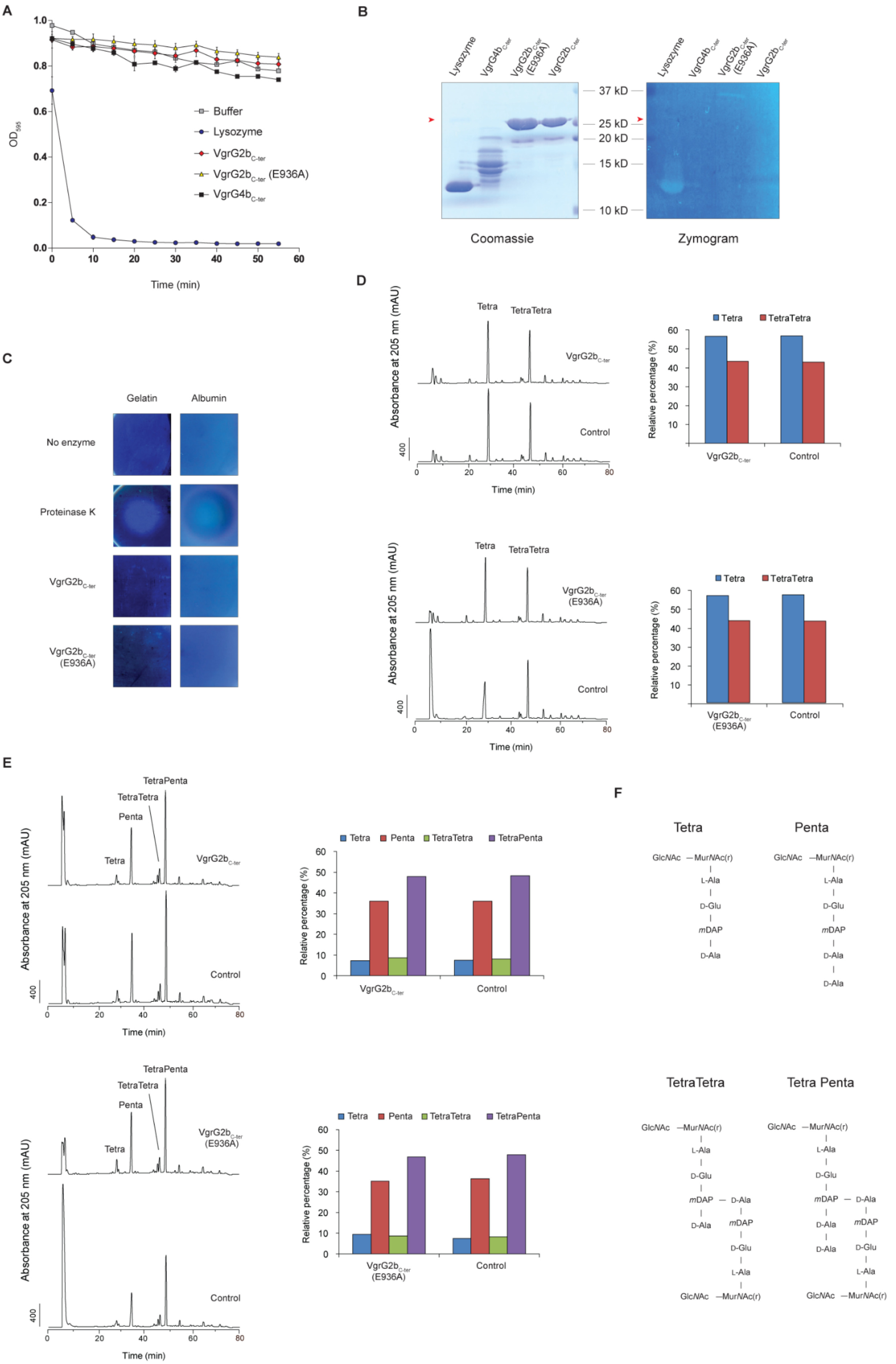

A
OD595
Time (min)
Buffer
Lysozyme
VgrG2bC-ter
VgrG2bC-ter (E936A)
VgrG4bC-ter
B
Lysozyme
VgrG4bC-ter
VgrG2bC-ter (E936A)
VgrG2bC-ter
37 kD
25 kD
20 kD
15 kD
10 kD
Coomassie
Zymogram
C
Gelatin
Albumin
No enzyme
Proteinase K
VgrG2bC-ter
VgrG2bC-ter (E936A)
D
Absorbance at 205 nm (mAU)
Tetra
TetraTetra
Control
Time (min)
Relative percentage (%)
E
TetraPenta
Penta
F
GlcNAc
MurNAc(r)
L-Ala
D-Glu
mDAP
D-Ala
Tetra Penta

Figure S6: VgrG2b$_{C-ter}$ displays no detectable peptidoglycan hydrolase or generic protease activity. Related to Figure 6.

A Recombinant VgrG2b$_{C-ter}$ does not cause lysis of *M. lysodeikticus* as measured by turbidometry. Incubation with lysozyme acts as the positive control, while the buffer-only condition and addition of recombinant VgrG4b$_{C-ter}$ act as the negative controls. Points and error bars represent the mean ± SEM (*n* = 3 technical replicates).

B Recombinant VgrG2b$_{C-ter}$, VgrG2b$_{C-ter}$ (E936A), VgrG4b$_{C-ter}$ and lysozyme were assessed for peptidoglycan hydrolytic activity by zymography with a purified *E. coli* peptidoglycan substrate. Samples were run on a 12% SDS-PAGE gel containing 0.1% peptidoglycan and stained with a methylene blue solution to visualise zones of clearing, indicative of peptidoglycan hydrolysis. A Coomassie-stained gel acts as a loading control for the purified proteins. The arrows highlight the location of the band corresponding to VgrG2b$_{C-ter}$.

C 10 µg VgrG2b$_{C-ter}$, VgrG2b$_{C-ter}$ (E936A) or proteinase K was spotted onto bacteriological agar plates containing 1% bovine serum albumin or gelatin. After incubation at 37 °C for 24 h, amido black staining was used to visualise zones of clearing indicative of proteolytic activity.

D and **E** HPLC chromatograms showing the profiles of the major muropeptides (*left panels*) released from tetrapeptide-rich *E. coli* MC1061 (**D**) and pentapeptide-rich CS703/1 (**E**) sacculi after incubation with VgrG2b$_{C-ter}$, VgrG2b$_{C-ter}$ (E936A) or a buffer-only control. The composition of muropeptide species in control and assay reactions is also shown (*right panels*).

F Structures of the muropeptides shown in panels **D** and **E**. Abbreviations: Glc*N*Ac, *N*-acetylglucosamine; Mur*N*Ac(r), *N*-acetylmuramitol; L-Ala, L-alanine; D-Glu, D-glutamic acid; *m*DAP, *meso*-diaminopimelic acid; D-Ala, D-alanine.

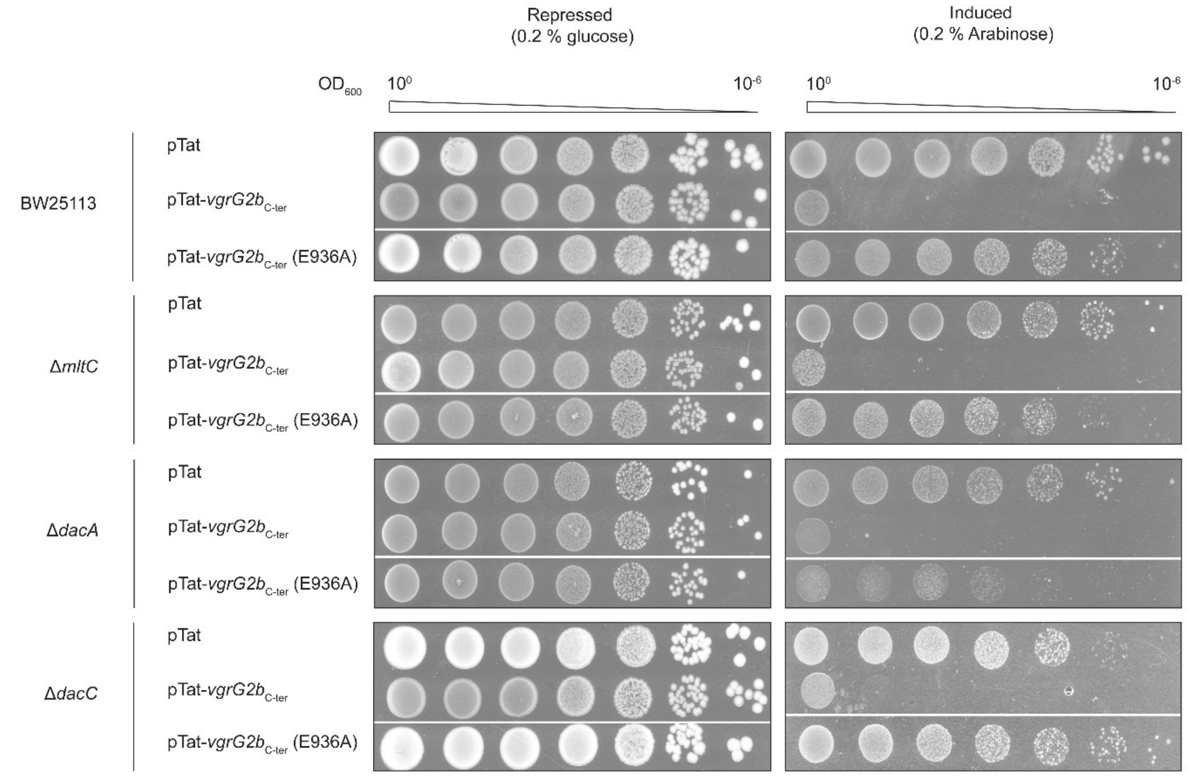

Figure S7: MltC, PBP5 and PBP6a individually dispensable for VgrG2b$_{\text{C-ter}}$-mediated toxicity. Related to Figure 7.

Plating efficiency of *E. coli* strains harbouring pTat vector, pTat-*vgrG2b*$_{\text{C-ter}}$ or pTat-*vgrG2b*$_{\text{C-ter}}$ (E936A). Strains are wild type BW25113, *mltC*, *dacA* (encoding PBP5) and *dacC* (encoding PBP6a) mutants. Serial dilutions assessed toxicity in repressive (0.2% glucose) and inducing (0.2% arabinose) conditions. Images are representative of three biological replicates.

Table S1

	VgrG2b (833-1013)
Data processing	
Wavelength (Å)	0.9795
Resolution Range (Å)	43.9 - 3.2 (3.42 - 3.2)[a]
Space Group	$P3_1$
Unit Cell	
a, *b*, *c* (Å)	78.3, 78.3, 115.3
α, *β*, *γ* (°)	90, 90, 120
Total Reflections	45339
Unique Reflections	12782
Multiplicity	3.5 (3.6)[a]
Completeness (%)	99.6 (99.9)[a]
$I/\sigma I$	12.5 (2.0)[a]
d_{min} for I/sig(I) > 1.0 (Å)	3.1
Wilson B-factor	123.5
R_{sym} (%)	5.7 (46.2)[a]
Model refinement	
R value	0.223
R_{free}	0.232
Number of Atoms	2745
Protein Residues	360
Water Molecules	0
Root-mean-square Deviations	
Bond lengths (Å)	0.009
Bond angles (°)	1.01
Ramachandran Favoured (%)	95.5%
Ramachandran Outliers (%)	0
Clash Score	4

a. Values within parentheses denote the resolution of the highest resolution shell

Table S1: Data collection and refinement statistics for the crystal structure of the VgrG2b metallopeptidase domain. Related to Figure 2.

Table S2

	Pos 1	Pos 4	Pos 5	Pos 6	All data
**Data collection**					
Wavelength (Å)	0.97949				
Oscillation width (°)	0.15				
Number of images	2400				9600
**Data processing**					
Space group	P 4 2 2				
Unit cell parameters	168.8, 79.37	169.4, 79.37	169.4, 79.20	168.7, 79.15	168.8, 79.37
Resolution range (Å)	40-2.7 (2.86-2.7)[a]	40-2.8 (2.97-2.8)[a]	40-2.9 (3.07-2.9)[a]	40-2.8 (2.97-2.8)[a]	40-2.7 (2.85-2.7)[a]
Unique reflections	60251 (9706)[a]	54432 (8760)[a]	48913 (7849)[a]	53864 (8712)[a]	60184 (8967)[a]
Multiplicity	14.0 (13.5)[a]	13.6 (12.1)[a]	13.7 (13.6)[a]	13.5 (12.2)[a]	49.3 (20.6)[a]
R_{meas} (lowest resolution)	0.073	0.061	0.071	0.079	0.095
ISa	14.7	16.7	21.8	14.2	N/A
R_{meas}	0.323 (6.17)[a]	0.304 (9.26)[a]	0.413 (8.15)[a]	0.345 (6.53)[a]	0.381 (7.60)[a]
Completeness	0.999 (0.998)[a]	0.999 (0.999)[a]	0.999 (0.997)[a]	1.000 (1.000)[a]	0.999 (0.999)[a]
$\langle I/\sigma(I)\rangle$	6.66 (0.13)[a]	7.80 (0.16)[a]	7.38 (0.22)[a]	6.65 (0.23)[a]	9.91 (0.13)[a]
$CC_{1/2}$	0.997 (0.277)[a]	0.997 (0.124)[a]	0.996 (0.201)[a]	0.997 (0.256)[a]	0.999 (0.251)[a]
d_{min} for I/sig(I) > 1.0 (Å)	3.0	3.1	3.2	3.1	3.0
d_{min} for SigAno[b] > 1.0 (Å)	4.65	4.82	3.88	4.19	4.27

a. Values within parentheses denote the resolution of the highest resolution shell
b. SigAno = $|F^{+}-F^{-}|/\sigma(F)$

Table S2: Data collection and processing statistics for crystals of selenomethionine-derivatised VgrG2b metallopeptidase domain. Related to Figure 2.

Table S3

UniProt Accession	Protein[a]	Replicate 1				Replicate 2					Replicate 3				
		Coverage (%)	Unique Peptides	MASCOT Score (a)	MASCOT Score (b)	Coverage (%)	Unique Peptides	MASCOT Score (a)	MASCOT Score (b)	MASCOT Score (c)	Coverage (%)	Unique Peptides	MASCOT Score (a)	MASCOT Score (b)	MASCOT Score (c)
Cell envelope															
Q9I6M7	VgrG2b$_{C-ter}$ (E936A)	88	26		14949	95	29	2014	24367	4989	92	26	298	8024	192
P0C066	MltC[d]	62	12		489						35	6		103	
P76537	YfeY[d]	37	4		155	36	4		159		29	4	0	379	26
P08506	PBP6a					58	14	46	455		44	15	30	172	
P0AEB4	PBP5					58	18	215	417		29	9	23	156	23
P18392	RstB					13	4		237		17	5		203	
P0A938	BamE[d]	76	3		199						76	3	0	166	
P69778	Lpp[d]	33	2	28	131	33	2	53	129		33	2	16	283	20
A7ZKY3	LolB[d]	54	9		227						46	8		113	
P0AC04	BamD[d]					33	6	119	250		39	7		152	
P77774	BamB[d]					47	10	77	189		35	9	14	157	
P0A903	BamC[d]	22	5	14	153						46	9	45	129	
P0AAS1	YlaC					33	4	31	195		33	5		134	
Cytoplasmic															
P0ACZ8	CusR	52	11	326	824	45	7	72	251	211					
P13035	GlpD					69	28	56	1063		33	13	44	169	28
P26616	MaeA	32	9	20	287	52	19	287	695						
P67095	YfcE					36	4	39	154	35	28	3	19	100	25
P0AGK8	IscR	38	3	35	108	54	4	34	220	0					
P0AEZ1	MetF	23	5	58	117	20	4	40	133	61					
P00561	ThrA					30	13	49	224		13	8	34	136	40
Cell envelope (below threshold)															
P69412	RcsF[d]	26	3		158						10	1		33	

a: BL21 pET22b-*vgrG2b*$_{C-ter}$ (E936A)
b: BL21 pET22b-*vgrG2b*$_{C-ter}$ (E936A)-*strepII*
c: BL21 pET22b-*vgrG2a*$_{C-ter}$ -*strepII*
d: Lipoprotein

Table S3: Interactome of VgrG2b$_{C\text{-}ter}$ (E936A) in the *E. coli* cell envelope. Related to Figure 7.

The MASCOT scores indicate the enrichment of the proteins pulled down within the three independent replicates, presenting the interactome of periplasmic VgrG2b$_{C\text{-}ter}$ (E936A) in *E. coli*. Proteins localised in the cytoplasm were discounted as relevant interaction partners. The UniProt accession number, defining identity of interacting partners, is also shown alongside the coverage and the number of unique peptides found in each replicate. The MASCOT score corresponds to -10log(*P*), where *P* is the calculated probability that the observed match between the experimental data and the database sequence is a random event and serves as a statistical representation of the positive identification of the protein in the sample, with higher scores being more significant. Note that replicates were entirely independent so quantitative comparisons may not be conducted.
