# Supplementary figures and images for: The Pseudomonas aeruginosa T6SS Delivers a Periplasmic Toxin that Disrupts Bacterial Cell Morphology
Source: Cell Rep. 2019 Oct 1;29(1):187–201.e7. doi: 10.1016/j.celrep.2019.08.094 (PMC6899460; doi:10.1016/j.celrep.2019.08.094)

Figure 1D

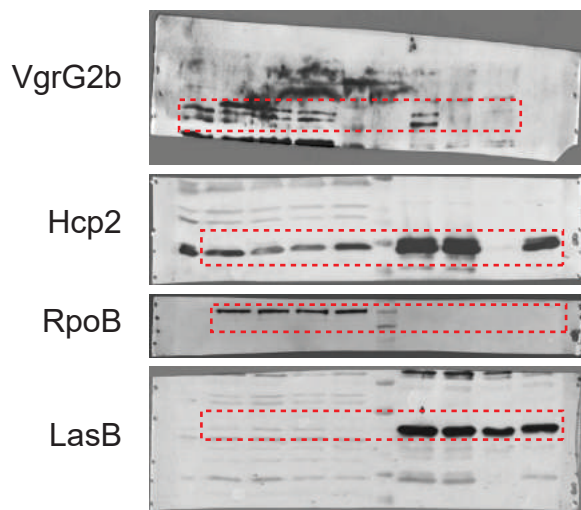

Figure 5B

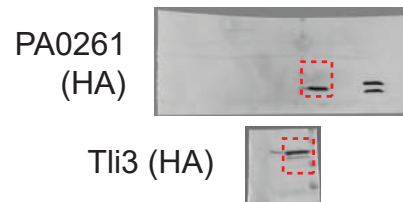

Figure 5D

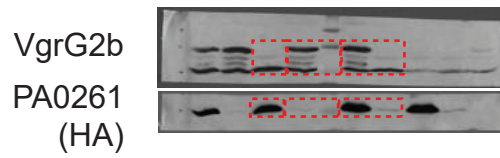

Figure 7

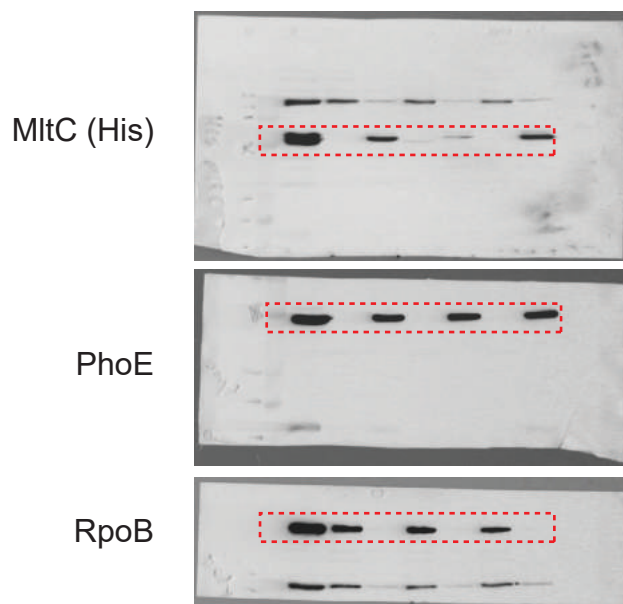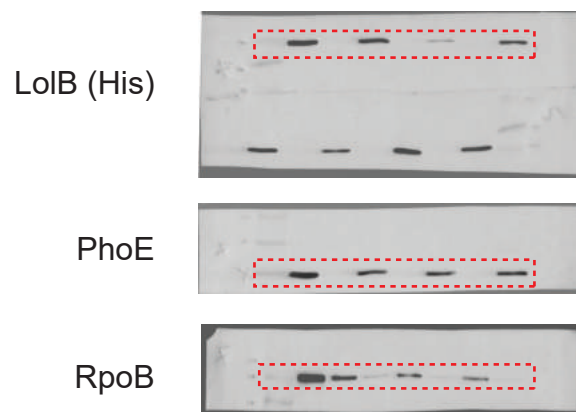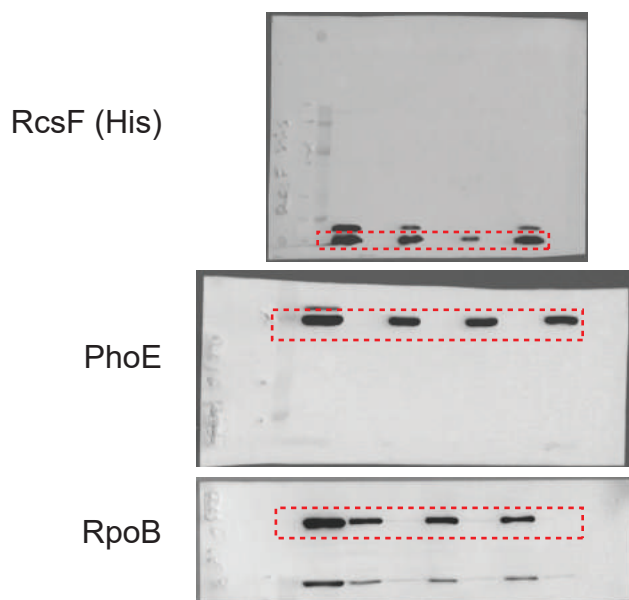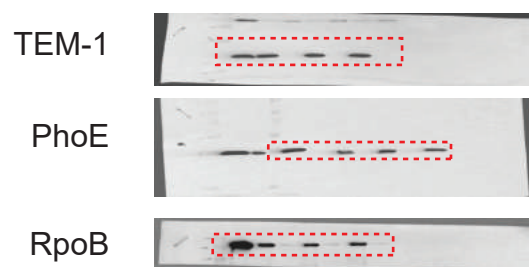

Figure S2

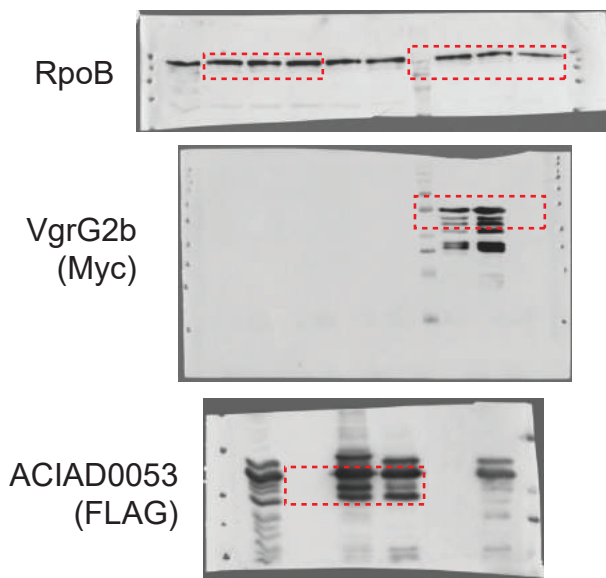

Figure S3

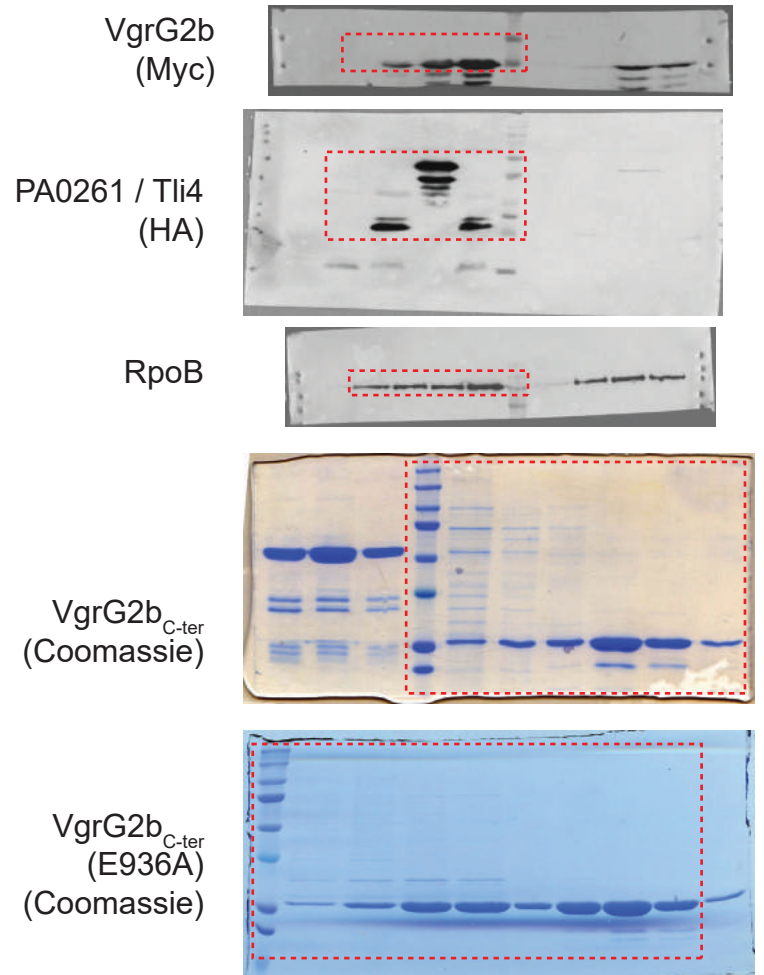

Figure S6B

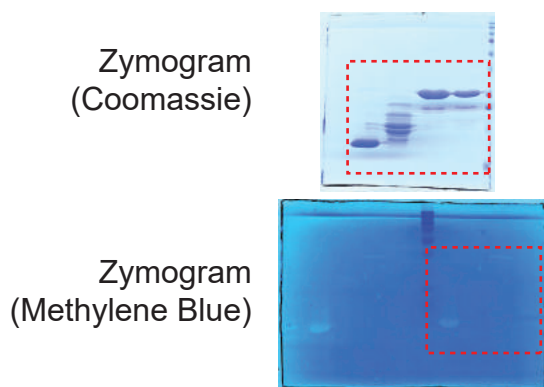

Figure S5

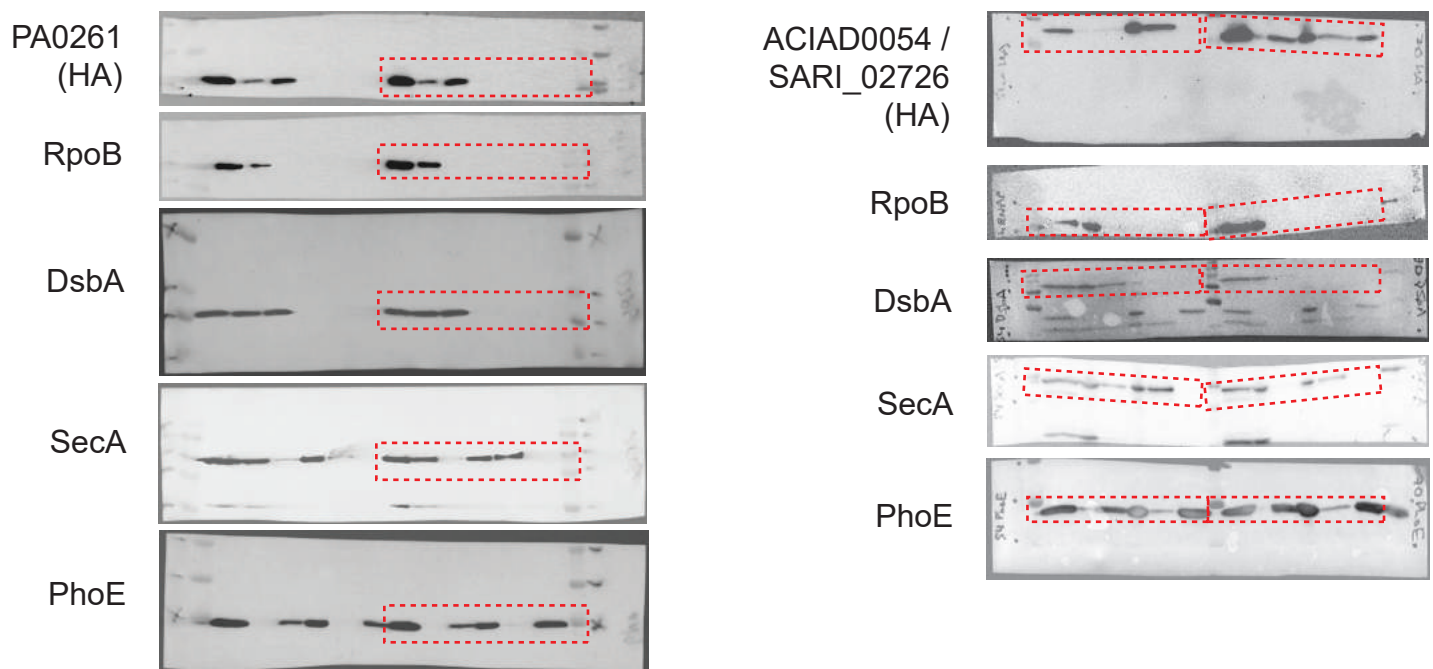

Supplement: Data S1. All Uncropped Immunoblots and Coomassie-Stained SDS-PAGE Gels, Related to Figures 1, 5, 7, S1, S3, S5, and S6 — Red dashed boxes demarcate where the images have been cropped. [file mmc3.pdf]
