# Supplementary material for: The Pseudomonas aeruginosa T6SS Delivers a Periplasmic Toxin that Disrupts Bacterial Cell Morphology
Source: Cell Rep. 2019 Oct 1;29(1):187–201.e7. doi: 10.1016/j.celrep.2019.08.094 (PMC6899460; doi:10.1016/j.celrep.2019.08.094)
Supplement: Document S2. Article plus Supplemental Information [file mmc4.pdf]

Cell Reports

Article

The *Pseudomonas aeruginosa* T6SS Delivers a Periplasmic Toxin that Disrupts Bacterial Cell Morphology

Graphical Abstract

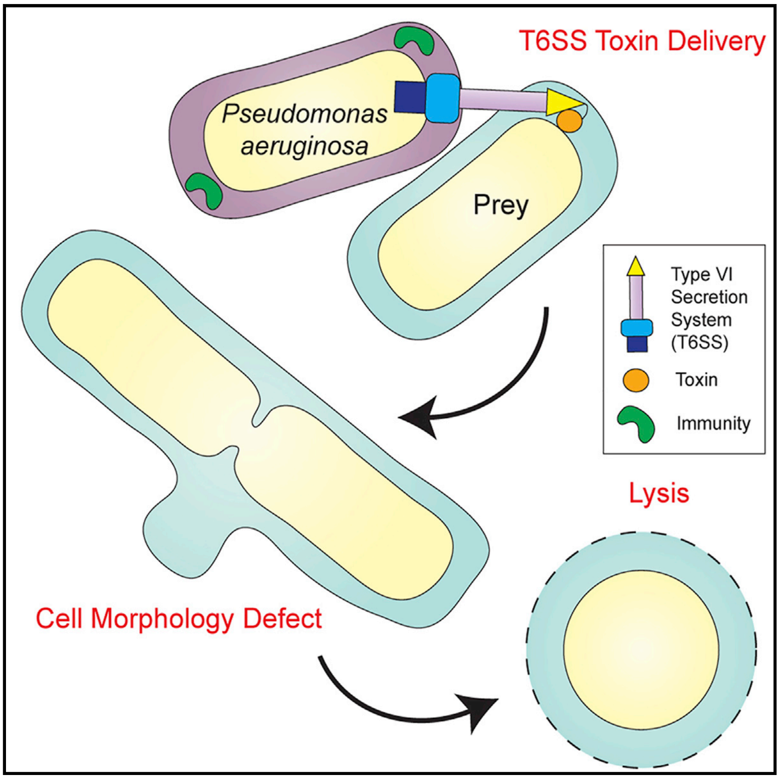

Highlights

- The structure of the VgrG2b C-terminal domain presents a metallopeptidase fold
- VgrG2b exerts antibacterial activity in the periplasmic space
- Toxicity of VgrG2b is counteracted by a cognate periplasmic immunity protein
- VgrG2b$_{C\text{-}ter}$-intoxicated prey cells bleb at the midcell and lyse

Authors

Thomas E. Wood, Sophie A. Howard, Andreas Förster, ..., Waldemar Vollmer, Paul S. Freemont, Alain Filloux

Correspondence

a.filloux@imperial.ac.uk

In Brief

The bacterial type VI secretion system (T6SS) delivers effector proteins into prokaryotic and eukaryotic cells to enhance the survival of the donor cell. Wood et al. describe an antibacterial T6SS toxin family eliciting a profound cell division defect and lysis. The structure of this periplasmic-acting toxin reveals a metallopeptidase fold.

Wood et al., 2019, Cell Reports *29*, 187–201
October 1, 2019 © 2019 The Authors.
https://doi.org/10.1016/j.celrep.2019.08.094

CellPress

OPEN ACCESS
CellPress

The *Pseudomonas aeruginosa* T6SS Delivers a Periplasmic Toxin that Disrupts Bacterial Cell Morphology

Thomas E. Wood,[1,7,8] Sophie A. Howard,[1,12] Andreas Förster,[2,9,12] Laura M. Nolan,[1] Eleni Manoli,[1] Nathan P. Bullen,[3,4] Hamish C.L. Yau,[5] Abderrahman Hachani,[1,10,11] Richard D. Hayward,[6] John C. Whitney,[3,4] Waldemar Vollmer,[5] Paul S. Freemont,[2] and Alain Filloux[1,13,*]
[1]MRC Centre for Molecular Bacteriology and Infection, Department of Life Sciences, Imperial College London, London SW7 2AZ, UK
[2]Section of Structural Biology, Department of Medicine, Imperial College London, London SW7 2AZ, UK
[3]Michael DeGroote Institute for Infectious Disease Research, McMaster University, Hamilton, ON L8S 4K1, Canada
[4]Department of Biochemistry and Biomedical Sciences, McMaster University, Hamilton, ON L8S 4L8, Canada
[5]Centre for Bacterial Cell Biology, Institute for Cell and Molecular Biosciences, Newcastle University, Newcastle upon Tyne NE2 4HH, UK
[6]Division of Microbiology and Parasitology, Department of Pathology, University of Cambridge, Cambridge CB2 1QP, UK
[7]Present address: Department of Medicine, Division of Infectious Diseases, Massachusetts General Hospital, Boston, MA, USA
[8]Present address: Department of Microbiology, Blavatnik Institute, Harvard Medical School, Boston, MA, USA
[9]Present address: DECTRIS Ltd., Täfernweg 1, 5405 Baden-Dättwil, Switzerland
[10]Present address: Department of Pathogen Molecular Biology, Faculty of Infectious and Tropical Diseases, London School of Hygiene and Tropical Medicine, London WC1E 7HT, UK
[11]Present address: Department of Microbiology and Immunology, University of Melbourne at the Peter Doherty Institute for Infection and Immunity, Melbourne, VIC 3000, Australia
[12]These authors contributed equally
[13]Lead Contact
*Correspondence: a.filloux@imperial.ac.uk
https://doi.org/10.1016/j.celrep.2019.08.094

SUMMARY

The type VI secretion system (T6SS) is crucial in interbacterial competition and is a virulence determinant of many Gram-negative bacteria. Several T6SS effectors are covalently fused to secreted T6SS structural components such as the VgrG spike for delivery into target cells. In *Pseudomonas aeruginosa*, the VgrG2b effector was previously proposed to mediate bacterial internalization into eukaryotic cells. In this work, we find that the VgrG2b C-terminal domain ($VgrG2b_{C\text{-}ter}$) elicits toxicity in the bacterial periplasm, counteracted by a cognate immunity protein. We resolve the structure of $VgrG2b_{C\text{-}ter}$ and confirm it is a member of the zinc-metallopeptidase family of enzymes. We show that this effector causes membrane blebbing at midcell, which suggests a distinct type of T6SS-mediated growth inhibition through interference with cell division, mimicking the impact of β-lactam antibiotics. Our study introduces a further effector family to the T6SS arsenal and demonstrates that VgrG2b can target both prokaryotic and eukaryotic cells.

INTRODUCTION

Protein secretion systems are used by bacteria to interact with other organisms and exploit the local environment (Filloux, 2011). Proteins transported by these systems shape the behavior of polymicrobial communities and interfere with eukaryotic cells. The type VI secretion system (T6SS) delivers effector proteins into both bacterial and eukaryotic targets in a contact-dependent manner (Ho et al., 2014; Jiang et al., 2014) and has been proposed to be involved in the acquisition of public goods such as metal ions (Lin et al., 2017; Si et al., 2017a, 2017b). Although the T6SS can manipulate signaling pathways and the cytoskeleton of eukaryotes (Aubert et al., 2016; Chen et al., 2017; Hachani et al., 2016), it appears to be predominantly involved in bacterial antagonism, in which toxin delivery into neighboring cells elicits growth inhibition and cell death (Allsopp et al., 2017; Hachani et al., 2014; MacIntyre et al., 2010; Russell et al., 2014).

The T6SS is a contractile injection system anchored in the bacterial envelope, evolutionarily related to bacteriophage tails (Leiman et al., 2009; Pukatzki et al., 2007). A conformational change of the T6SS baseplate platform is thought to trigger the contraction of a cytoplasmic sheath, expelling a spear-like structure to puncture a target cell membrane (Cianfanelli et al., 2016; Salih et al., 2018; Wang et al., 2017). The spear is composed of a stack of Hcp rings capped with a spike complex of a trimer of VgrG proteins and a PAAR protein tip (Mougous et al., 2006; Renault et al., 2018; Shneider et al., 2013). The trimeric VgrG complex shares extensive structural homology with the bacteriophage gp27-gp5 tail spike, which also serves as a membrane puncturing device (Hachani et al., 2011; Leiman et al., 2009; Pukatzki et al., 2007). The spear is decorated with cargo effector proteins, often associating through non-covalent interactions with the structural components (Cianfanelli et al., 2016; Jiang

This is an open access article under the CC BY license (http://creativecommons.org/licenses/by/4.0/).

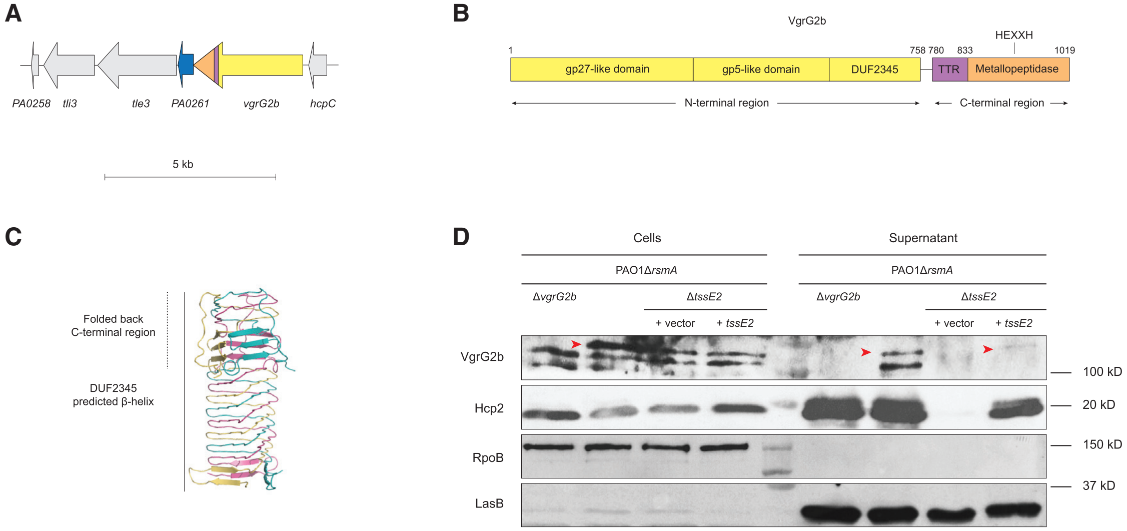

Figure 1. H2-T6SS-Dependent Secretion of the Evolved VgrG2b

(A) *vgrG2b* locus of *P. aeruginosa* PAO1. The *vgrG2b* gene is colored according to its domain architecture shown in (B). *PA0261*, shown in blue, is also characterized in this work. Scale bar represents 5 kb.

(B) Schematic of the domain organization of the VgrG2b protein. The N-terminal region (yellow) forms the spike-like structure of the T6SS spear, composed of the gp27- and gp5-like domains and likely the DUF2345 domain. The TTR domain is a putative protein-protein interaction domain. The C-terminal domain has an HEXXH motif typically found in the catalytic site of metallopeptidases.

(C) Model of the tertiary structure of the DUF2345 domain of the VgrG2b trimer, with the protomers depicted in red, yellow, and teal.

(D) Immunoblot demonstrating secretion of VgrG2b by the H2-T6SS. Red arrows indicate the band corresponding to the VgrG2b protein to distinguish it from the non-specific bands recognized by the polyclonal antibody. Anti-RpoB recognizes the β subunit of RNA polymerase and is employed as a lysis control, while anti-LasB detects a secreted type II secretion system (T2SS) effector protein, used as a supernatant loading control. Image is representative of three independent experiments.

Uncropped immunoblots and gels of all figures are provided in Data S1.

et al., 2019; Shneider et al., 2013). Besides these cargo effectors, effector domains can be fused to a Hcp, VgrG, or PAAR protein. Examples of these specialized effectors, also called evolved spear components, include the NAD(P)$^+$-hydrolyzing toxin Tse6 from *Pseudomonas aeruginosa* or the peptidoglycan hydrolase extension of VgrG3^{VC} of *Vibrio cholerae* (Brooks et al., 2013; Hachani et al., 2014; Whitney et al., 2015).

P. aeruginosa possesses three T6SSs, designated H1-, H2-, and H3-T6SS (Mougous et al., 2006). Although the H1-T6SS is well characterized as an antibacterial weapon, our understanding of the effectors secreted by the H2-T6SS is still in its infancy. Two phospholipases, PldA and Tle4, are secreted by this system into both bacterial and eukaryotic cells and have consequently been designated trans-kingdom effectors (Jiang et al., 2014, 2016). In addition, the antibacterial nuclease effector TseT and the evolved VgrG2b have been linked to this system (Burkinshaw et al., 2018; Sana et al., 2015). VgrG2b is involved in the internalization of *P. aeruginosa* PAO1 into epithelial cells, because uptake of a *vgrG2b* mutant is decreased versus the wild-type strain (Sana et al., 2015). Infection of epithelial cells expressing *vgrG2b* displays enhanced internalization not only of wild-type *P. aeruginosa* but also of an H2-T6SS-deficient strain. Elements of the host cytoskeleton are co-opted for invasion, because chemical inhibition of actin and microtubule polymerization blocks bacterial uptake. Furthermore, interactome analysis of VgrG2b ectopically expressed in host cells identified components of the γ-tubulin ring complex (γTuRC) as binding partners of this evolved spike protein. Despite the microtubule network being implicated in *P. aeruginosa* invasion, the mechanism of this process remains to be understood.

In this study, we report that VgrG2b represents an evolved trans-kingdom T6SS effector. We provide evidence that VgrG2b is directly secreted by the H2-T6SS and that the VgrG2b C-terminal domain (VgrG2b$_{C\text{-}ter}$) possesses antibacterial activity. We show that this domain, the structure of which we present in this work, is a member of a widespread family of metallopeptidases eliciting toxicity in the bacterial periplasm and that it can be neutralized by a cognate immunity protein. The toxicity of VgrG2b$_{C\text{-}ter}$ results in profound morphological anomalies characterized by blebbing of the bacterial membrane at the site of septation, a phenotype reminiscent of inhibition of the cell division machinery by β-lactam antibiotics.

RESULTS

VgrG2b Is an Evolved VgrG Protein Secreted by the H2-T6SS

VgrG2b is encoded in the *hcpC* locus with the hypothetical protein PA0261 and the effector-immunity module Tle3-Tli3 (type VI lipase effector 3-type VI lipase immunity 3) (Figure 1A), where Tle3 is a predicted phospholipase (Barret et al., 2011; Wood et al., 2019). As such, it is not genetically linked to any T6SS core gene clusters. Beyond its canonical trimeric spike-forming

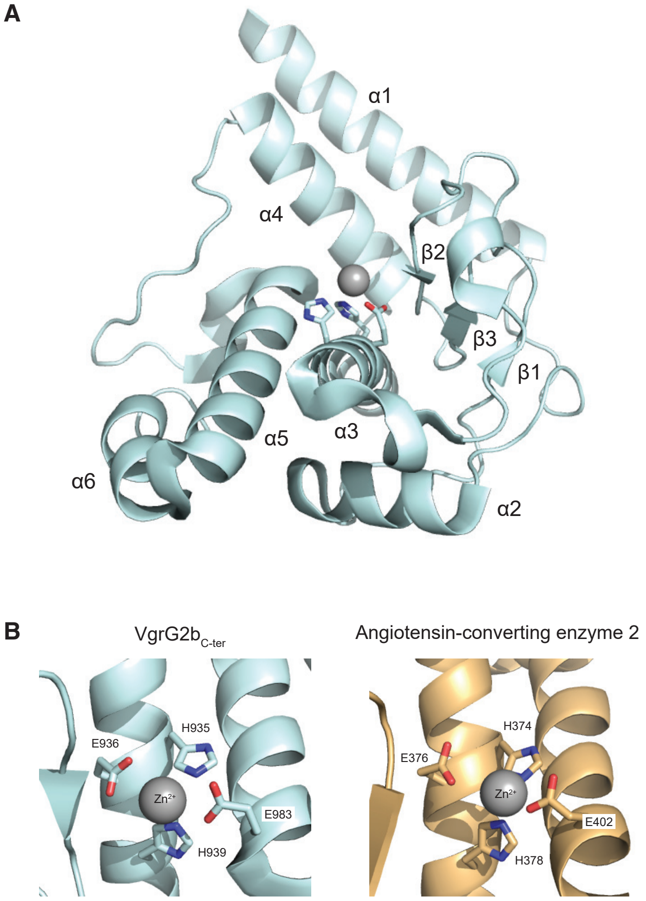

Figure 2. Structure of the VgrG2b Metallopeptidase Domain

(A) Cartoon representation of the metallopeptidase fold of the VgrG2b C-terminal domain. The zinc ion is modeled in the catalytic center as a gray sphere. Elements of secondary structure are labeled (α, α helix; β, β strand) and the three catalytic residues are shown as sticks. See also Figure S1 and Tables S1 and S2.

(B) Comparison of the active site of the VgrG2b metallopeptidase domain (left) with that of angiotensin-converting enzyme 2 (right) from *Homo sapiens* (PDB: 3D0G). The coordinating residues of the gray zinc ion are labeled. In VgrG2b$_{C\text{-ter}}$, H935, E936, and H939 form the catalytic triad, and E983 is the additional ligand; the corresponding residues in human angiotensin-converting enzyme 2 are H374, E375, H378, and E736, respectively.

region containing gp27- and gp5-like domains, VgrG2b harbors a C-terminal extension, rendering it an evolved VgrG protein (Figure 1B). VgrG2b also contains a DUF2345 domain, predicted to adopt a β-helical fold similar to the gp5-like domain (Figure 1C), which can be considered an extension of the spike region (Sana et al., 2015). The C-terminal portion of the VgrG2b spike protein is predicted to function as a metallopeptidase because of the presence of a signature zinc-binding HEXXH motif (Figure 1B). Bioinformatic analysis of the linker region between the DUF2345 and the metallopeptidase domains reveals a transthyretin (TTR)-like fold, implicated in protein-protein interactions. Indeed, C-terminal TTR folds have been described in PAAR and VgrG proteins, with that of VgrG1 in enteroaggregative *Escherichia coli* empirically shown to be important in the delivery of the cargo effector Tle1 (Flaugnatti et al., 2016; Shneider et al., 2013).

A previous study determined that VgrG2b and the H2-T6SS are required in invasion of epithelial cells during infection, but no H2-T6SS-dependent secretion of VgrG2b was effectively demonstrated (Sana et al., 2015). We have recently described *in vitro* conditions in which the H2-T6SS is active and established that the Hcp2 tail tube protein, encoded by at least one of the identical paralogs *hcpA*, *hcpB*, *hcpC*, and *hcp2* in *P. aeruginosa* PA14, is secreted by this system (Allsopp et al., 2017). Here, we employed antibodies raised against peptides within the metallopeptidase domain of VgrG2b to probe for this spike protein in the supernatant of *P. aeruginosa* cultures grown in H2-T6SS-conducive conditions. VgrG2b and Hcp2 are detected in the extracellular milieu of the parental strain (PAO1Δ*rsmA*); however, deletion of the *tssE2* gene, encoding an H2-T6SS baseplate component, abolishes their secretion (Figure 1D). Complementation with *tssE2 in trans* partially restores secretion of VgrG2b and Hcp2.

Altogether, this confirms that VgrG2b is secreted in an H2-T6SS-dependent manner. Although the *H2-T6SS* locus of *P. aeruginosa* PAO1 lacks genes encoding the spike and tube proteins of the T6SS spear assembly, this result highlights that the H2-T6SS supports the secretion of several of distally encoded effectors (Barret et al., 2011; Hachani et al., 2011).

Crystal Structure of VgrG2b C-Terminal Effector Domain

Next, we purified VgrG2b$_{C\text{-ter}}$ (residues 770–1,019) to initiate structural analysis. We solved the structure of VgrG2b$_{C\text{-ter}}$, encompassing residues 833–1,019, using crystals of both the native and the selenomethionine-substituted forms with resolutions of 3.2 and 3.0 Å, respectively (Figure 2A). Two copies of VgrG2b$_{C\text{-ter}}$ were found in the asymmetric unit of the crystal, which could be superimposed with a root-mean-square deviation (RMSD) of 0.1 Å (Tables S1 and S2). In solution, however, the purified metallopeptidase domain exists predominantly as a monomer, as determined by size-exclusion chromatography-multi-angle laser light scattering (SEC-MALLS), with a dimeric species also present (Figure S1A). It is likely that the physiologically relevant oligomerization state of the metallopeptidase domain is achieved only in the full-length VgrG2b protein.

The VgrG2b$_{C\text{-ter}}$ protomer possesses a single domain composed of six α helices and three β strands (Figure 2A), forming a shallow bowl with multiple small loops. A PDB search using the DALI server identified several thermolysin-like metallopeptidases as structural homologs of the VgrG2b$_{C\text{-ter}}$ effector domain (Holm and Laakso, 2016). One such metallopeptidase is human angiotensin-converting enzyme 2 (*Z* score: 5.0, C_α RMSD of 4.2 Å across 105 residues), with the active sites of these proteins displaying similar architectures (Figure 2B). Although metallopeptidases target diverse substrates, the overall core fold of three α helices (α2, α3, and α5) and two β strands (β2 and β3) is highly conserved and bears the classical HEXXH catalytic motif typical of these zinc-dependent enzymes, where X is any amino acid (Figure 2B).

Indeed, the conformations of the HEXXH residues in our structure are suggestive of metal ion coordination, and the geometry

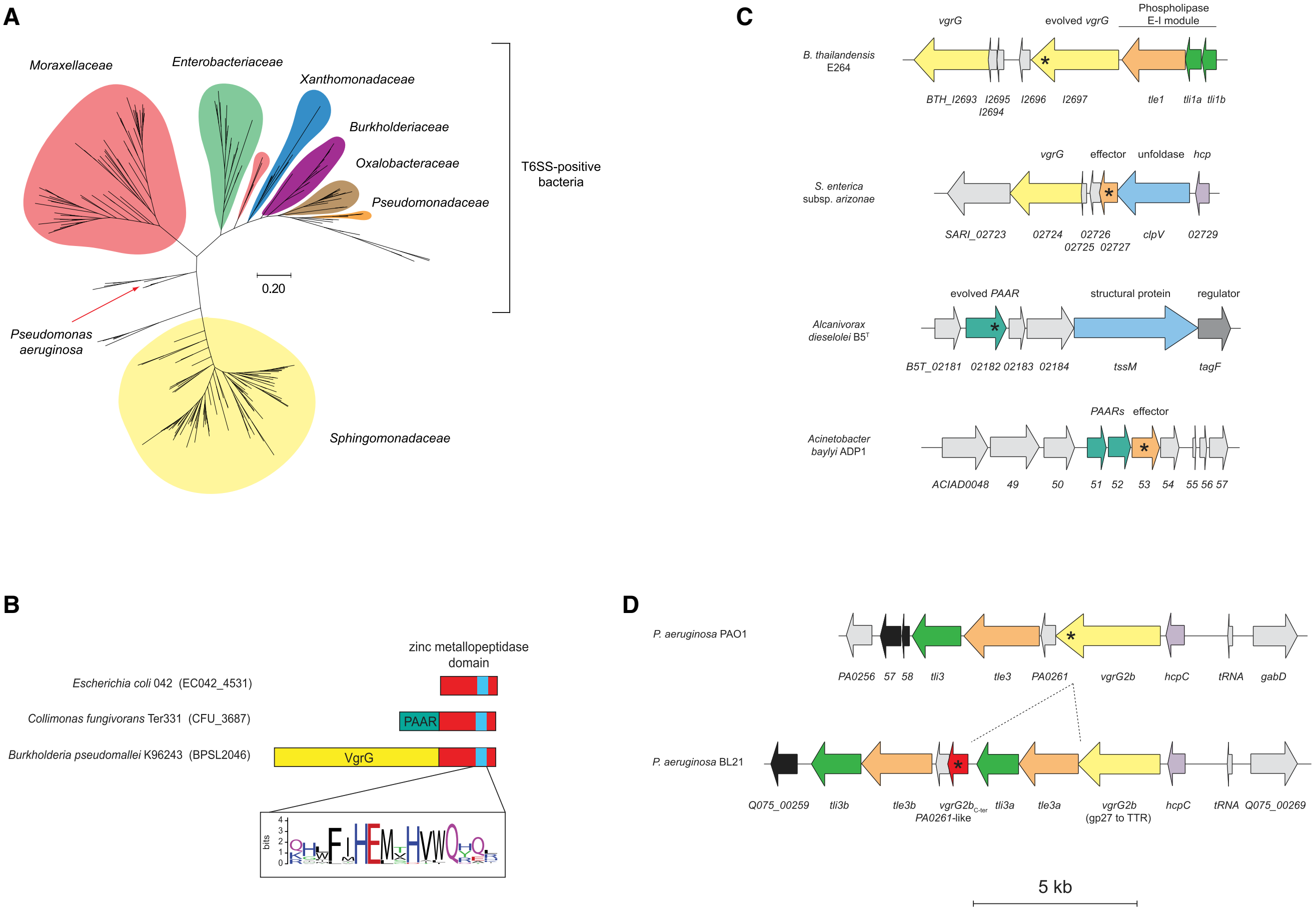

Figure 3. Phylogenetic Analysis of the VgrG2b Metallopeptidase Domain

(A) Phylogenetic tree of 240 homologs of the VgrG2b metallopeptidase domain constructed using the maximum likelihood method. Clades are colored by bacterial family, and the branch denoting VgrG2b of *P. aeruginosa* is labeled with a red arrow. The scale represents the number of residue substitutions per site.

(B) Schematic representation of the domain architecture of various VgrG2b metallopeptidase domain orthologs. The sequence logo shows the conservation of residues constituting the predicted HEXXH catalytic motif.

(C) Diagram of genetic cassettes encoding VgrG2b metallopeptidase homologs (shown with an asterisk) found within T6SS loci. In each case, the gene encoding the putative metallopeptidase lies upstream of a small ORF. The VgrG2b$_{C\text{-}ter}$ homolog in *B. thailandensis* is the evolved VgrG BTH_I2697; SARI_02727 of *S. arizonae* and ACIAD0053 of *Acinetobacter baylyi* are predicted cargo effectors, whereas in *Alcanivorax dieselolei*, B5T_02182 is a putative evolved PAAR protein.

(D) Schematic of the genetic loci of the *vgrG2b* satellite islands of *P. aeruginosa* strains PAO1 and BL21. The site of a possible recombination event suggesting duplication of the *tle3-tli3* module within the 3′ end of *vgrG2b* in *P. aeruginosa* BL21 is shown by dotted lines. The gene encoding the VgrG2b$_{C\text{-}ter}$ metallopeptidase is marked by an asterisk. Scale bar represents 5 kb.

suggests that the electron density in the putative metal-binding site corresponds to a divalent cation such as zinc. To confirm that VgrG2b$_{C\text{-}ter}$ binds zinc, we employed differential scanning fluorimetry to assess the thermodynamic stabilization of the metallopeptidase in the presence of zinc ions. The melting temperature of the domain increases from 46.3°C in the absence of zinc to a maximum of 55.1°C in the presence of an eight-fold excess of the metal ion (Figure S1B), providing a strong indication of the binding of zinc to the metallopeptidase. The assigned zinc ion is coordinated by the two histidine residues of the catalytic triad and has a third glutamate ligand (E983) at the top of helix α5 (Figure 2B). These features permit classification of VgrG2b$_{C\text{-}ter}$ as a thermolysin-like gluzincin within the MA(E) metallopeptidase subclan (Rawlings et al., 2018).

VgrG2b$_{C\text{-}ter}$ Is Part of a Wider Family of Metallopeptidase Effectors

We assessed the prevalence of the VgrG2b$_{C\text{-}ter}$ metallopeptidase domain by exploring its phylogenetic distribution. Homologous metallopeptidases were found throughout the proteobacterial phylum, especially in bacteria possessing a T6SS (Li et al., 2015) (Figure 3A). Intriguingly, the presence of VgrG2b$_{C\text{-}ter}$-like proteins in *Sphingomonadaceae*, which rarely encode a T6SS, implies that this protein family may have a role beyond constituting a T6SS effector. Nevertheless, many orthologs are encoded within T6SS gene clusters and are classified as DUF4157 domain-containing putative metallopeptidases, found as both putative cargo and specialized effectors. For example, *Burkholderia pseudomallei* encodes an evolved VgrG with this

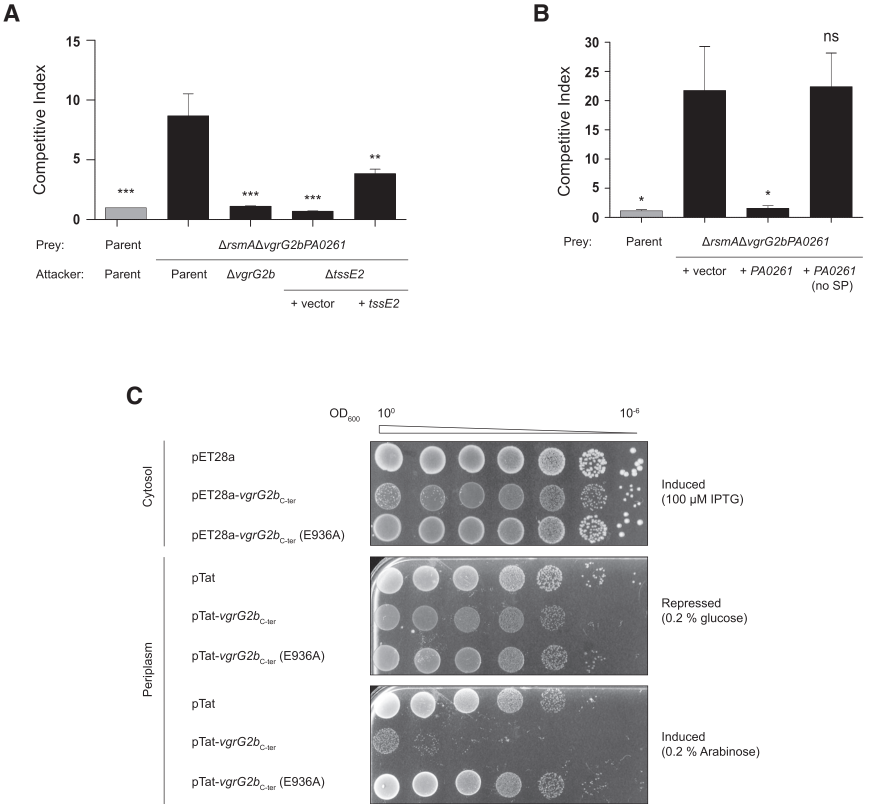

Figure 4. VgrG2b$_{C\text{-ter}}$ Is the Periplasmic Toxin of an Antibacterial Effector-Immunity Pair

(A) Intraspecies *P. aeruginosa* competition assay between a strain lacking the *vgrG2b-PA0261* module and various isogenic attacker strains under H2-T6SS-conducive growth conditions at 25°C for 8 h. The competition assay between the parental strain (PAO1Δ*rsmA*) and itself, shown in gray, is the internal control for competitive parity. For (A) and (B), the strains used are described in the text. The values and error bars represent the mean ± SEM (n = 3 biological replicates). Statistical comparisons undertaken used one-way ANOVA with Dunnett's test using the parent versus prey competition as the comparator (*$p < 0.05$, **$p < 0.01$, ***$p < 0.001$; ns, not significant).

(B) Intraspecies competition assay showing that PA0261 is the immunity protein for VgrG2b$_{C\text{-ter}}$. The prey strain produces PA0261 *in trans* with or without its native signal peptide (PA0261(no SP)) or harbors the empty vector. The attacker is the PAO1Δ*rsmA* parental strain. See also Figure S2.

(C) Survival of *E. coli* expressing *vgrG2b*$_{C\text{-ter}}$ or its catalytically inactive mutant *vgrG2b*$_{C\text{-ter}}$ (E936A). The proteins are targeted to the periplasm by the Tat-dependent signal peptide of TorA in the pTat vector. Ten-fold serial dilutions of cultures (optical density 600 [OD_{600}] 10^0 to 10^{-6}) were spotted on LB agar containing the noted concentrations of repressor (glucose) or inducer (IPTG for pET28a and arabinose for pTat) and grown for 24 h. Image is representative of three independent experiments.

metallopeptidase domain, similar to VgrG2b. Yet in *Collimonas* and *Alcanivorax* species, it is grafted as an extension of a PAAR protein. Finally, many members of the *Enterobacteriaceae* family code for this metallopeptidase as a putative T6SS cargo effector (Figures 3B and 3C), underscoring the modularity of T6SS effector proteins.

Further analysis of the distribution of *vgrG2b* genes uncovered evidence for a remarkable recombination event in the *P. aeruginosa* BL21 strain, in which the *tle3-tli3* module is duplicated within the 3′ end of the *vgrG2b* gene (Figure 3D). This has given rise to a canonical *vgrG* gene encoding VgrG2b up to its TTR fold, with the metallopeptidase domain encoded by a separate open reading frame (ORF) downstream of the phospholipase effector-immunity locus. This genetic reorganization also implies that *vgrG* genes can acquire or lose their 3′ end through recombination, allowing the exchange of effector domains at the VgrG tip. This is reminiscent of observations concerning C-terminal toxin modules in systems such as contact-dependent growth inhibition (CDI) and Rhs proteins (Ruhe et al., 2017; Wang et al., 1998). Overall, these *in silico* analyses suggest that VgrG2b$_{C\text{-ter}}$ is frequently associated with proteobacterial T6SS loci and that its secretion can be achieved via various effector configurations.

VgrG2b-PA0261 Constitutes an Antibacterial Effector-Immunity Pair

Our bioinformatic analysis of the genetic context of T6SS-associated VgrG2b$_{C\text{-ter}}$ orthologs revealed intriguing synteny of this effector gene with a small ORF, often unannotated and encoded downstream, such as *PA0261* in *P. aeruginosa* (Figure 1A), *BTH_I2696* in *Burkholderia thailandensis*, *ACIAD0054* in *Acinetobacter baylyi*, *SARI_02726* in *Salmonella enterica* subsp. *arizonae*, or *B5T_02183* in *Alcanivorax dieselolei* (Figure 3C). The juxtaposition of these genes is invariable, prompting the hypothesis that *P. aeruginosa* VgrG2b$_{C\text{-ter}}$ may constitute an antibacterial effector-immunity pair with the *PA0261* gene product. Indeed, T6SS effectors whose genes are systematically encoded in tandem with a small ORF are frequently found to exert antibacterial activity, with the adjacent gene encoding its cognate immunity protein (English et al., 2012; Hood et al., 2010; Russell et al., 2011).

We engineered a strain lacking the *vgrG2b-PA0261* genes and placed it in competition with the parental strain under conditions in which the H2-T6SS is active. Whereas competition of the parent with itself results in competitive parity, the Δ*vgrG2bPA0261* strain exhibits a significant growth disadvantage (Figure 4A). VgrG2b is responsible for the advantage of the parental strain, because a Δ*vgrG2b* attacker no longer outcompetes the Δ*vgrG2bPA0261* prey. Importantly, the attacker and prey strains are isogenic; thus, the *vgrG2b-PA0261* module represents the sole difference between these strains. As such, the prey strain is immune to the action of all other T6SS toxins such as Tle3, encoded downstream of *PA0261* (Figure 1A). Deletion of *tssE2* in the attacker strain, rendering the H2-T6SS non-functional, restores competitive parity of the prey strain,

confirming the H2-T6SS-dependent secretion of VgrG2b (Figure 4A), whereas complementation of *tssE2* on a plasmid partially restores the growth advantage of the attacker strain. Furthermore, a *P. aeruginosa* strain lacking the immunity gene exhibits a growth defect on solid media, but not in liquid culture, consistent with contact-dependent delivery by the H2-T6SS (Figures S2A and S2B).

To assess the role of PA0261 as the immunity protein neutralizing VgrG2b-mediated toxicity, we introduced the *PA0261* gene into the Δ*vgrG2bPA0261* strain *in trans*. Constitutive expression of *PA0261* in the prey strain abolishes the competitive growth advantage of the attacker, whereas the empty vector provides no such immunity (Figure 4B). Bioinformatic analysis of the PA0261 sequence revealed the presence of a putative N-terminal signal peptide, likely directing the protein into the periplasm (Figure S2C). Provision of *PA0261* without the sequence encoding this N-terminal portion (PA0261(no SP)) fails to prevent elimination by the parent (Figure 4B), suggesting that the predicted extracytoplasmic localization of this protein is needed to neutralize VgrG2b, similar to the immunity proteins of other *P. aeruginosa* periplasmic-acting toxins (Jiang et al., 2016; Russell et al., 2011).

If PA0261 requires its putative N-terminal signal peptide to act as an immunity protein, VgrG2b must elicit its toxicity beyond the cytoplasmic membrane. We ectopically produced VgrG2b$_{C\text{-ter}}$ in *E. coli* and targeted the domain to the periplasm by fusing it to an N-terminal signal peptide from the Tat-dependent substrate TorA (Palmer and Berks, 2012; Santini et al., 2001). Production of VgrG2b$_{C\text{-ter}}$ is not detrimental to *E. coli* when the protein remains in the cytoplasm, because this strain grows similarly to vector control strain (Figure 4C). However, this domain exhibits acute toxicity when targeted to the periplasmic compartment by the Tat-dependent signal peptide. Crucially, substitution of the putative catalytic glutamic acid residue to an alanine (E936A from HEXXH) renders VgrG2b$_{C\text{-ter}}$ non-toxic in the periplasm, restoring growth. No differences in the levels of VgrG2b$_{C\text{-ter}}$ production are observed between the periplasmic-targeted toxin and its inactive isoform (Figure S2D), indicating that the putative enzymatic activity of the VgrG2b$_{C\text{-ter}}$ metallopeptidase is responsible for toxicity. Mutation of the two histidine residues (H935A and H939A) coordinating the zinc ion in our structure also abolish periplasmic toxicity, supporting our designation of the HEXXH catalytic triad (Figure S2E). Interestingly, although raising the tonicity of media has been reported to rescue growth of bacteria producing the periplasmic-acting T6SS effectors Tse1 and Tse3, the addition of 0.5 M sucrose does not suppress the growth defect of *E. coli* producing VgrG2b$_{C\text{-ter}}$ in the periplasm (Russell et al., 2011) (Figure S2F). This suggests that VgrG2b$_{C\text{-ter}}$ may elicit an activity distinct from the D,L-endopeptidase and muramidase activities of Tse1 and Tse3, respectively (described later).

We sought to determine whether toxicity was displayed by orthologs of VgrG2b$_{C\text{-ter}}$ and expressed the putative T6SS cargo effector ACIAD0053 from *A. baylyi* similarly. Surprisingly, induction of periplasmic-targeted ACIAD0053 expression does not hamper *E. coli* growth under identical conditions to that of intoxication by VgrG2b$_{C\text{-ter}}$ (Figure S2G). Nevertheless, reduction of the osmolarity of the medium (low-salt LB [LB-LS]) to increase bacterial susceptibility to lysis results in a drastic reduction in growth of the strain expressing pTat-*ACIAD0053*. Once more, alanine substitution of the residues of the catalytic triad (*ACIAD0053**) produces a non-toxic isoform (Figures S2G and S2H). These results indicate that members of the VgrG2b$_{C\text{-ter}}$ metallopeptidase effector family act as potent periplasmic antibacterial toxins.

Functional Characterization of the PA0261 Immunity Protein Family

To validate the toxin-immunity relationship of the VgrG2b$_{C\text{-ter}}$-PA0261 pair, we investigated whether PA0261 could protect *E. coli* from VgrG2b$_{C\text{-ter}}$-mediated toxicity. Coexpression of the immunity gene relieves the growth defect caused by the metallopeptidase (Figure 5A). As a specificity control, we expressed *tli4*, encoding the cognate immunity protein of the antibacterial H2-T6SS-dependent effector Tle4, which was unable to restore bacterial viability (Jiang et al., 2016). Immunoblot analysis determined that both immunity proteins are produced (Figure S3A). This demonstrates that PA0261 is the cognate immunity determinant of the VgrG2b$_{C\text{-ter}}$ toxin. Furthermore, the additional presence of a higher molecular weight band for PA0261-HA is indicative of the precursor form of a periplasmic protein, supporting the predicted localization of this immunity protein, which we later confirm (Figures S3A and S5A). We then used far-western dot blotting (Figure 5B) to investigate whether complex formation with the metallopeptidase domain was the neutralization mechanism of PA0261. We spotted recombinant VgrG2b$_{C\text{-ter}}$ (Figure S3B) or the C-terminal TTR domain of VgrG4b as bait proteins on nitrocellulose membrane and incubated them with a bacterial lysate overproducing hemagglutinin (HA)-tagged immunity proteins PA0261 or Tli3. Immunoblotting shows that despite production of both immunity proteins, only PA0261-HA binds to VgrG2b$_{C\text{-ter}}$, and not to VgrG4b$_{C\text{-ter}}$, suggesting a specific interaction with the metallopeptidase domain (Figure 5B). Furthermore, PA0261 binds the inactive metallopeptidase variant VgrG2b$_{C\text{-ter}}$ (E936A), suggesting that the inactive variant is fully folded (Figures 5B and S3C). This implies that PA0261 neutralizes VgrG2b$_{C\text{-ter}}$ through complex formation, as has been shown for other biochemically characterized T6SS toxin-immunity pairs (Russell et al., 2011; Shang et al., 2012).

In general, immunity proteins of T6SS toxins display little sequence identity with their orthologs, likely because of selective pressure solely on the conservation of toxin binding (Russell et al., 2012). This phenomenon is also apparent in the PA0261 family; however, a multiple sequence alignment of homologous immunity proteins revealed the presence of cysteine residues in partially conserved positions (Figure S4). Because PA0261 is a predicted periplasmic protein, the conservation of cysteine residues engenders the hypothesis that disulfide bond formation may be required for the function of the protein in the oxidizing environment of this compartment. To begin answering this question, we demonstrated through subcellular fractionation that PA0261 is found in the periplasm when expressed in *E. coli* (Figure S5A). We assessed the ability of PA0261 to protect against the toxicity of periplasmic VgrG2b$_{C\text{-ter}}$ in the absence of *dsbA*, encoding the major oxidoreductase of the disulfide bond formation (Dsb) system in *E. coli* (Figure 5C). The levels of PA0261 in the Δ*dsbA* mutant are greatly diminished, hampering the growth

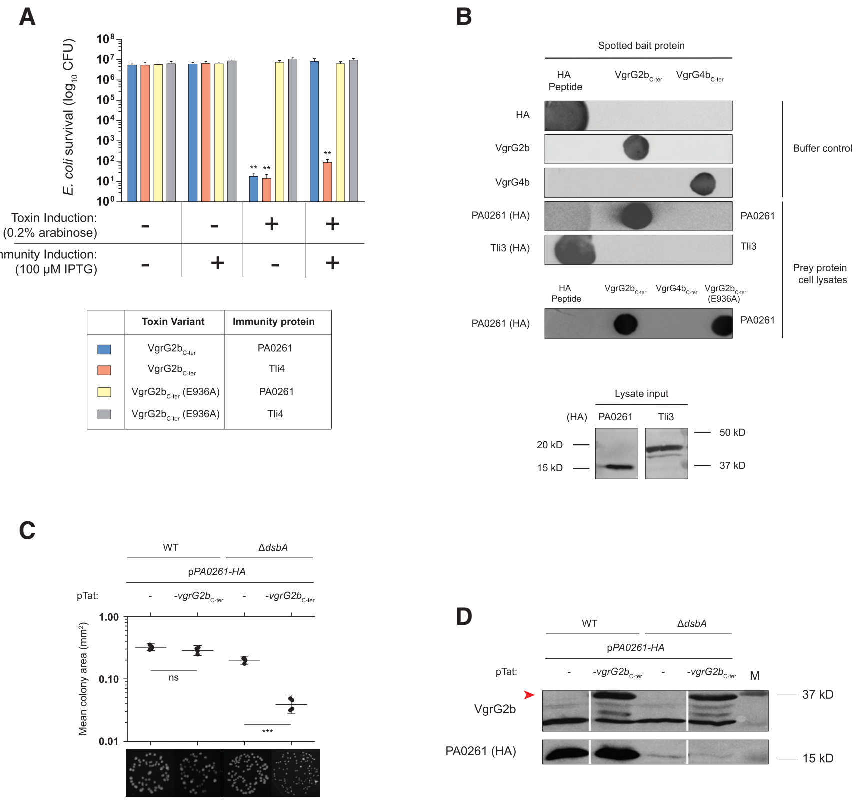

Figure 5. PA0261 Is the Cognate Immunity Protein of VgrG2b$_{C\text{-}ter}$

(A) *PA0261* expression prevents intoxication of *E. coli* cells producing periplasmic VgrG2b$_{C\text{-}ter}$. The non-cognate immunity protein Tli4 is used as a negative control. The values and error bars represent the mean ± SEM (n = 3 biological replicates). Statistical significance of the difference in survival compared with the strain under non-inducing conditions was calculated by one-way ANOVA followed by Tukey's multiple comparison test (**$p < 0.01$). See also Figures S3.

(B) PA0261 interacts with VgrG2b$_{C\text{-}ter}$ in a far-western dot blot assay. Recombinant VgrG2b$_{C\text{-}ter}$, its catalytic mutant, or VgrG4b$_{C\text{-}ter}$ were spotted and incubated with *E. coli* cell lysates containing HA-tagged PA0261 or Tli3. Purified HA peptide is an anti-HA antibody binding control. The bottom panel shows an immunoblot of the lysate input samples confirming immunity protein production. See also Figure S3.

(C and D) Disulfide bond formation is required for PA0261 to efficiently neutralize VgrG2b$_{C\text{-}ter}$. *E. coli* strains produce PA0261-HA with the expression of pTat or pTat-*vgrG2b*$_{C\text{-}ter}$ in the presence or absence of the *dsbA* gene and plated onto solid media. Measurement of colony size and images of the spots are shown in (C). A statistically significant difference between colony size of strains producing periplasmic-targeted VgrG2b$_{C\text{-}ter}$ or not was determined by a two-tailed Student's t test (n = 4 biological replicates; ***$p < 0.001$). Immunoblotting determines production of VgrG2b$_{C\text{-}ter}$ and the HA-tagged PA0261 proteins in (D), where lanes have been excised for clarity. The red arrow shows the position of the VgrG2b$_{C\text{-}ter}$ band. See also Figure S4.

of this strain in the presence of VgrG2b$_{C\text{-}ter}$ (Figures 5C and 5D). This indicates that the Dsb system contributes to the protection of bacterial cells from T6SS-mediated antagonism, in juxtaposition to a recent study demonstrating that a lack of DsbA in prey cells can indirectly confer protection against T6SS attack because of the improper folding of delivered toxins (Mariano et al., 2018). Crystal structures of immunity proteins such as those of the Tai4 family have also revealed the presence of disulfide bonds, extending the relevance of our observation to other families of T6SS immunity proteins (Benz et al., 2013; English et al., 2012; Fukuhara et al., 2018).

In silico examination of putative immunity proteins to members of the VgrG2b$_{C\text{-}ter}$ family suggests that like PA0261, they are targeted to the cell envelope. However, the mechanisms of attaining this localization seem to be diverse. In *Enterobacteriaceae*, PA0261 homologs are predicted to be lipoproteins (Figure S5B), whereas ACIAD0054, the putative immunity protein to ACIAD0053 in *A. baylyi*, appears to bear hallmarks of an N-terminal transmembrane helix (Figure S4, top line). Irrespective of these differences, in both cases the bulk of the immunity protein would be exposed in the periplasm. The second residue following the lipobox cysteine residue of the predicted immunity lipoproteins in *Enterobacteriaceae* is often a glycine, likely directing the protein to the outer membrane rather than constituting an avoidance signal for the lipoprotein outer membrane localization (Lol) pathway as described by the +2 rule (Figure S5B) (Narita and Tokuda, 2017; Yamaguchi et al., 1988). Indeed, membrane fractionation of the *E. coli* cell envelope using selective detergent treatment showed that the PA0261 ortholog from *S. arizonae*, SARI_02726, is enriched in the outer membrane fraction (Figure S5C). Likewise, the *A. baylyi* ortholog ACIAD0054 was enriched in the inner membrane fraction as predicted (Figure S5D). Determination of the localization of members of this immunity protein family supports the designation of VgrG2b$_{C\text{-}ter}$-PA0261 as a periplasmic effector-immunity pair while highlighting the diversity of strategies employed by the immunity determinants to gain access to the periplasm, as summarized in Figure S5E.

VgrG2b$_{C\text{-}ter}$ Is Bacteriolytic but Does Not Display Peptidoglycan Hydrolase Activity

Next, we investigated the activity of the VgrG2b$_{C\text{-}ter}$ effector domain. To determine whether VgrG2b$_{C\text{-}ter}$ induces growth arrest or lysis of target bacteria, we employed an outside-in approach, permeabilizing *E. coli* cells with a sublethal concentration of polymyxin B and measuring whether the toxin reduces culture turbidity. Although VgrG2b$_{C\text{-}ter}$ (E936A) does not decrease the turbidity of the suspension, exogenous addition of lysozyme or VgrG2b$_{C\text{-}ter}$ elicits suspension clarification, thereby showing that the metallopeptidase effector challenges cell integrity, having gained access to the periplasm (Figure 6A).

OPEN
ACCESS
CellPress

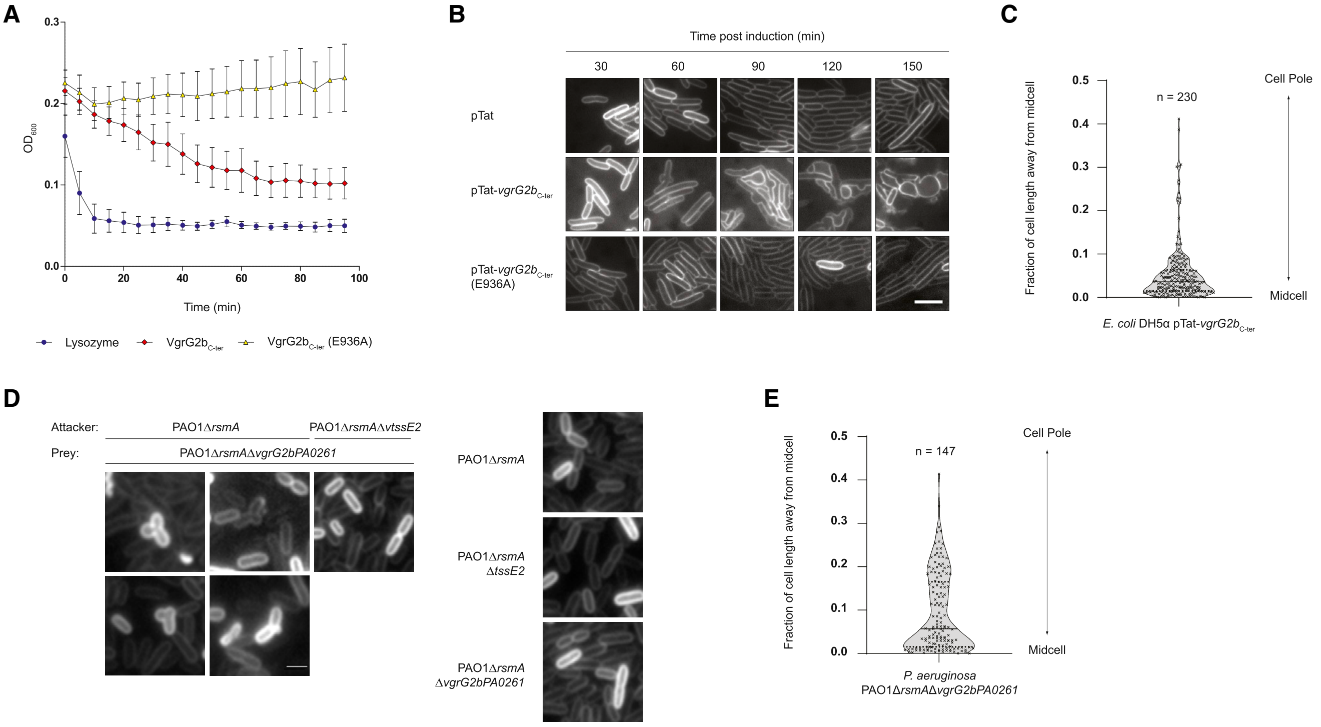

Figure 6. VgrG2b$_{C\text{-}ter}$ Perturbs Cell Division

(A) Incubation of recombinant VgrG2b$_{C\text{-}ter}$ (red), VgrG2b$_{C\text{-}ter}$ (E936A) (yellow) and lysozyme (blue) with polymyxin B-permeabilized *E. coli* cells. A decrease in turbidity indicates bacterial lysis. Points and error bars represent the mean ± SEM (n = 3 biological replicates). See also Figure S6.

(B) Single-cell analysis of *E. coli* cell morphology when expressing pTat, pTat-*vgrG2b*$_{C\text{-}ter}$ or pTat-*vgrG2b*$_{C\text{-}ter}$ (E936A). A series of representative fluorescence microscopy images of bacteria labeled with the membrane dye FM1-43 to highlight the cell exterior at different time points. Scale bar represents 2.5 μm. Image is representative of three independent experiments.

(C) Localization of membrane blebs mediated by VgrG2b$_{C\text{-}ter}$ activity. Using images from three independent time course experiments, typified by those in (B), the position of the membrane bleb of 230 intoxicated bacteria was plotted as its fraction of the cell length from midcell. The solid and dashed lines show the median and quartiles, respectively.

(D) VgrG2b delivery by the H2-T6SS results in membrane blebbing. Fluorescent microscopy images show *P. aeruginosa* strains labeled with FM1-43 either alone (PAO1Δ*rsmA*, PAO1Δ*rsmA*Δ*tssE2*, or PAO1Δ*rsmA*Δ*vgrG2bPA0261*) or in competition (PAO1Δ*rsmA* or PAO1Δ*rsmA*Δ*tssE2* versus PAO1Δ*rsmA*Δ*vgrG2bPA0261*). Representative images from three independent experiments are shown. Scale bar represents 1 μm.

(E) Localization of the site of membrane blebbing of PAO1Δ*rsmA*Δ*vgrG2bPA0261* in competition with the parental strain PAO1Δ*rsmA*, using the dataset represented in (D). Here, 147 intoxicated bacteria were analyzed identically to (C).

Because this bacteriolytic effector is a periplasmic-acting metallopeptidase, we reasoned that it might target either the peptide bonds in the cell wall or a protein within the cell envelope. We probed the action of the toxin toward the bacterial cell wall with various approaches. In a turbidimetric assay, recombinant proteins were incubated with lyophilized *Micrococcus lysodeikticus* as a substrate, but while lysozyme reduces turbidity, VgrG2b$_{C\text{-}ter}$ does not (Figure S6A). Similarly, zymography demonstrated that lysozyme produces a zone of clearing in a stained gel impregnated with purified *E. coli* sacculi, yet no such peptidoglycan degradation was apparent for VgrG2b$_{C\text{-}ter}$ (Figure S6B). An analogous assay for crude protease activity was also used, with a similar negative outcome (Figure S6C). Finally, quantification of muropeptides released through sacculus hydrolysis using high-performance liquid chromatography (HPLC) revealed that treatment with VgrG2b$_{C\text{-}ter}$ does not alter the composition of either tetrapeptide-rich or pentapeptide-rich *E. coli* cell wall preparations (Figures S6D–S6F). Thus, we conclude that VgrG2b$_{C\text{-}ter}$ does not exhibit peptidoglycan hydrolase activity.

VgrG2b Induces Lysis by Apparent Perturbation of Cell Division

Next, we turned to single-cell analysis of *E. coli* producing the periplasmic-targeted metallopeptidase or its inactive variant from the pTat vector. Bacterial morphology was highlighted by staining the membranes with the fluorescent dye FM1-43. Over the course of 2.5 h, no alterations in rod-like *E. coli* cell shape were observed in bacteria harboring the empty vector or pTat-*vgrG2b*$_{C\text{-}ter}$ (E936A). However, production of the active periplasmic metallopeptidase causes aberrant cellular morphology characterized by membrane blebbing, leading to cell rounding and lysis (Figure 6B). Periplasmic VgrG2b$_{C\text{-}ter}$ is still toxic in high-osmolarity conditions (Figure S2F), suggesting that the effector does not cause lysis through global weakening of the cell wall as with Tse1 and Tse3 (Russell et al., 2011). Figure 6C

shows that blebbing predominately originates at midcell, leading to the cracking of the rod shape before rounding up, suggesting a defect in cell division similar to observations during β-lactam antibiotic administration (Chung et al., 2009; Yao et al., 2012). This observation of site-dependent perturbation in cell shape is also in opposition to the notion of VgrG2b$_{C\text{-}ter}$ exhibiting generic peptidoglycan hydrolase activity, unless the metallopeptidase requires a factor or structure specifically localized at the cell septum for its activation.

To discount the possibility that this phenotype is an artifact because of toxin overexpression, we monitored the morphology of *P. aeruginosa* cells subject to VgrG2b-mediated killing in competition assays. Upon attack of the susceptible Δ*vgrG2bPA0261* strain by its parent, we observed similar membrane blebbing, whereas no such morphological aberrations occurred when the attacker strain lacked a functional H2-T6SS (Δ*tssE2*) (Figure 6D). In addition, when the strains were incubated alone, no blebbing occurred. The blebs produced during elimination of VgrG2b-sensitive prey again localized to the septum, indicating that the mechanism of killing by the metallopeptidase domain is similar whether it is delivered by the T6SS or the effector is artificially targeted to the periplasm (Figure 6E). These results demonstrate that VgrG2b$_{C\text{-}ter}$-induced blebbing is a physiologically relevant phenotype and that the target of this toxin is conserved between *E. coli* and *P. aeruginosa*.

Identification of Putative Interacting Partners of VgrG2b$_{C\text{-}ter}$

Because characterization of the biochemical activity of VgrG2b$_{C\text{-}ter}$ had been unsuccessful, we turned to affinity purification-coupled mass spectrometry to investigate the identities of putative target proteins that interact with the toxin in the periplasm. A tagged inactive VgrG2b$_{C\text{-}ter}$ variant was directed to the periplasm after which cellular proteins were crosslinked *in vivo* using dimethyl 3,3′-dithiobispropionimidate (DTBP). Affinity purification pulled down the tagged inactive metallopeptidase and any crosslinked interaction partners. Three independent replicates of the affinity-purified protein samples were subject to mass spectrometry analysis to identify candidate interactors of the metallopeptidase. This preliminary analysis found 89 proteins enriched in the tagged sample dataset compared with the untagged VgrG2b$_{C\text{-}ter}$ control, which were present in at least two of the three replicates. Of these, just 19 proteins (21.3%) were found with MASCOT scores (representing the probability of true identification of the protein) higher than the threshold set to exclude background noise. Twelve of these 19 proteins (63.2%) are localized in the cell envelope and were subsequently considered candidate interactors of VgrG2b$_{C\text{-}ter}$ (Table S3). Three of the four highest-scoring candidates (MltC, PBP5, and PBP6a) are involved in peptidoglycan biogenesis. This suggests that the metallopeptidase may perturb the complex machinery, which maintains the cell wall, inhibiting cell division and producing the observed blebbing at midcell. If VgrG2b$_{C\text{-}ter}$ acts through interference with peptidoglycan remodeling, it may be through direct cleavage of these identified target proteins; however, we cannot exclude that the interaction with periplasmic binding partners stimulates a putative peptidoglycan hydrolase activity of the toxin. The periplasmic action of VgrG2b$_{C\text{-}ter}$ prevents growth of *E. coli* strains with single deletions in *mltC*, *dacA* (encoding PBP5), or *dacC* (encoding PBP6a), indicating that cleavage of one of these enzymes individually is not sufficient for toxicity (Figure S7).

The dataset is also enriched with lipoproteins (8/12, or 67%), raising the possibility that VgrG2b$_{C\text{-}ter}$ might interact with the linker region between the acylated cysteine residue of a lipoprotein and its main fold, resulting in disruption of its intrinsic localization. We monitored the abundance of two lipoproteins found in the dataset, MltC and LolB, in bacterial membranes after incubation of purified VgrG2b$_{C\text{-}ter}$ or VgrG2b$_{C\text{-}ter}$ (E936A) with bacterial lysates. The presence of VgrG2b$_{C\text{-}ter}$ elicits a reduction in membrane-associated MltC or LolB in a manner dependent on its catalytic activity (Figures 7A and 7B). We examined the distribution of RcsF, an outer membrane lipoprotein that was identified below the significance cutoff in our pull-down experiments (Table S3), and found that its levels were also diminished in the membrane fraction in the presence of the toxin (Figure 7C). The abundance of the periplasmic protein TEM-1 was unaltered in this assay (Figure 7D), suggesting that VgrG2b$_{C\text{-}ter}$ may preferentially target lipoproteins; specifically, the toxin may cleave lipid anchors to release lipoproteins from membranes. Because this assay was conducted with non-physiological levels of both toxin and tagged lipoproteins, it is not possible to currently state whether MltC, LolB, or RcsF is a physiological target of VgrG2b when delivered by the T6SS. In all, our data indicate that the VgrG2b$_{C\text{-}ter}$ metallopeptidase represents a hitherto-undescribed family of antibacterial T6SS effectors that appear to dysregulate the cell division process.

DISCUSSION

Previous work has proposed that VgrG2b is an anti-host effector that subverts the cytoskeleton (Sana et al., 2015). VgrG2b interacts with members of the γTuRC, whereas VgrG2a, sharing 99.5% identity with VgrG2b across its spike and DUF2345 domains, does not, implying that the interaction occurs through the C-terminal extension of VgrG2b, encompassing the predicted metallopeptidase domain. The precise function of this domain was not explored, so it is unclear whether its catalytic activity is required for invasion. In addition, although ectopic expression of *vgrG2b* within host cells enhances *P. aeruginosa* uptake, it is possible that other bacterial factors are involved in the mechanism, because the internalization of neither inert particles nor other bacteria was shown. For example, other studies have found that *P. aeruginosa* can be internalized by non-phagocytic cells in a process that requires the H2-T6SS phospholipase effectors PldA and PldB (Jiang et al., 2014). Interestingly, PldA and PldB also display antibacterial activity, which necessitates a set of immunity proteins to avoid self-intoxication, and were coined trans-kingdom effectors (Jiang et al., 2014; Russell et al., 2013). In this study, we performed an in-depth characterization of VgrG2b and propose that it is also a trans-kingdom effector.

The identification of a small ORF downstream of genes encoding homologs of the VgrG2b$_{C\text{-}ter}$ metallopeptidase family is a key finding in this work. We demonstrated the impact of the VgrG2b metallopeptidase domain on bacterial survival when reaching the periplasm of prey cells in an H2-T6SS-dependent manner.

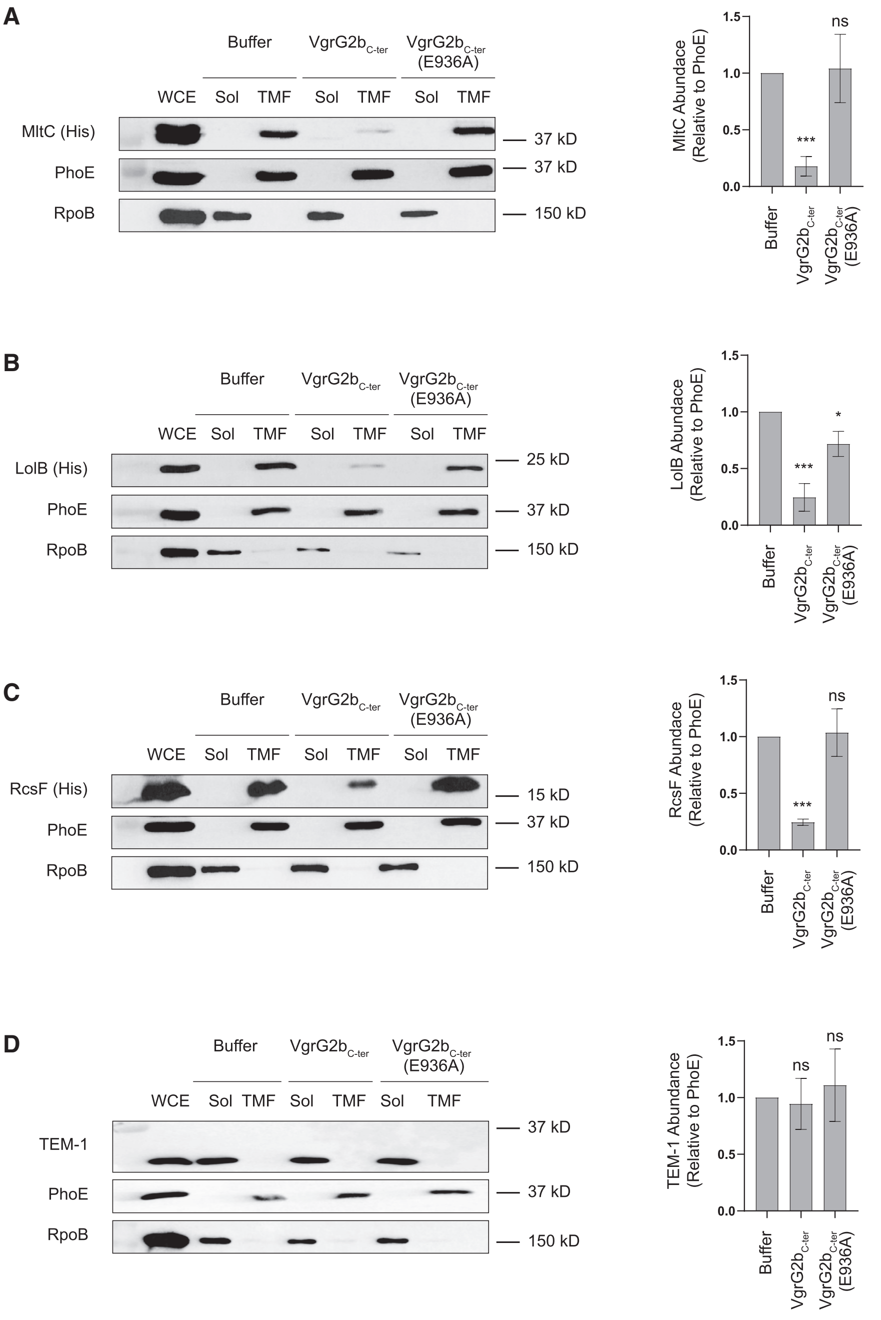

Figure 7. Incubation with VgrG2b$_{C\text{-}ter}$ Reduces the Abundance of Lipoproteins in Bacterial Cell Membranes

(A–C) Immunoblot analysis (left panels) and densitometry (right panels) of the abundance of lipoproteins MltC (A), LolB (B), and RcsF (C) in bacterial total membrane fraction (TMF) preparations in the presence of recombinant VgrG2b$_{C\text{-}ter}$, VgrG2b$_{C\text{-}ter}$ (E936A), or a buffer-only control.

(D) Abundance of the periplasmic protein TEM-1 was assessed. The outer membrane protein PhoE acts as a marker of the total membrane fraction, and RpoB serves as the soluble fraction marker. A statistically significant difference in protein abundance between incubation with buffer or recombinant protein was determined by a two-tailed Student's t test (n = 3 biological replicates; *p < 0.05, ***p < 0.001; ns, not significant). See also Figure S7.

The HEXXH motif in its C-terminal domain had led to its designation as a putative zinc-dependent metallopeptidase, and here we present the crystal structure of this domain, which confirms this classification. Our phylogenetic analyses of VgrG2b$_{C\text{-}ter}$-like proteins also find the corresponding gene within T6SS loci of diverse proteobacteria. It is frequently annotated as DUF4157, which corresponds to a small domain of approximately 80 residues encompassing the HEXXH motif. Our data show that mutations in the catalytic site abrogate the antibacterial activity, although we were unable to identify direct cleavage of a substrate by VgrG2b.

Our characterization of VgrG2b$_{C\text{-}ter}$ as an antibacterial toxin is supported by the recent description of the ACIAD0053 protein from *A. baylyi* (Ringel et al., 2017). This study showed that an *A. baylyi* strain lacking *ACIAD0053-ACIAD0054* can be eliminated by its parent, leading to the designation of the locus as encoding a type VI metallopeptidase effector-immunity pair, Tpe1-Tpi1. We have additionally demonstrated that similar to VgrG2b$_{C\text{-}ter}$, Tpe1 exerts its activity in the periplasm, whereas the cognate immunity protein Tpi1 resides in the inner membrane. The ortholog of this immunity determinant in *S. arizonae* is an outer membrane lipoprotein, but in both cases, the bulk of the immunity protein is exposed to the periplasm; accordingly, we also observe PA0261 of *P. aeruginosa* localizing to this compartment. It is therefore fitting to propose that the VgrG2b$_{C\text{-}ter}$-PA0261 family of metallopeptidase effector-immunity pairs constitutes members of the Tpe-Tpi family.

In our study, we revealed the differing ability of VgrG2b$_{C\text{-}ter}$ and the *A. baylyi* toxin Tpe1 to intoxicate *E. coli*. Work by Ringel and colleagues demonstrated Tpe1 toxicity in *A. baylyi* under standard laboratory conditions but failed to see an impact on *E. coli* (Ringel et al., 2017). We corroborate this observation under standard laboratory conditions but find that a defect in *E. coli* growth is elicited by Tpe1 in conditions of low salinity, implying that osmolarity, or salt per se, may regulate its action. Recently, a study of effector synergy within a delivered payload indicates that conditional efficacy is a common phenomenon among T6SS toxins; thus, toxin efficiency should be considered in the context of the whole T6SS-delivered payload (LaCourse et al., 2018;

Shneider et al., 2013). It may be that the biochemical action of Tpe1 is inhibited by moderate salinity, or rather its target is regulated by this condition. Alternatively, this experimental setup may simply reflect a more environmentally relevant condition and imply that VgrG2b$_{C\text{-ter}}$ is a more robust enzyme. Salinity is known to regulate periplasmic proteins such as peptidoglycan biosynthetic enzymes, presenting the possibility that these toxins may interact with different members of a family of peptidoglycan remodeling enzymes such as penicillin-binding proteins (PBPs) or lytic transglycosylases (LTases) (Möll et al., 2015; Palomino et al., 2009). Indeed, *P. aeruginosa*, *A. baylyi*, and *E. coli* possess differing quotas of these peptidoglycan hydrolases, rendering species-dependent nuances in substrate targeting an attractive hypothesis (Dik et al., 2017; Pazos et al., 2017).

We observed bulge formation at sites of septation resulting from the activity of VgrG2b$_{C\text{-ter}}$. Recently, a T6SS effector inhibiting bacterial cell division was described in *Serratia proteamaculans*, for which ADP ribosylation of the essential cell division protein FtsZ prevents Z-ring formation, causing bacterial elongation because of inhibition of septation (Ting et al., 2018). The cell division defect mediated by VgrG2b$_{C\text{-ter}}$ that we observe appears to be distinct, characterized by midcell blebbing. This is reminiscent of the inhibition of the cell wall biosynthesis machineries by β-lactam antibiotics, in which the balance of peptidoglycan growth and degradation is disrupted, causing severe morphological aberrations and cell lysis (Cho et al., 2014; Dik et al., 2019; Templin et al., 1992). Class A PBPs possess transpeptidase (TPase) and glycosyltransferase activities to incorporate new monomers into the sacculus, and these biosynthetic processes are coordinated with the degradative action of enzymes such as LTases, amidases, and carboxypeptidases (CPases), which modify the peptidoglycan to regulate its growth and permit septation of daughter cells (Egan et al., 2017). These enzymes form large complexes, whose subcellular localization often regulates their activities, and dysregulation of this process usually results in lysis. For example, the recruitment to midcell and subsequent activity of the TPase LdtD requires the concerted actions of PBP5, PBP6a, and PBP1b to facilitate septation under certain stress conditions, the latter of which is also associated with LTases MltA and MltG and is recruited to the septum by the lipoprotein LpoB (Hugonnet et al., 2016; Montón Silva et al., 2018; Morè et al., 2019; Paradis-Bleau et al., 2010; Typas et al., 2010; Vollmer et al., 1999; Yunck et al., 2016). Similarly, perturbation of the localization of EnvC and NlpD to the division site, which controls the activity of amidases required for separation of daughter cells, results in septation failure (Tsang et al., 2017; Uehara et al., 2009, 2010).

Consequently, it is thought that functional redundancy may help to insulate this crucial process from environmental insults, and correspondingly, individual deletions in LTases and many PBP genes often do not produce phenotypic changes, because the enzymes display partially overlapping functions for robust control of cell division in changing conditions (Denome et al., 1999; Heidrich et al., 2002; Pazos et al., 2017; Peters et al., 2016). Our pull-down assays identified the membrane-associated LTase MltC and the D,D-CPases PBP5 and PBP6a as possible interaction partners of VgrG2b$_{C\text{-ter}}$, yet individually they are dispensable for effector-mediated toxicity, potentially as a consequence of built-in redundancy. The precise biochemical activities and functions of these peptidoglycan biosynthetic enzymes have only started to be unraveled (Artola-Recolons et al., 2014; Dijkstra and Keck, 1996; Meiresonne et al., 2017; Peters et al., 2016), so further work is required to establish their relevance during intoxication by the T6SS.

We found that VgrG2b$_{C\text{-ter}}$ depletes several lipoproteins from membranes, implying broad target specificity by associating with the lipidated linker region of these proteins. We speculate that the activity VgrG2b$_{C\text{-ter}}$ may disrupt the localization of lipoproteins associated with cell envelope maintenance such as membrane-associated LTases, the cell wall anchoring protein Lpp, or components of the Lol and Bam machineries, thereby perturbing bacterial cell division. Targeting of lipidated substrates has been described for bacterial effectors of the type III secretion system, namely, YopT of *Yersinia enterocolitica* and IpaJ of *Shigella flexneri*. These proteases are delivered into host cells, where they cleave the isoprenyl and *N*-myristoyl anchors of small guanosine triphosphatases (GTPases), respectively, to disrupt host cell signaling and facilitate bacterial survival (Burnaevskiy et al., 2013; Shao et al., 2002). Indeed, these effectors target specific families of GTPases during infection, rather than a single protein; thus, the notion of target promiscuity for VgrG2b$_{C\text{-ter}}$ has an intriguing precedent (Burnaevskiy et al., 2015; Fueller and Schmidt, 2008).

In summary, we find that besides its described role in invasion of eukaryotic cells, the evolved VgrG2b spike protein of *P. aeruginosa* has a direct role in bacterial antagonism. Given the vast dissimilarity between host microtubule manipulation and bacteriolytic activity, reconciliation of VgrG2b metallopeptidase dual role warrants further work to shed light on the activity of this effector within eukaryotic systems. In prokaryotes, the C-terminal metallopeptidase domain elicits acute toxicity when targeted to the periplasmic space, resulting in dysregulation of bacterial morphology and cell lysis. The prevalence of this family of T6SS effector proteins suggests conservation of its target, so elucidation of its antibacterial mechanism may help progress both our knowledge of bacterial cell division and how to target this process with therapeutic compounds.

STAR★METHODS

Detailed methods are provided in the online version of this paper and include the following:

- KEY RESOURCES TABLE
- LEAD CONTACT AND MATERIALS AVAILABILITY
- EXPERIMENTAL MODEL AND SUBJECT DETAILS
- METHOD DETAILS
    - Secretion assays and immunoblot analysis
    - Protein purification
    - Crystallization experiments and structure determination
    - Differential scanning fluorimetry
    - SEC-MALLS
    - Bacterial competition assays
    - Bacterial intoxication assays
    - Subcellular fractionation of bacterial cells

- ○ Far-western dot blot analysis
- ○ Turbidometric and colorimetric analysis of VgrG2b activity
- ○ Zymography
- ○ Peptidoglycan hydrolase assay
- ○ Microscopy techniques
- ○ Mass spectrometry analysis
- ○ Analysis of lipoprotein abundance
- ○ Bioinformatic and phylogenetic analysis
- QUANTIFICATION AND STATISTICAL ANALYSIS
- DATA AND CODE AVAILABILITY

SUPPLEMENTAL INFORMATION

Supplemental Information can be found online at https://doi.org/10.1016/j.celrep.2019.08.094.

ACKNOWLEDGMENTS

The authors are grateful to Luke P. Allsopp, Sarah Wettstadt, and Silvia D'Arcangelo for their technical help; Serge Mostowy for microscopy support; Thibaut Léger and Camille Garcia at the Institut Jacques Monod proteomic facility, UMR7592, Université Paris Diderot/CNRS, for mass spectrometry analysis; Despoina A.I. Mavridou and R. Christopher D. Furniss (CMBI, Imperial College London) for *E. coli* MC1000 strains; Suzana P. Salcedo (IBCP, Lyon) for *A. baylyi* ADP1; and Gad Frankel (CMBI, Imperial College London) for *S. arizonae* genomic DNA. T.E.W. and S.A.H. are in receipt of PhD scholarships from the Wellcome Trust and Medical Research Council (MRC), respectively. E.M. is supported by MRC grant MR/N023250/1; L.M.N. is supported by MRC grant MR/N023250/1 and a Marie Curie Fellowship (PIIF-GA-2013-625318); N.P.B. is supported by a Canada Graduate Scholarship; H.C.L.Y. and W.V. are supported by Wellcome Trust grant 101824/Z/13/Z; A.H. is supported by H2020-MSCA-Global Fellowship grant 657766; R.D.H. is supported by MRC grant N000846/1; J.C.W. is supported by Canadian Institutes of Health Research (CIHR) grant PJT-156129; P.S.F. and A. Förster are supported by MRC grant MRK/K001930/1; and A. Filloux is supported by MRC grants MR/N023250/1 and MRK/K001930/1.

AUTHOR CONTRIBUTIONS

T.E.W. and A. Filloux conceived the study and wrote the manuscript; T.E.W., S.A.H., A. Förster, L.M.N., E.M., N.P.B., and H.C.L.Y. performed experiments; and A.H., R.D.H., J.C.W., W.V., and P.S.F. helped with experiment design.

DECLARATION OF INTERESTS

The authors declare no competing interests.

Received: August 7, 2018
Revised: July 2, 2019
Accepted: August 27, 2019
Published: October 1, 2019

REFERENCES

Adams, P.D., Afonine, P.V., Bunkóczi, G., Chen, V.B., Davis, I.W., Echols, N., Headd, J.J., Hung, L.-W., Kapral, G.J., Grosse-Kunstleve, R.W., et al. (2010). *PHENIX*: a comprehensive Python-based system for macromolecular structure solution. Acta Crystallogr. D Biol. Crystallogr. *66*, 213–221.

Allsopp, L.P., Wood, T.E., Howard, S.A., Maggiorelli, F., Nolan, L.M., Wettstadt, S., and Filloux, A. (2017). RsmA and AmrZ orchestrate the assembly of all three type VI secretion systems in *Pseudomonas aeruginosa*. Proc. Natl. Acad. Sci. USA *114*, 7707–7712.

Artola-Recolons, C., Lee, M., Bernardo-García, N., Blázquez, B., Hesek, D., Bartual, S.G., Mahasenan, K.V., Lastochkin, E., Pi, H., Boggess, B., et al. (2014). Structure and cell wall cleavage by modular lytic transglycosylase MltC of *Escherichia coli*. ACS Chem. Biol. *9*, 2058–2066.

Aubert, D.F., Xu, H., Yang, J., Shi, X., Gao, W., Li, L., Bisaro, F., Chen, S., Valvano, M.A., and Shao, F. (2016). A Burkholderia Type VI Effector Deamidates Rho GTPases to Activate the Pyrin Inflammasome and Trigger Inflammation. Cell Host Microbe *19*, 664–674.

Baba, T., Ara, T., Hasegawa, M., Takai, Y., Okumura, Y., Baba, M., Datsenko, K.A., Tomita, M., Wanner, B.L., and Mori, H. (2006). Construction of *Escherichia coli* K-12 in-frame, single-gene knockout mutants: the Keio collection. Mol. Syst. Biol. *2*, 0008.

Barret, M., Egan, F., Fargier, E., Morrissey, J.P., and O'Gara, F. (2011). Genomic analysis of the type VI secretion systems in *Pseudomonas* spp.: novel clusters and putative effectors uncovered. Microbiology *157*, 1726–1739.

Becher, A., and Schweizer, H.P. (2000). Integration-proficient *Pseudomonas aeruginosa* vectors for isolation of single-copy chromosomal *lacZ* and *lux* gene fusions. Biotechniques *29*, 948–950, 952.

Benz, J., Reinstein, J., and Meinhart, A. (2013). Structural Insights into the Effector-Immunity System Tae4/Tai4 from *Salmonella typhimurium*. PLoS ONE *8*, e67362.

Blanc, E., Roversi, P., Vonrhein, C., Flensburg, C., Lea, S.M., and Bricogne, G. (2004). Refinement of severely incomplete structures with maximum likelihood in BUSTER-TNT. Acta Crystallogr. D Biol. Crystallogr *60*, 2210–2221.

Brooks, T.M., Unterweger, D., Bachmann, V., Kostiuk, B., and Pukatzki, S. (2013). Lytic activity of the *Vibrio cholerae* type VI secretion toxin VgrG-3 is inhibited by the antitoxin TsaB. J. Biol. Chem. *288*, 7618–7625.

Burkinshaw, B.J., Liang, X., Wong, M., Le, A.N.H., Lam, L., and Dong, T.G. (2018). A type VI secretion system effector delivery mechanism dependent on PAAR and a chaperone-co-chaperone complex. Nat. Microbiol. *3*, 632–640.

Burnaevskiy, N., Fox, T.G., Plymire, D.A., Ertelt, J.M., Weigele, B.A., Selyunin, A.S., Way, S.S., Patrie, S.M., and Alto, N.M. (2013). Proteolytic elimination of *N*-myristoyl modifications by the *Shigella* virulence factor IpaJ. Nature *496*, 106–109.

Burnaevskiy, N., Peng, T., Reddick, L.E., Hang, H.C., and Alto, N.M. (2015). Myristoylome profiling reveals a concerted mechanism of ARF GTPase deacylation by the bacterial protease IpaJ. Mol. Cell *58*, 110–122.

Cardona, S.T., and Valvano, M.A. (2005). An expression vector containing a rhamnose-inducible promoter provides tightly regulated gene expression in *Burkholderia cenocepacia*. Plasmid *54*, 219–228.

Casadaban, M.J. (1976). Transposition and fusion of the *lac* genes to selected promoters in *Escherichia coli* using bacteriophage lambda and Mu. J. Mol. Biol. *104*, 541–555.

Casadaban, M.J., and Cohen, S.N. (1980). Analysis of gene control signals by DNA fusion and cloning in *Escherichia coli*. J. Mol. Biol. *138*, 179–207.

Chen, H., Yang, D., Han, F., Tan, J., Zhang, L., Xiao, J., Zhang, Y., and Liu, Q. (2017). The Bacterial T6SS Effector EvpP Prevents NLRP3 Inflammasome Activation by Inhibiting the Ca^{2+}-Dependent MAPK-Jnk Pathway. Cell Host Microbe *21*, 47–58.

Cho, H., Uehara, T., and Bernhardt, T.G. (2014). Beta-lactam antibiotics induce a lethal malfunctioning of the bacterial cell wall synthesis machinery. Cell *159*, 1300–1311.

Chung, H.S., Yao, Z., Goehring, N.W., Kishony, R., Beckwith, J., and Kahne, D. (2009). Rapid beta-lactam-induced lysis requires successful assembly of the cell division machinery. Proc. Natl. Acad. Sci. USA *106*, 21872–21877.

Cianfanelli, F.R., Monlezun, L., and Coulthurst, S.J. (2016). Aim, Load, Fire: The Type VI Secretion System, a Bacterial Nanoweapon. Trends Microbiol. *24*, 51–62.

Crooks, G.E., Hon, G., Chandonia, J.-M., and Brenner, S.E. (2004). WebLogo: a sequence logo generator. Genome Res. *14*, 1188–1190.

Denome, S.A., Elf, P.K., Henderson, T.A., Nelson, D.E., and Young, K.D. (1999). *Escherichia coli* mutants lacking all possible combinations of eight penicillin binding proteins: viability, characteristics, and implications for peptidoglycan synthesis. J. Bacteriol. *181*, 3981–3993.

Dijkstra, A.J., and Keck, W. (1996). Identification of new members of the lytic transglycosylase family in *Haemophilus influenzae* and *Escherichia coli*. Microb. Drug Resist. *2*, 141–145.

Dik, D.A., Marous, D.R., Fisher, J.F., and Mobashery, S. (2017). Lytic transglycosylases: concinnity in concision of the bacterial cell wall. Crit. Rev. Biochem. Mol. Biol. *52*, 503–542.

Dik, D.A., Madukoma, C.S., Tomoshige, S., Kim, C., Lastochkin, E., Boggess, W.C., Fisher, J.F., Shrout, J.D., and Mobashery, S. (2019). Slt, MltD, and MltG of Pseudomonas aeruginosa as Targets of Bulgecin A in Potentiation of β-Lactam Antibiotics. ACS Chem. Biol *14*, 296–303.

Egan, A.J.F., Cleverley, R.M., Peters, K., Lewis, R.J., and Vollmer, W. (2017). Regulation of bacterial cell wall growth. FEBS J. *284*, 851–867.

Emsley, P., Lohkamp, B., Scott, W.G., and Cowtan, K. (2010). Features and development of *Coot*. Acta Crystallogr. D Biol. Crystallogr. *66*, 486–501.

English, G., Trunk, K., Rao, V.A., Srikannathasan, V., Hunter, W.N., and Coulthurst, S.J. (2012). New secreted toxins and immunity proteins encoded within the Type VI secretion system gene cluster of *Serratia marcescens*. Mol. Microbiol. *86*, 921–936.

Filloux, A. (2011). Protein secretion systems in *Pseudomonas aeruginosa*: An essay on diversity, evolution, and function. Front. Microbiol. *2*, 155.

Flaugnatti, N., Le, T.T., Canaan, S., Aschtgen, M.S., Nguyen, V.S., Blangy, S., Kellenberger, C., Roussel, A., Cambillau, C., Cascales, E., and Journet, L. (2016). A phospholipase A1 antibacterial Type VI secretion effector interacts directly with the C-terminal domain of the VgrG spike protein for delivery. Mol. Microbiol. *99*, 1099–1118.

Foadi, J., Aller, P., Alguel, Y., Cameron, A., Axford, D., Owen, R.L., Armour, W., Waterman, D.G., Iwata, S., and Evans, G. (2013). Clustering procedures for the optimal selection of data sets from multiple crystals in macromolecular crystallography. Acta Crystallogr. D Biol. Crystallogr. *69*, 1617–1632.

Fueller, F., and Schmidt, G. (2008). The polybasic region of Rho GTPases defines the cleavage by *Yersinia enterocolitica* outer protein T (YopT). Protein Sci. *17*, 1456–1462.

Fukuhara, S., Nakane, T., Yamashita, K., Ishii, R., Ishitani, R., and Nureki, O. (2018). Crystal structure of the *Agrobacterium tumefaciens* type VI effector-immunity complex. Acta Crystallogr. F Struct. Biol. Commun. *74*, 810–816.

Glauner, B. (1988). Separation and quantification of muropeptides with high-performance liquid chromatography. Anal. Biochem. *172*, 451–464.

Hachani, A., Lossi, N.S., Hamilton, A., Jones, C., Bleves, S., Albesa-Jové, D., and Filloux, A. (2011). Type VI secretion system in *Pseudomonas aeruginosa*: secretion and multimerization of VgrG proteins. J. Biol. Chem. *286*, 12317–12327.

Hachani, A., Allsopp, L.P., Oduko, Y., and Filloux, A. (2014). The VgrG proteins are "à la carte" delivery systems for bacterial type VI effectors. J. Biol. Chem. *289*, 17872–17884.

Hachani, A., Wood, T.E., and Filloux, A. (2016). Type VI secretion and anti-host effectors. Curr. Opin. Microbiol. *29*, 81–93.

Heidrich, C., Ursinus, A., Berger, J., Schwarz, H., and Höltje, J.-V. (2002). Effects of multiple deletions of murein hydrolases on viability, septum cleavage, and sensitivity to large toxic molecules in *Escherichia coli*. J. Bacteriol. *184*, 6093–6099.

Herrero, M., de Lorenzo, V., and Timmis, K.N. (1990). Transposon vectors containing non-antibiotic resistance selection markers for cloning and stable chromosomal insertion of foreign genes in gram-negative bacteria. J. Bacteriol. *172*, 6557–6567.

Ho, B.T., Dong, T.G., and Mekalanos, J.J. (2014). A view to a kill: the bacterial type VI secretion system. Cell Host Microbe *15*, 9–21.

Holm, L., and Laakso, L.M. (2016). Dali server update. Nucleic Acids Res. *44* (*W1*), W351–W355.

Hood, R.D., Singh, P., Hsu, F., Güvener, T., Carl, M.A., Trinidad, R.R.S., Silverman, J.M., Ohlson, B.B., Hicks, K.G., Plemel, R.L., et al. (2010). A type VI secretion system of *Pseudomonas aeruginosa* targets a toxin to bacteria. Cell Host Microbe *7*, 25–37.

Hugonnet, J.E., Mengin-Lecreulx, D., Monton, A., den Blaauwen, T., Carbonnelle, E., Veckerlé, C., Brun, Y.V., van Nieuwenhze, M., Bouchier, C., Tu, K., et al. (2016). Factors essential for L,D-transpeptidase-mediated peptidoglycan cross-linking and β-lactam resistance in *Escherichia coli*. Elife *5*, e19469.

Jiang, F., Waterfield, N.R., Yang, J., Yang, G., and Jin, Q. (2014). A *Pseudomonas aeruginosa* type VI secretion phospholipase D effector targets both prokaryotic and eukaryotic cells. Cell Host Microbe *15*, 600–610.

Jiang, F., Wang, X., Wang, B., Chen, L., Zhao, Z., Waterfield, N.R., Yang, G., and Jin, Q. (2016). The *Pseudomonas aeruginosa* Type VI Secretion PGAP1-like Effector Induces Host Autophagy by Activating Endoplasmic Reticulum Stress. Cell Rep. *16*, 1502–1509.

Jiang, N., Tang, L., Xie, R., Li, Z., Burkinshaw, B., Liang, X., Sosa, D., Aravind, L., Dong, T., Zhang, D., and Zheng, J. (2019). Retraction Note: Vibrio parahaemolyticus RhsP represents a widespread group of pro-effectors for type VI secretion systems. Nat. Commun. *10*, 1277.

Jones, C., Hachani, A., Manoli, E., and Filloux, A. (2014). An *rhs* gene linked to the second type VI secretion cluster is a feature of the *Pseudomonas aeruginosa* strain PA14. J. Bacteriol. *196*, 800–810.

Kabsch, W. (2010). XDS. Acta Crystallogr. D Biol. Crystallogr. *66*, 125–132.

Kaniga, K., Delor, I., and Cornelis, G.R. (1991). A wide-host-range suicide vector for improving reverse genetics in gram-negative bacteria: inactivation of the blaA gene of *Yersinia enterocolitica*. Gene *109*, 137–141.

Katoh, K., Rozewicki, J., and Yamada, K.D. (2017). MAFFT online service: multiple sequence alignment, interactive sequence choice and visualization. Brief. Bioinform. Published online September 6, 2017. https://doi.org/10.1093/bib/bbx108.

Kemmer, G., and Keller, S. (2010). Nonlinear least-squares data fitting in Excel spreadsheets. Nat. Protoc. *5*, 267–281.

Kovach, M.E., Elzer, P.H., Hill, D.S., Robertson, G.T., Farris, M.A., Roop, R.M., 2nd, and Peterson, K.M. (1995). Four new derivatives of the broad-host-range cloning vector pBBR1MCS, carrying different antibiotic-resistance cassettes. Gene *166*, 175–176.

Krogh, A., Larsson, B., von Heijne, G., and Sonnhammer, E.L.L. (2001). Predicting transmembrane protein topology with a hidden Markov model: application to complete genomes. J. Mol. Biol. *305*, 567–580.

Kumar, S., Stecher, G., and Tamura, K. (2016). MEGA7: Molecular Evolutionary Genetics Analysis Version 7.0 for Bigger Datasets. Mol. Biol. Evol. *33*, 1870–1874.

LaCourse, K.D., Peterson, S.B., Kulasekara, H.D., Radey, M.C., Kim, J., and Mougous, J.D. (2018). Conditional toxicity and synergy drive diversity among antibacterial effectors. Nat. Microbiol. *3*, 440–446.

Lee, D.G., Urbach, J.M., Wu, G., Liberati, N.T., Feinbaum, R.L., Miyata, S., Diggins, L.T., He, J., Saucier, M., Déziel, E., et al. (2006). Genomic analysis reveals that *Pseudomonas aeruginosa* virulence is combinatorial. Genome Biol. *7*, R90.

Leiman, P.G., Basler, M., Ramagopal, U.A., Bonanno, J.B., Sauder, J.M., Pukatzki, S., Burley, S.K., Almo, S.C., and Mekalanos, J.J. (2009). Type VI secretion apparatus and phage tail-associated protein complexes share a common evolutionary origin. Proc. Natl. Acad. Sci. USA *106*, 4154–4159.

Li, J., Yao, Y.F., Xu, H.H., Hao, L., Deng, Z., Rajakumar, K., and Ou, H.Y. (2015). SecReT6: a web-based resource for type VI secretion systems found in bacteria. Environ. Microbiol *17*, 2196–2202.

Lin, J., Zhang, W., Cheng, J., Yang, X., Zhu, K., Wang, Y., Wei, G., Qian, P.-Y., Luo, Z.-Q., and Shen, X. (2017). A *Pseudomonas* T6SS effector recruits PQS-containing outer membrane vesicles for iron acquisition. Nat. Commun. *8*, 14888.

Lossi, N.S., Dajani, R., Freemont, P., and Filloux, A. (2011). Structure-function analysis of HsiF, a gp25-like component of the type VI secretion system, in *Pseudomonas aeruginosa*. Microbiology *157*, 3292–3305.

MacIntyre, D.L., Miyata, S.T., Kitaoka, M., and Pukatzki, S. (2010). The *Vibrio cholerae* type VI secretion system displays antimicrobial properties. Proc. Natl. Acad. Sci. USA *107*, 19520–19524.

Mariano, G., Monlezun, L., and Coulthurst, S.J. (2018). Dual Role for DsbA in Attacking and Targeted Bacterial Cells during Type VI Secretion System-Mediated Competition. Cell Rep. *22*, 774–785.

Mavridou, D.A.I., Ferguson, S.J., and Stevens, J.M. (2012). The interplay between the disulfide bond formation pathway and cytochrome *c* maturation in *Escherichia coli*. FEBS Lett. *586*, 1702–1707.

McCarthy, R.R., Mazon-Moya, M.J., Moscoso, J.A., Hao, Y., Lam, J.S., Bordi, C., Mostowy, S., and Filloux, A. (2017). Cyclic-di-GMP regulates lipopolysaccharide modification and contributes to *Pseudomonas aeruginosa* immune evasion. Nat. Microbiol. *2*, 17027.

McCoy, A.J., Grosse-Kunstleve, R.W., Adams, P.D., Winn, M.D., Storoni, L.C., and Read, R.J. (2007). *Phaser* crystallographic software. J. Appl. Cryst. *40*, 658–674.

Meberg, B.M., Sailer, F.C., Nelson, D.E., and Young, K.D. (2001). Reconstruction of *Escherichia coli mrcA* (PBP 1a) mutants lacking multiple combinations of penicillin binding proteins. J. Bacteriol. *183*, 6148–6149.

Meiresonne, N.Y., van der Ploeg, R., Hink, M.A., and den Blaauwen, T. (2017). Activity-Related Conformational Changes in d,d-Carboxypeptidases Revealed by *In Vivo* Periplasmic Förster Resonance Energy Transfer Assay in *Escherichia coli*. MBio *8*, 1–18.

Miller, V.L., and Mekalanos, J.J. (1988). A novel suicide vector and its use in construction of insertion mutations: osmoregulation of outer membrane proteins and virulence determinants in *Vibrio cholerae* requires *toxR*. J. Bacteriol. *170*, 2575–2583.

Möll, A., Dörr, T., Alvarez, L., Davis, B.M., Cava, F., and Waldor, M.K. (2015). A D, D-carboxypeptidase is required for *Vibrio cholerae* halotolerance. Environ. Microbiol. *17*, 527–540.

Montón Silva, A., Otten, C., Biboy, J., Breukink, E., VanNieuwenhze, M., Vollmer, W., and den Blaauwen, T. (2018). The Fluorescent D-Amino Acid NADA as a Tool to Study the Conditional Activity of Transpeptidases in *Escherichia coli*. Front. Microbiol. *9*, 2101.

Morè, N., Martorana, A.M., Biboy, J., Otten, C., Winkle, M., Serrano, C.K.G., Montón Silva, A., Atkinson, L., Yau, H., Breukink, E., et al. (2019). Peptidoglycan Remodeling Enables *Escherichia coli* To Survive Severe Outer Membrane Assembly Defect. MBio *10*, 16–18.

Mougous, J.D., Cuff, M.E., Raunser, S., Shen, A., Zhou, M., Gifford, C.A., Goodman, A.L., Joachimiak, G., Ordoñez, C.L., Lory, S., et al. (2006). A virulence locus of *Pseudomonas aeruginosa* encodes a protein secretion apparatus. Science *312*, 1526–1530.

Murshudov, G.N., Skubák, P., Lebedev, A.A., Pannu, N.S., Steiner, R.A., Nicholls, R.A., Winn, M.D., Long, F., and Vagin, A.A. (2011). *REFMAC5* for the refinement of macromolecular crystal structures. Acta Crystallogr. D Biol. Crystallogr. *67*, 355–367.

Narita, S.I., and Tokuda, H. (2017). Bacterial lipoproteins; biogenesis, sorting and quality control. Biochim. Biophys. Acta Mol. Cell. Biol. Lipids *1862*, 1414–1423.

Nielsen, H. (2017). Predicting Secretory Proteins with SignalP. Methods Mol. Biol. *1611*, 59–73.

Palmer, T., and Berks, B.C. (2012). The twin-arginine translocation (Tat) protein export pathway. Nat. Rev. Microbiol. *10*, 483–496.

Palomino, M.M., Sanchez-Rivas, C., and Ruzal, S.M. (2009). High salt stress in *Bacillus subtilis*: involvement of PBP4* as a peptidoglycan hydrolase. Res. Microbiol. *160*, 117–124.

Paradis-Bleau, C., Markovski, M., Uehara, T., Lupoli, T.J., Walker, S., Kahne, D.E., and Bernhardt, T.G. (2010). Lipoprotein cofactors located in the outer membrane activate bacterial cell wall polymerases. Cell *143*, 1110–1120.

Pazos, M., Peters, K., and Vollmer, W. (2017). Robust peptidoglycan growth by dynamic and variable multi-protein complexes. Curr. Opin. Microbiol. *36*, 55–61.

Peters, K., Kannan, S., Rao, V.A., Biboy, J., Vollmer, D., Erickson, S.W., Lewis, R.J., Young, K.D., and Vollmer, W. (2016). The redundancy of peptidoglycan carboxypeptidases ensures robust cell shape maintenance in *Escherichia coli*. MBio *7*, 1–14.

Pukatzki, S., Ma, A.T., Revel, A.T., Sturtevant, D., and Mekalanos, J.J. (2007). Type VI secretion system translocates a phage tail spike-like protein into target cells where it cross-links actin. Proc. Natl. Acad. Sci. USA *104*, 15508–15513.

Rawlings, N.D., Barrett, A.J., Thomas, P.D., Huang, X., Bateman, A., and Finn, R.D. (2018). The MEROPS database of proteolytic enzymes, their substrates and inhibitors in 2017 and a comparison with peptidases in the PANTHER database. Nucleic Acids Res. *46* (*D1*), D624–D632.

Renault, M.G., Zamarreno Beas, J., Douzi, B., Chabalier, M., Zoued, A., Brunet, Y.R., Cambillau, C., Journet, L., and Cascales, E. (2018). The gp27-like Hub of VgrG Serves as Adaptor to Promote Hcp Tube Assembly. J. Mol. Biol. *430* (*18 Pt B*), 3143–3156.

Rietsch, A., Vallet-Gely, I., Dove, S.L., and Mekalanos, J.J. (2005). ExsE, a secreted regulator of type III secretion genes in *Pseudomonas aeruginosa*. Proc. Natl. Acad. Sci. USA *102*, 8006–8011.

Ringel, P.D., Hu, D., and Basler, M. (2017). The Role of Type VI Secretion System Effectors in Target Cell Lysis and Subsequent Horizontal Gene Transfer. Cell Rep. *21*, 3927–3940.

Ruhe, Z.C., Nguyen, J.Y., Xiong, J., Koskiniemi, S., Beck, C.M., Perkins, B.R., Low, D.A., and Hayes, C.S. (2017). CdiA Effectors Use Modular Receptor-Binding Domains To Recognize Target Bacteria. MBio *8*, 1–16.

Russell, A.B., Hood, R.D., Bui, N.K., LeRoux, M., Vollmer, W., and Mougous, J.D. (2011). Type VI secretion delivers bacteriolytic effectors to target cells. Nature *475*, 343–347.

Russell, A.B., Singh, P., Brittnacher, M., Bui, N.K., Hood, R.D., Carl, M.A., Agnello, D.M., Schwarz, S., Goodlett, D.R., Vollmer, W., and Mougous, J.D. (2012). A widespread bacterial type VI secretion effector superfamily identified using a heuristic approach. Cell Host Microbe *11*, 538–549.

Russell, A.B., LeRoux, M., Hathazi, K., Agnello, D.M., Ishikawa, T., Wiggins, P.A., Wai, S.N., and Mougous, J.D. (2013). Diverse type VI secretion phospholipases are functionally plastic antibacterial effectors. Nature *496*, 508–512.

Russell, A.B., Peterson, S.B., and Mougous, J.D. (2014). Type VI secretion system effectors: poisons with a purpose. Nat. Rev. Microbiol. *12*, 137–148.

Salih, O., He, S., Planamente, S., Stach, L., MacDonald, J.T., Manoli, E., Scheres, S.H.W., Filloux, A., and Freemont, P.S. (2018). Atomic Structure of Type VI Contractile Sheath from *Pseudomonas aeruginosa*. Structure *26*, 329–336.

Sana, T.G., Baumann, C., Merdes, A., Soscia, C., Rattei, T., Hachani, A., Jones, C., Bennett, K.L., Filloux, A., Superti-Furga, G., et al. (2015). Internalization of *Pseudomonas aeruginosa* Strain PAO1 into Epithelial Cells Is Promoted by Interaction of a T6SS Effector with the Microtubule Network. MBio *6*, e00712.

Santin, Y.G., and Cascales, E. (2017). Measure of Peptidoglycan Hydrolase Activity. Methods Mol. Biol. *1615*, 151–158.

Santini, C.-L., Bernadac, A., Zhang, M., Chanal, A., Ize, B., Blanco, C., and Wu, L.-F. (2001). Translocation of jellyfish green fluorescent protein via the Tat system of *Escherichia coli* and change of its periplasmic localization in response to osmotic up-shock. J. Biol. Chem. *276*, 8159–8164.

Schindelin, J., Arganda-Carreras, I., Frise, E., Kaynig, V., Longair, M., Pietzsch, T., Preibisch, S., Rueden, C., Saalfeld, S., Schmid, B., et al. (2012). Fiji: an open-source platform for biological-image analysis. Nat. Methods *9*, 676–682.

Shang, G., Liu, X., Lu, D., Zhang, J., Li, N., Zhu, C., Liu, S., Yu, Q., Zhao, Y., Zhang, H., et al. (2012). Structural insight into how Pseudomonas aeruginosa peptidoglycanhydrolase Tse1 and its immunity protein Tsi1 function. Biochem. J. *448*, 201–211.

Shao, F., Merritt, P.M., Bao, Z., Innes, R.W., and Dixon, J.E. (2002). A *Yersinia* effector and a *Pseudomonas* avirulence protein define a family of cysteine proteases functioning in bacterial pathogenesis. Cell *109*, 575–588.

Sheldrick, G.M. (2010). Experimental phasing with *SHELXC/D/E*: combining chain tracing with density modification. Acta Crystallogr. D Biol. Crystallogr. *66*, 479–485.

Shneider, M.M., Buth, S.A., Ho, B.T., Basler, M., Mekalanos, J.J., and Leiman, P.G. (2013). PAAR-repeat proteins sharpen and diversify the type VI secretion system spike. Nature *500*, 350–353.

Si, M., Wang, Y., Zhang, B., Zhao, C., Kang, Y., Bai, H., Wei, D., Zhu, L., Zhang, L., Dong, T.G., and Shen, X. (2017a). The Type VI Secretion System Engages a Redox-Regulated Dual-Functional Heme Transporter for Zinc Acquisition. Cell Rep. *20*, 949–959.

Si, M., Zhao, C., Burkinshaw, B., Zhang, B., Wei, D., Wang, Y., Dong, T.G., and Shen, X. (2017b). Manganese scavenging and oxidative stress response mediated by type VI secretion system in *Burkholderia thailandensis*. Proc. Natl. Acad. Sci. USA *114*, E2233–E2242.

Silverman, J.M., Agnello, D.M., Zheng, H., Andrews, B.T., Li, M., Catalano, C.E., Gonen, T., and Mougous, J.D. (2013). Haemolysin coregulated protein is an exported receptor and chaperone of type VI secretion substrates. Mol. Cell *51*, 584–593.

Templin, M.F., Edwards, D.H., and Höltje, J.-V. (1992). A murein hydrolase is the specific target of bulgecin in *Escherichia coli*. J. Biol. Chem. *267*, 20039–20043.

Ting, S.-Y., Bosch, D.E., Mangiameli, S.M., Radey, M.C., Huang, S., Park, Y.-J., Kelly, K.A., Filip, S.K., Goo, Y.A., Eng, J.K., et al. (2018). Bifunctional Immunity Proteins Protect Bacteria against FtsZ-Targeting ADP-Ribosylating Toxins. Cell *175*, 1380–1392.e14.

Tsang, M.-J., Yakhnina, A.A., and Bernhardt, T.G. (2017). NlpD links cell wall remodeling and outer membrane invagination during cytokinesis in *Escherichia coli*. PLoS Genet. *13*, e1006888.

Typas, A., Banzhaf, M., van den Berg van Saparoea, B., Verheul, J., Biboy, J., Nichols, R.J., Zietek, M., Beilharz, K., Kannenberg, K., von Rechenberg, M., et al. (2010). Regulation of peptidoglycan synthesis by outer-membrane proteins. Cell *143*, 1097–1109.

Uehara, T., Dinh, T., and Bernhardt, T.G. (2009). LytM-domain factors are required for daughter cell separation and rapid ampicillin-induced lysis in *Escherichia coli*. J. Bacteriol. *191*, 5094–5107.

Uehara, T., Parzych, K.R., Dinh, T., and Bernhardt, T.G. (2010). Daughter cell separation is controlled by cytokinetic ring-activated cell wall hydrolysis. EMBO J. *29*, 1412–1422.

Vasseur, P., Vallet-Gely, I., Soscia, C., Genin, S., and Filloux, A. (2005). The *pel* genes of the *Pseudomonas aeruginosa* PAK strain are involved at early and late stages of biofilm formation. Microbiology *151*, 985–997.

Vermelho, A.B., Meirelles, M.N.L., Lopes, A., Petinate, S.D.G., Chaia, A.A., and Branquinha, M.H. (1996). Detection of extracellular proteases from microorganisms on agar plates. Mem. Inst. Oswaldo Cruz *91*, 755–760.

Vollmer, W., von Rechenberg, M., and Höltje, J.-V. (1999). Demonstration of molecular interactions between the murein polymerase PBP1B, the lytic transglycosylase MltA, and the scaffolding protein MipA of *Escherichia coli*. J. Biol. Chem. *274*, 6726–6734.

Wang, Y.D., Zhao, S., and Hill, C.W. (1998). Rhs elements comprise three subfamilies which diverged prior to acquisition by *Escherichia coli*. J. Bacteriol. *180*, 4102–4110.

Wang, J., Brackmann, M., Castaño-Díez, D., Kudryashev, M., Goldie, K.N., Maier, T., Stahlberg, H., and Basler, M. (2017). Cryo-EM structure of the extended type VI secretion system sheath-tube complex. Nat. Microbiol. *2*, 1507–1512.

Wettstadt, S., Wood, T.E., Fecht, S., and Filloux, A. (2019). Delivery of the *Pseudomonas aeruginosa* Phospholipase Effectors PldA and PldB in a VgrG- and H2-T6SS-Dependent Manner. Front. Microbiol. *10*, 1718.

Whitney, J.C., Quentin, D., Sawai, S., LeRoux, M., Harding, B.N., Ledvina, H.E., Tran, B.Q., Robinson, H., Goo, Y.A., Goodlett, D.R., et al. (2015). An interbacterial NAD(P)($^{+}$) glycohydrolase toxin requires elongation factor Tu for delivery to target cells. Cell *163*, 607–619.

Wood, T.E., Howard, S.A., Wettstadt, S., and Filloux, A. (2019). PAAR proteins act as the ‘sorting hat’ of the type VI secretion system. Microbiology, Published online August 5, 2019. https://doi.org/10.1099/mic.0.000842. https://www.ncbi.nlm.nih.gov/entrez/query.fcgi?cmd=Retrieve&db=PubMed&list_uids=31380737&dopt=Abstract.

Yamaguchi, K., Yu, F., and Inouye, M. (1988). A single amino acid determinant of the membrane localization of lipoproteins in *E. coli*. Cell *53*, 423–432.

Yao, Z., Kahne, D., and Kishony, R. (2012). Distinct single-cell morphological dynamics under beta-lactam antibiotics. Mol. Cell *48*, 705–712.

Yunck, R., Cho, H., and Bernhardt, T.G. (2016). Identification of MltG as a potential terminase for peptidoglycan polymerization in bacteria. Mol. Microbiol. *99*, 700–718.

STAR★METHODS

KEY RESOURCES TABLE

REAGENT or RESOURCE	SOURCE	IDENTIFIER
Antibodies		
Mouse monoclonal anti-RpoB	Biolegend	Cat#663006; RRID: AB_2565555
Rabbit polyclonal anti-Hcp2	Jones et al., 2014	N/A
Rabbit polyclonal anti-VgrG2b	Sana et al., 2015	N/A
Rabbit polyclonal anti-LasB	Gift from Romé Voulhoux	N/A
Rabbit polyclonal anti-VgrG4b	Allsopp et al., 2017	N/A
Mouse monoclonal anti-Myc	Millipore	Cat#05-724; RRID: AB_309938
Mouse monoclonal anti-HA	Biolegend	Cat#MMS-101P; RRID: AB_2314672
Mouse monoclonal anti-TEM-1	QED Bioscience	Cat#15720; RRID: AB_129940
Mouse monoclonal anti-His	Sigma	Cat#H1029; RRID: AB_260015
Rabbit polyclonal anti-DsbA	Gift from Despoina Mavridou	N/A
Rabbit polyclonal anti-SecA	Gift from Jan Tommassen	N/A
Rabbit polyclonal anti-PhoE	Gift from Jan Tommassen	N/A
Bacterial and Virus Strains		
Pseudomonas aeruginosa PAO1	Dieter Haas Laboratory (Filloux lab wild type)	N/A
P. aeruginosa PAO1Δ*rsmA*	This paper	N/A
P. aeruginosa PAO1Δ*rsmA*Δ*tssE2*	Wettstadt et al., 2019	N/A
P. aeruginosa PAO1Δ*rsmA*Δ*vgrG2b*	Wood et al., 2019	N/A
P. aeruginosa PAO1Δ*rsmA*Δ*vgrG2bPA0261*	This paper	N/A
P. aeruginosa PA14	Lee et al., 2006 (Whitney lab wild type)	N/A
P. aeruginosa PA14Δ*rsmA*Δ*amrZ*	This paper	N/A
P. aeruginosa PA14Δ*rsmA*Δ*amrZ* Δ*PA14_03210* (*PA0261*^{PA14})	This paper	N/A
P. aeruginosa PA14Δ*rsmA*Δ*amrZ*Δ*PA14_03210-20* (*vgrG2bPA0261*^{PA14})	This paper	N/A
P. aeruginosa PA14Δ*rsmA*Δ*amrZ*Δ*clpV2*Δ*PA14_03210*	This paper	N/A
Escherichia coli DH5α	Lab Stock	N/A
E. coli BL21 (λDE3)	Lab Stock	N/A
E. coli B834 (λDE3)	Lab Stock	N/A
E. coli CC118λ*pir*	Herrero et al., 1990	N/A
E. coli Sm10λ*pir*	Miller and Mekalanos, 1988	N/A
E. coli 1047 pRK2013	Hachani et al., 2014	N/A
E. coli XL1-Blue	Agilent	Cat#200249
E. coli MC4100	Casadaban, 1976	N/A
E. coli MC1061	Casadaban and Cohen, 1980	N/A
E. coli CS703/1	Meberg et al., 2001	N/A
E. coli MC1000	Mavridou et al., 2012	N/A
E. coli MC1000 *dsbA::Km*	Mavridou et al., 2012	N/A
E. coli BW25113	Keio collection	N/A
E. coli BW25113Δ*mltC*	Keio collection	N/A
E. coli BW25113Δ*dacA*	Keio collection	N/A
E. coli BW25113Δ*dacC*	Keio collection	N/A
Acinetobacter baylyi ADP1	Suzana Salcedo	N/A

(*Continued on next page*)

Continued

REAGENT or RESOURCE	SOURCE	IDENTIFIER
Chemicals, Peptides, and Recombinant Proteins		
Arabinose	Sigma Aldrich	Cat#A3256
Cellosyl	(Gift from Hoechst, Germany)	N/A
FM1-43	ThermoFisher	Cat#T3163
HA Peptide	Sigma Aldrich	Cat#11666975001
IPTG	Melford	Cat#MB1008
Lysozyme	Roche	Cat#10837059001
Proteinase K	QIAGEN	Cat#19131
Thrombin Protease	Sigma Aldrich	Cat#GE27-0846-01
VgrG4b$_{C\text{-}ter}$	Allsopp et al., 2017	N/A
X-gal	Melford	Cat#M1001
Critical Commercial Assays		
SuperSignal West Pico PLUS Chemiluminescent Substrate	ThermoFisher	Cat#34580
Deposited Data		
VgrG2b$_{C\text{-}ter}$ crystal structure	This paper	PDB: 6H56
VgrG2b$_{C\text{-}ter}$ crystal diffraction data	This paper	Zenodo: 10.5281/zenodo.3246345
Oligonucleotides		
See Table S4 for full list of primers		N/A
Recombinant DNA		
pBBR1-MCS-4	Kovach et al., 1995	N/A
pBBR1-MCS-4-*tssE2-his*$_6$	This paper	N/A
pET28a	Novagen	Cat#69864
pET28a-*vgrG2b*$_{C\text{-}ter}$	This paper	N/A
pET28a-*vgrG2b*$_{C\text{-}ter}$ (E936A)	This paper	N/A
pET28a-*mltC*	This paper	N/A
pET28a-*lolB*	This paper	N/A
pET28a-*rcsF*	This paper	N/A
pTat	This paper	N/A
pTat-*vgrG2b*$_{C\text{-}ter}$-*myc*	This paper	N/A
pTat-*vgrG2b*$_{C\text{-}ter}$ (E936A)-*myc*	This paper	N/A
pET22b	Novagen	Cat#69744
pET22b-*PA0261-HA*	This paper	N/A
pET22b-*tli4-HA*	This paper	N/A
pBBR1-MCS-5	Kovach et al., 1995	N/A
pBBR1-MCS-5-*PA0261-HA*	This paper	N/A
pBBR1-MCS-5-*PA0261*(no SP)-*HA*	This paper	N/A
pBBR1-MCS-5-*tli3-HA*	Wood et al., 2019	N/A
pTat-*ACIAD0053-FLAG*	This paper	N/A
pTat-*ACIAD0053*-FLAG*	This paper	N/A
pET22b-*SARI_02726-HA*	This paper	N/A
pET22b-*ACIAD0054-HA*	This paper	N/A
pET22b-*vgrG2a*$_{C\text{-}ter}$-*strepII*	This paper	N/A
pET22b-*vgrG2b*$_{C\text{-}ter}$ (E936A)	This paper	N/A
pET22b-*vgrG2b*$_{C\text{-}ter}$ (E936A)-*strepII*	This paper	N/A
pSCrhaB2-CV	Cardona and Valvano, 2005	N/A
pSCrhaB2-CV-peri-*vgrG2b*(847-1019)^{PA14}	This paper	N/A
pSCrhaB2-CV-peri-*vgrG2b*(847-1019) ^{PA14} (H935A)	This paper	N/A
pSCrhaB2-CV-peri-*vgrG2b*(847-1019) ^{PA14} (E936A)	This paper	N/A

(*Continued on next page*)

Continued

REAGENT or RESOURCE	SOURCE	IDENTIFIER
pSCrhaB2-CV-peri-*vgrG2b*(847-1019) ^{PA14} (H939A)	This paper	N/A
pPSV39-CV	Silverman et al., 2013	N/A
pPSV39-CV-*PA14_03210* (*PA0261*^{PA14})	This paper	N/A
Mini-CTX-*lacZ*	Becher and Schweizer, 2000	N/A
pEXG2	Rietsch et al., 2005	N/A
pEXG2-Δ*PA14_52570* (*rsmA*^{PA14})	This paper	N/A
pEXG2-Δ*PA14_20290* (*amrZ*^{PA14})	This paper	N/A
pEXG2-Δ*PA14_03210* (*PA0261*^{PA14})	This paper	N/A
pEXG2-Δ*PA14_03210-20* (*vgrG2bPA0261*^{PA14})	This paper	N/A
pEXG2-Δ*PA14_42980* (*clpV2*^{PA14})	This paper	N/A
pKNG101	Kaniga et al., 1991	N/A
pKNG101-Δ*rsmA*	Allsopp et al., 2017	N/A
pKNG101-Δ*tssE2*	Wettstadt et al., 2019	N/A
pKNG101-Δ*vgrG2b*	Wood et al., 2019	N/A
pKNG101-Δ*vgrG2bPA0261*	This paper	N/A
Software and Algorithms		
Prism 8.0	Graphpad	https://www.graphpad.com/scientific-software/prism/
FIJI	Schindelin et al., 2012	https://fiji.sc/
PyMol	Schrödinger	https://pymol.org/2/
COOT	Emsley et al., 2010	https://www2.mrc-lmb.cam.ac.uk/personal/pemsley/coot/

LEAD CONTACT AND MATERIALS AVAILABILITY

Further information and requests for resources and reagents should be directed to and will be fulfilled by the Lead Contact, Alain Filloux (a.filloux@imperial.ac.uk). All reagents generated in this work shall be shared upon request without restrictions.

EXPERIMENTAL MODEL AND SUBJECT DETAILS

Bacterial strains were grown at 37°C in lysogeny broth (LB) with agitation unless stated otherwise. All cultures were supplemented with antibiotics and other supplements where necessary. The following antibiotic concentrations were employed for *E. coli*: 50 μg/ml kanamycin, 50 μg/ml ampicillin, 50 μg/ml streptomycin, 34 μg/ml chloramphenicol, 15 μg/ml tetracycline, 200 μg/ml trimethoprim and 15 μg/ml gentamicin. For *P. aeruginosa*, 50 μg/ml gentamicin, 2 mg/ml streptomycin, 50 μg/ml tetracycline and 100 μg/ml carbenicillin were used. Deletion mutants were constructed as previously described (Vasseur et al., 2005). Briefly, splicing by overlap extension polymerase chain reaction (PCR) was used to generate DNA fragments of regions of the *P. aeruginosa* genome with in-frame gene deletions introduced, which were cloned into the suicide vector pKNG101. After mobilisation into *P. aeruginosa* by three-partner conjugation from *E. coli* CC118λpir and with the 1047 pRK2013 helper strain, selection of conjugants was achieved on Vogel-Bonner medium (20 mM magnesium sulfate heptahydrate, 200 mg anhydrous citric acid, 1 g potassium phosphate dibasic and 350 mg ammonium sodium phosphate dibasic tetrahydrate) supplemented with 1.5% (w/v) agar and 2 mg/ml streptomycin. Counter-selection on solid LB medium containing 20% (w/v) sucrose at ambient temperature for 72 h lead to plasmid excision and generation double recombinants. All mutants were confirmed by PCR and sequencing.

METHOD DETAILS

Secretion assays and immunoblot analysis

P. aeruginosa secretion assays were conducted as previously described (Allsopp et al., 2017) with the following modifications. Cultures were inoculated into 25 mL tryptic soy broth (TSB) at OD_{600} 0.1 and grown for 8 h at 25°C with agitation. Supernatants were cleared of cells by four rounds of centrifugation at 4000 *g* at 4°C, successively taking the uppermost supernatant. Proteins were precipitated with 10% trichloroacetic acid supplemented with 0.03% sodium deoxycholate overnight at 4°C. Separation of protein samples by SDS-PAGE and subsequent immunoblot analysis was conducted as previously described (Hachani et al., 2011) with the exception of culture supernatants being loaded at 20x concentrated in comparison to the whole cell extracts.

Here, bacterial culture samples were normalized to an OD_{600} 1.0. Gels containing 6%, 8%, 10%, 12% or 15% polyacrylamide were used to separate protein samples by SDS-polyacrylamide gel electrophoresis (SDS-PAGE) in Tris-glycine-SDS buffer (0.3% (w/v) Tris, 1.44% (w/v) glycine, 0.01% (w/v) SDS) at 180 V, depending on the size resolution desired. Gels were prepared using Mini-PROTEAN Tetra handcast systems (BioRad) and the Precision Plus Protein Kaleidoscope Prestained Protein Standards marker (Bio-Rad) was loaded to show the migration of molecular weight standards. Samples were stained with Coomassie Brilliant Blue R250 (Sigma) or subjected to immunoblotting. For immunoblot analysis, protein samples were transferred to 0.2 μm nitrocellulose membrane (Amersham Biosciences) after incubation in transfer buffer (10% (v/v) Tris-glycine-SDS buffer, 20% (v/v) ethanol) using a TransBlot SD semi-dry transfer cell (BioRad) at 24 V and 0.18 A for 44 min. Membranes were blocked using a solution of 5% (w/v) skimmed milk powder (Sigma), 50 mM Tris-HCl pH 8.0, 150 mM NaCl and 0.1% (v/v) Tween-20 for 1 h before the addition of primary antibodies. Primary antibodies were used at 1/1000 dilutions in blocking buffer overnight at 4°C, aside from anti-RpoB and anti-PhoE, which were used at 1/5000 and 1/2000, respectively. Membranes were washed in blocking buffer lacking milk three times before the addition of horseradish peroxidase-conjugated secondary antibodies for 45 min at room temperature. Secondary antibodies were used at 1/5000 dilution. Membranes were once again washed three times before development using SuperSignal West Pico Chemiluminescent substrate (ThermoFisher) and imaging using a LAS-3000 imager (Fujifilm).

Protein purification

Purification of proteins was performed as described previously (Allsopp et al., 2017). Briefly, *E. coli* BL21 (λDE3) pET28a-*vgrG2b*$_{\text{C-ter}}$ or pET28a-*vgrG2b*$_{\text{C-ter}}$ (E936A) were grown in terrific broth at 37°C for 2 h prior to induction of gene expression by addition of 1 mM isopropyl-β-D-thiogalactopyranoside (IPTG) and growth overnight at 18°C. B834 (λDE3) was the host for selenomethionine incorporation. Cell harvesting and protein purification was done as described, but without protease inhibitors. The C-terminal hexahistidine tag was cleaved by thrombin protease (Sigma Aldrich) and dialysed into low imidazole buffer (50 mM Tris-HCl pH 8.0, 500 mM sodium chloride, 20 mM imidazole) before separation from non-cleaved protein on a Ni^{2+}-NTA column in the flow-through fractions. Proteins were concentrated in Amicon Ultra Centrifugal Filter Units (Millipore) before further purification by SEC using a Superdex S200 10/300 GL column (GE Healthcare). Purity of elution fractions was determined by Coomassie staining of proteins separated by SDS-PAGE.

Crystallization experiments and structure determination

Purified VgrG2b$_{\text{C-ter}}$ was concentrated to 12 mg/ml and centrifuged for 20 minutes at 4°C to remove dust and aggregates. The protein was crystallized by vapor diffusion at 20°C, with small crystals growing in 100 Mm bis-tris pH 6.5 and 45% polypropylene glycol P400. Their space group was $P3_1$ and they diffracted to 3.2 Å. The structure was solved by experimental phasing, whereby crystals of selenomethionylated protein were grown in 100 mM sodium citrate pH 4.7, 20 mM magnesium chloride, 50 mM sodium chloride and 29% PEG400. These crystals had space group $P42_12$ and diffracted to 3.0 Å. Data were collected at the beamlines i02 and i04 of Diamond Light Source (Didcot, UK) from crystals flash-frozen in liquid nitrogen without additional cryoprotection. Diffraction data from both experiments can be downloaded from Zenodo (https://doi.org/10.5281/zenodo.3246345). Data were processed in XDS (Kabsch, 2010). The structure was solved by single anomalous dispersion based on the selenomethionine signal, although due to limited resolution and low anomalous signal, four datasets were combined in Blend (Foadi et al., 2013) to determine the anomalous substructure in SHELX (Sheldrick, 2010). A partial model comprising secondary structure elements was built by phenix.autobuild over several rounds of rebuilding and refinement before being used for molecular replacement with the $P3_1$ dataset in Phaser (McCoy et al., 2007). The new model was rebuilt in Coot and refined in Refmac5 (Murshudov et al., 2011), phenix.refine (Adams et al., 2010) and Buster-TNT (Blanc et al., 2004) until convergence. The crystallographic statistics are summarized in Table S1. Interface analysis was performed on the Proteins, Interfaces, Structures and Assemblies (PISA) server while molecular graphics figures were prepared with PyMol (Schrödinger).

Differential scanning fluorimetry

Analysis of the thermal stability of recombinant VgrG2b$_{\text{C-ter}}$ protein in the presence and absence of zinc ions was determined using a Mx3005P qPCR instrument (Agilent). VgrG2b$_{\text{C-ter}}$ was used at 1.25 μM, in a buffer of 10 mM HEPES pH 7.4 and 100 mM NaCl supplemented with zinc acetate as indicated. The fluorescent dye SyproOrange (Sigma Aldrich) was used at a 1/1000 dilution and the thermal unfolding of VgrG2b$_{\text{C-ter}}$ was monitored between 25-98°C at a rate of 1.5K/min. Excitation occurred at 492 nm and emission fluorescence at 610 nm was measured every 40 s. Non-linear least-squares fitting (Kemmer and Keller, 2010) was used to analyze the raw data and the melting point of each sample was determined from the inflection point of the fitted curve.

SEC-MALLS

The oligomeric state of recombinant VgrG2b$_{\text{C-ter}}$ was determined using SEC-MALLS and refractometry. VgrG2b$_{\text{C-ter}}$ was loaded onto a Superdex 75 10/30 column (GE Healthcare) and separated at 20°C with a flow rate of 0.5 ml/min. MALLS detection was performed with a miniDAWN TREOS detector (Wyatt Technology) using a laser emitting at 657.3 nm. The refractive index of the solution was measured with an Optilab T-rEX detector (Wyatt Technology) with the refractive-index increment (dn/dc) set to 0.185 ml/g. Weight-averaged molar masses were calculated using ASTRA software (Wyatt Technology).

Bacterial competition assays

The *P. aeruginosa* strains used in intraspecies competitions were differentiated by integration of Mini-CTX-*lacZ* at the chromosomal *att* site of the prey strains, permitting blue/white screening on 5-bromo-4-chloro-3-indolyl-D-galactopyranoside (X-gal)-containing solid media. Bacteria were grown overnight in 5 mL LB with appropriate antibiotics before normalization to an OD_{600} 3.0 in 1 mL sterile PBS. Attacker and prey strains were mixed at a 1:1 ratio and 5 μl competition drops were spotted onto dry 3% low salt LB agar (10 g bactopeptone, 5 g yeast extract, 30 g bacteriological agar per liter). After drying, competitions were incubated at 25°C for 24 h. Both the input and output of competition spots were serially diluted in PBS and plated on LB containing 100 μg/ml X-gal for enumeration of colony forming units (CFU). The competitive index was calculated as the ratio between the input and output attacker/prey ratios. For self-intoxication assays on solid media, overnight cultures of *P. aeruginosa* strains were normalized and spotted onto nitrocellulose membrane on 3% LB agar containing gentamicin and 300 μM IPTG. CFUs were enumerated after 18 hours of growth at 25°C. For the corresponding growth curves in liquid media, overnight cultures were back-diluted 200-fold into LB broth containing gentamicin and 300 μM IPTG. Cultures were grown at 25°C with agitation in a 96-well plate, and OD_{600} readings were taken every 30 minutes for 18 hours using a Synergy 4 Microplate Reader (Biotek Instruments).

Bacterial intoxication assays

E. coli DH5α was transformed with pTat plasmids harboring variants of T6SS metallopeptidase genes to target the gene products to the periplasmic space and BL21 (λDE3) was the host when pET28a and pET22b plasmids were used. Investigation into the role of cell wall biosynthetic enzymes employed the corresponding deletion strains and the BW25113 parent strain from the Keio collection as hosts (Baba et al., 2006). The host for the peri-*vgrG2b*(847-1019) plasmids was *E. coli* XL1-Blue harboring the immunity gene under a leaky promoter, since the presence of the plasmid containing the wild-type peri-*vgrG2b*(847-1019) construct was otherwise not tolerated. Overnight cultures of the strains harboring the vectors of interest were grown in LB, normalized and serially diluted. Dilutions were spotted on LB agar containing inducer (100 μM IPTG, 0.2% arabinose or 0.3% rhamnose) or repressor (0.2% glucose). To modify the osmolality of solid media, low tonicity was achieved with low salt LB agar, while to raise it LB agar was supplemented with 500 mM sucrose.

Subcellular fractionation of bacterial cells

The localization of proteins within bacterial cells was probed by membrane fractionation and periplasmic extraction procedures. To extract the periplasm, *E. coli* cells producing the proteins of interest were normalized to OD_{600} 20 and resuspended in 200 μl spheroplast buffer (200 mM Tris-HCl pH 8.0, 500 μM EDTA, 500 mM sucrose) to which 50 μg hen egg-white lysozyme (Roche) was added and incubated at 4°C for 15 min. To this, 720 μl half-strength spheroplast buffer was added and incubated for a further 15 min to release the periplasmic contents. Centrifugation at 5000 *g* at 4°C for 5 min separated the spheroplasts from the periplasmic fraction. To assess the presence of proteins in the inner and outer membranes, *E. coli* cultures were resuspended in sonication buffer (50 mM Tris-HCl pH 8.0, 1 mM EDTA) and lysed using a Vibra-Cell sonicator (Sonics & Materials, Inc.) on ice. Centrifugation at 4000 *g* cleared the soluble fraction from the cellular debris before ultracentrifugation at 100 000 *g* for 1 h at 4°C pelleted the total membrane fraction. After washing the membranes with sonication buffer, solubilisation buffer (15 mM Tris-HCl pH 7.4, 2% sodium lauroyl sarcosinate) was added to differentially solubilise the inner and outer membrane proteins before further ultracentrifugation to pellet the insoluble outer membrane protein fraction. The membrane fractions were separated, and the pellet was washed with solubilisation buffer. All fractions were normalized prior to analysis by SDS-PAGE.

Far-western dot blot analysis

Protein-protein interactions were probed by far-western dot blotting as described previously (McCarthy et al., 2017). Briefly, 10 μg purified protein of interest or HA peptide (Sigma Aldrich) was spotted onto nitrocellulose membrane and air-dried before blocking. Lysates of *E. coli* strains producing the prey proteins with C-terminal HA tags were obtained by sonication and normalized to OD_{600} 10 in protein binding buffer (20 mM Tris-HCl pH 7.6, 100 mM sodium chloride, 10% glycerol, 0.1% Tween-20, 2% skimmed milk powder). Membranes were incubated with the lysates or protein binding buffer overnight at 4°C prior to washing three times and immunoblotting for the bait and prey proteins.

Turbidometric and colorimetric analysis of VgrG2b activity

Generic protease activity of 10 μg recombinant $VgrG2b_{C\text{-}ter}$, its inactive mutant and a proteinase K control was assessed using 1% proteinaceous substrates bovine serum albumin and gelatin in 2% bacteriological agar plates. After incubation at 37°C for 24 h, plates were stained with amido black solution (Vermelho et al., 1996) to visualize zones of proteolysis. Lysozyme activity of recombinant proteins utilized the lyophilised substrate *Micrococcus lysodeikticus* (Sigma Aldrich) as previously documented (Lossi et al., 2011). The polymyxin B-mediated permeabilisation of the bacterial outer membrane to permit access of exogenous proteins to the periplasmic space was performed as described elsewhere (Brooks et al., 2013), except *E. coli* was employed. *E. coli* DH5α was resuspended in turbidometry buffer (50 mM Tris-HCl pH 8.0, 150 mM NaCl) at OD_{600} 0.2 and incubated with 4 μg/ml polymyxin B. After addition of 10 μg purified protein, the turbidity at OD_{600} was monitored at five-minute intervals for 1 h.

Zymography

Peptidoglycan was purified from 500 mL *E. coli* MC4100 culture for zymographic analysis as described elsewhere (Brooks et al., 2013; Santin and Cascales, 2017). Briefly, 10 μg purified protein was separated by SDS-PAGE in gels impregnated with 0.1% peptidoglycan before the gel was washed with ddH_2O and equilibrated in renaturation buffer (10 mM Tris-HCl pH 7.5, 10 μM zinc acetate, 0.1% Triton X-100). Renaturation of the proteins occurred overnight at 37°C and after washing the peptidoglycan was stained with methylene blue (0.1% methylene blue, 0.01% potassium hydroxide) to visualize zones of clearing corresponding to peptidoglycan hydrolase activity.

Peptidoglycan hydrolase assay

Peptidoglycan was isolated from *E. coli* MC1061 (Casadaban and Cohen, 1980) and CS703/1 (Meberg et al., 2001) strains as described previously (Glauner, 1988). Briefly, *E. coli* cultures were grown to mid-exponential phase before centrifugation to pellet the cells. Cell membranes were solubilised by adding the cells in ice-cold water drop-wise into a boiling 8% (w/v) SDS solution under agitation. After cooling overnight, cell sacculi were collected by centrifugation at 130 000 *g* for 1 h at room temperature and washed four times to remove residual SDS. Glycogen and covalently-linked proteins were released by treatment with amylase and pronase, respectively, before boiling in SDS once more. Purified peptidoglycan was washed four more times in distilled water before resuspension in 20 mM sodium phosphate buffer pH 4.8. Reactions were carried out in 50 mM Tris-HCl pH 7.5 containing 150 mM NaCl, 0.05% Triton X-100 and 1 mM $ZnCl_2$. Purified $VgrG2b_{C\text{-}ter}$ or $VgrG2b_{C\text{-}ter}$ (E936A) (5 μM) was incubated with 0.1 mg/ml purified peptidoglycan in a final volume of 100 μl for 4 h at 37°C in a thermoshaker at 850 rpm. Reactions were terminated by boiling the samples at 100°C for 10 min. Next, 20 μl of 20 mM sodium phosphate pH 4.8 and 1 μM cellosyl muramidase (kindly provided by Hoechst, Germany) were added and incubated over night at 37°C. Samples were centrifuged at 13 000 rpm for 10 min and the supernatant containing the soluble muropeptides was collected. Samples were reduced using sodium borohydride and adjusted to pH 4-5 before reduced muropeptides were separated by reversed-phase HPLC (Glauner, 1988).

Microscopy techniques

E. coli DH5α harboring pTat plasmids were grown to OD_{600} 0.5, resuspended in LB containing chloramphenicol, 0.2% arabinose and 5 μg/ml FM1-43 lipophilic dye, and 1 μl was spotted onto a coverslip under an agarose pad for microscopic analysis. Growth of bacteria was monitored every 30 min in a heated chamber at 37°C using an Axio Observer Z1 epifluorescent microscope (Zeiss). Image processing and cell length measurements were performed with FIJI software. Analysis of *P. aeruginosa* cell morphology was conducted similarly; however, competitions were initialised as described in the section "Bacterial competition assays." To investigate the role of DsbA in PA0261-mediated neutralisation of periplasmic $VgrG2b_{C\text{-}ter}$, *E. coli* MC1000 or its *dsbA* mutant derivative harboring the relevant plasmids were normalized to OD_{600} 1 after overnight growth. Serial dilution followed and spots were plated on LB agar containing the appropriate antibiotics in inducing or repressing conditions. Images of the spots were acquired with a Leica M205FA stereomicroscope with a 1x objective and colony area measurements were also determined within FIJI software.

Mass spectrometry analysis

Mass spectrometry analysis was conducted by the Plateforme Protéomique Structurale et Fonctionelle at the Institut Jacques Monod, Paris. Briefly, samples underwent on-bead digestion with 12.5 μg/ml sequencing-grade trypsin (Promega), before peptide analysis on an Orbitrap Fusion Tribrid mass spectrometer coupled to an Easy-spray nanoelectrospray ion source and an Easy nano-LC Proxeon 1000 liquid chromatography system (all Thermo Scientific). Chromatographic separation of the peptides was achieved by an Acclaim PepMap 100 C18 pre-column and a PepMap-RSLC Proxeon C18 column at a flow rate of 300 nl/min. The solvent gradient consisted of 95% solvent A (water, 0.1% (v/v) formic acid) to 35% solvent B (100% acetonitrile, 0.1% (v/v) formic acid) over 98 minutes. An Orbitrap mass spectrometer analyzed the peptides in full ion scan mode, with the resolution set at 120 000 with a *m/z* mass range of 350 - 1550. High energy collision-induced dissociation activation with a collisional energy of 28% permitted fragment acquisition with the quadruple isolation width of 1.6 Da. The linear ion trap was employed in top-speed mode to acquire the MS/MS data with a 50 s dynamic exclusion and a 1 min repeat duration. Maximum ion accumulation times were set to 250 ms for MS acquisition and 60 ms for MS/MS acquisition in parallelisation mode. A MASCOT search server (Matrix Science, version 2.5.1) was used in-house to identify the peptides. Here, a mass tolerance of 7 ppm was set for precursor ions and 0.5 Da for fragments. Identified modifications included acetylation (N-terminal), oxidation (Met) and phosphorylation (Ser, Thr or Tyr), and two missed trypsin cleavage sites were permitted. The *E. coli* SwissProt database (August 2017) was searched using the MS/MS data, while searching the *P. aeruginosa* database confirmed the presence of the bait proteins. A false-discovery rate of 1% was determined with the percolator algorithm, below which the identities of peptides could be assigned.

Analysis of lipoprotein abundance

Overnight cultures of *E. coli* BL21 (λDE3) harboring pET28a-*mltC*, -*lolB*, *rcsF* or pET22b were back-diluted in LB containing the appropriate antibiotics and construct expression was induced with 100 μM IPTG at OD_{600} 0.4 for 3 h. Cells were harvested by centrifugation at 8 000 *g* for 3 min and resuspended in lysis buffer (100 mM Tris-HCl pH 7.5, 50 mM NaCl, 10 μM zinc acetate) for cell disruption by sonication. Lysates were clarified of cellular debris by sonication three times at 4 000 *g* for 15 min at 4°C before incubation with 10 μg $VgrG2b_{C\text{-}ter}$ or $VgrG2b_{C\text{-}ter}$ (E936A) at 37°C for 1 h. Ultracentrifugation at 100 000 *g* for 1 h at 4°C separated the soluble and

membrane fractions, to which Laemmli buffer was added for subsequent SDS-PAGE and immunoblot analysis. Production of MltC, LolB and RcsF was monitored using antibodies against the C-terminal hexahistidine tag, while production of TEM-1 from pET22b was detected using a monoclonal antibody. Densitometry was conducted in FIJI software.

Bioinformatic and phylogenetic analysis

Protein sequences were obtained from the non-redundant NCBI database using the BLASTp algorithm, alignments were done in Jalview using MAFTT and sequence logos were generated using the Weblogo server (Crooks et al., 2004; Katoh et al., 2017). The prediction of bacterial lipoprotein signal peptides, type I signal peptides and transmembrane helices was undertaken using LipoP 1.0, SignalP 4.1 and TMHMM server v2.0, respectively (Krogh et al., 2001; Nielsen, 2017). The search algorithm Jackhmmer (EMBL-EBI) was used to search for homologs of the VgrG2b metallopeptidase domain. Three iterations querying the UniProtKB database were conducted, retrieving 240 sequences. Sequences were aligned and MEGA7 was employed for phylogenetic analysis, using the maximum-likelihood method of tree generation with 1000 bootstrap replicates (Kumar et al., 2016).

QUANTIFICATION AND STATISTICAL ANALYSIS

The statistical analyses described in this work were conducted using Prism 8.0 software (GraphPad) and the statistical parameters, including sample sizes (where *n* indicates the number of independent biological replicates, technical replicates or bacterial cells), *p* values and statistical tests performed are indicated in the relevant figure legends. The means of biologically independent replicates were compared using the following statistical tests: Student's t test, a one-way ANOVA followed by Dunnett's or Tukey's multiple comparisons tests. The spread is reported with the standard error of the mean (SEM), or by the median and quartiles for pooled single cell analyses.

DATA AND CODE AVAILABILITY

The accession numbers for the crystal structure and diffraction datasets of VgrG2b$_{C\text{-}ter}$ reported in this paper are PDB: 6H56 and Zenodo: 10.5281/zenodo.3246346, respectively.

Cell Reports, Volume *29*

Supplemental Information

The *Pseudomonas aeruginosa* T6SS Delivers a Periplasmic Toxin that Disrupts Bacterial Cell Morphology

Thomas E. Wood, Sophie A. Howard, Andreas Förster, Laura M. Nolan, Eleni Manoli, Nathan P. Bullen, Hamish C.L. Yau, Abderrahman Hachani, Richard D. Hayward, John C. Whitney, Waldemar Vollmer, Paul S. Freemont, and Alain Filloux

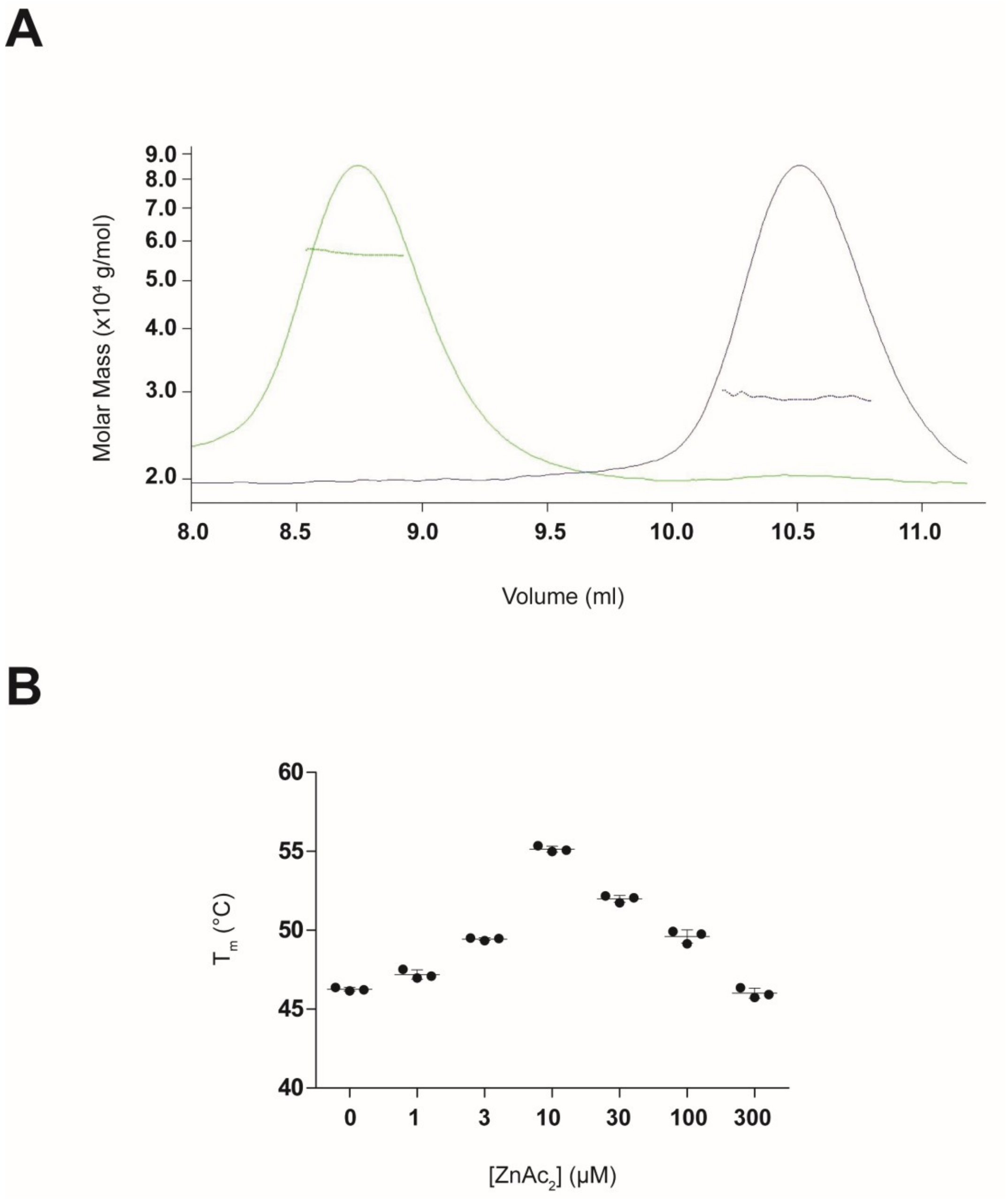

Figure S1: The VgrG2b metallopeptidase exists as monomeric and dimeric species in solution and is stabilised by zinc. Related to Figure 2.

A SEC-MALLS chromatogram of VgrG2b$_{C\text{-ter}}$ purified from *E. coli*. Solid lines show UV traces of fractions pooled from two peaks obtained after preparatory size-exclusion chromatography. Dashed lines across the peaks show the MALLS-derived apparent masses. The calculated weight-average molecular masses are 56.7 kD (left peak) and 29.1 kD (right peak) corresponding to the dimeric and monomeric species, respectively.

B Thermostability of the purified VgrG2b$_{C\text{-ter}}$ domain in the presence of different concentrations of zinc ions as determined by calculation of the melting temperature using differential scanning fluorimetry. Individual points represent the mean of three technical replicates with error bars showing the SEM (*n* = 3 technical replicates).

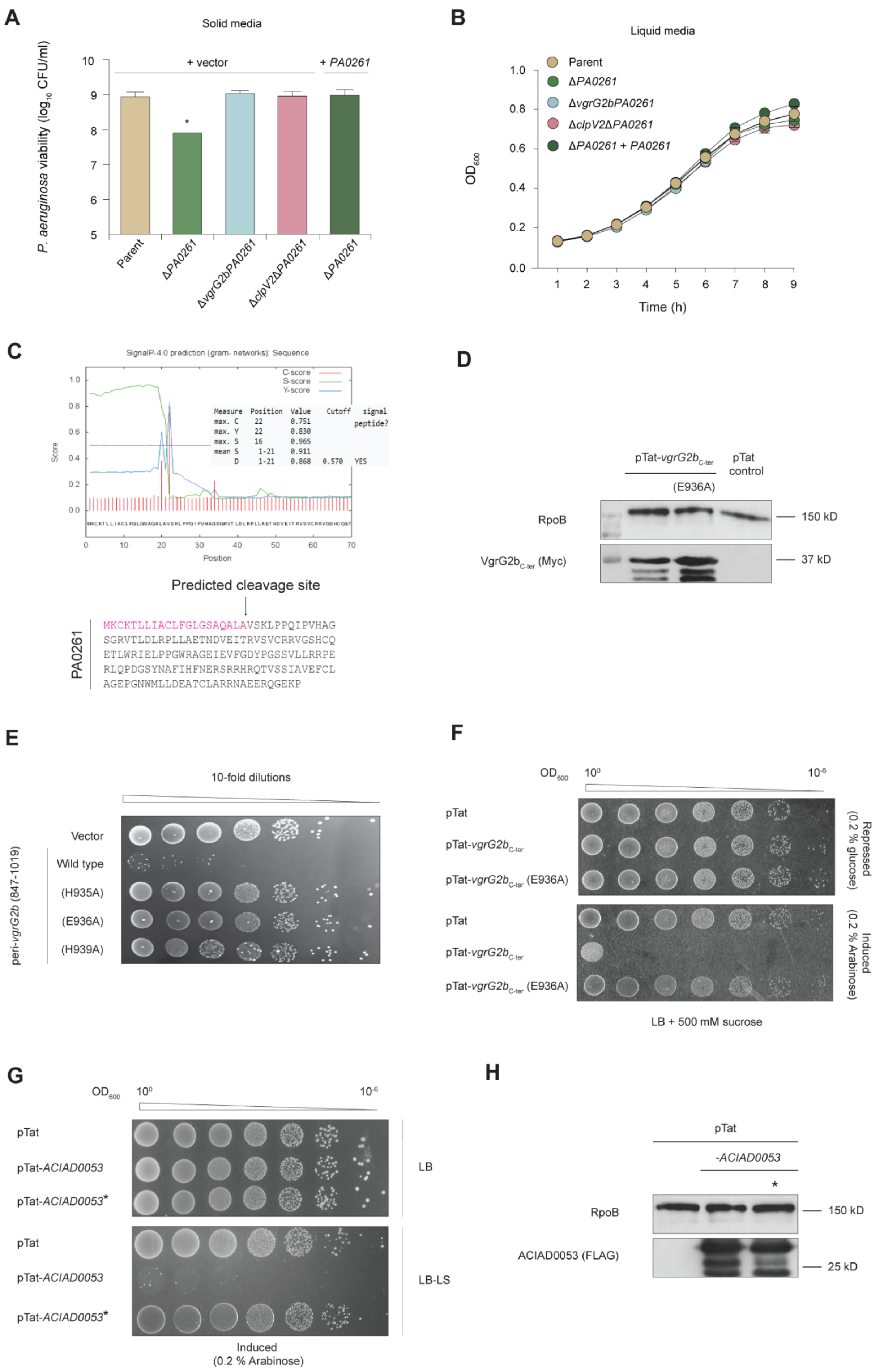

A
Solid media
P. aeruginosa viability (log10 CFU/ml)
+ vector
+ PA0261
Parent
ΔPA0261
ΔvgrG2bPA0261
ΔclpV2ΔPA0261
ΔPA0261
B
Liquid media
Parent
ΔPA0261
ΔvgrG2bPA0261
ΔclpV2ΔPA0261
ΔPA0261 + PA0261
OD600
Time (h)
C
SignalP-4.0 prediction (gram- networks): Sequence
C-score
S-score
Y-score
Measure Position Value Cutoff signal peptide?
max. C 22 0.751
max. Y 22 0.830
max. S 16 0.965
mean S 1-21 0.911
D 1-21 0.868 0.570 YES
Score
Position
Predicted cleavage site
PA0261
MKCKTLLIACLFGLGSAQALAVSKLPPQIPVHAG
SGRVTLDLRPLLAETNDVEITRVSVCRRVGSHCQ
ETLWRIELPPGWRAGEIEVFGDYPGSSVLLRRPE
RLQPDGSYNAFIHFNERSRRHRQTVSSIAVEFCL
AGEPGNWMLLDEATCLARRNAEERQGEKP
D
pTat-vgrG2bC-ter
(E936A)
pTat control
RpoB
VgrG2bC-ter (Myc)
150 kD
37 kD
E
10-fold dilutions
peri-vgrG2b (847-1019)
Vector
Wild type
(H935A)
(E936A)
(H939A)
F
OD600 10^0 10^-6
pTat
pTat-vgrG2bC-ter
pTat-vgrG2bC-ter (E936A)
Repressed (0.2 % glucose)
Induced (0.2 % Arabinose)
LB + 500 mM sucrose
G
OD600 10^0 10^-6
pTat
pTat-ACIAD0053
pTat-ACIAD0053*
LB
LB-LS
Induced (0.2 % Arabinose)
H
pTat
-ACIAD0053
*
RpoB
ACIAD0053 (FLAG)
150 kD
25 kD

Figure S2: Characterisation of the VgrG2b-PA0261 effector-immunity pair family. Related to Figure 4.
A Self-intoxication assay of *P. aeruginosa* on solid media. Viability of the indicated strains assessed by enumeration of colony forming units (CFU). Here, the parental strain is *P. aeruginosa* PA14Δ*rsmA*Δ*amrZ*. A statistically significant difference in recovered prey *versus* the parental strain was determined using a one-way ANOVA followed by Dunnett's multiple comparisons test (n = 3 biological replicates; * $p < 0.05$).
B Self-intoxication assay of *P. aeruginosa* in liquid culture. Viability was measured by monitoring turbidity (OD_{600}) in a 96-well format. The parental strain is the same as in panel **A**. Mean values are plotted from three biologically independent experiments, with error bars representing the standard deviation (n = 3).
C The putative N-terminal signal peptide of PA0261. The upper panel shows the prediction output of the SignalP 4.1 server. The lower panel indicates the predicted signal peptide (in pink) and putative signal peptidase cleavage site within the sequence of the PA0261 polypeptide.
D Immunoblot showing that both active and inactive VgrG2b$_{\text{C-ter}}$ variants are produced in *E. coli* from the pTat plasmids. Both isoforms are detected with anti-myc antibodies to a C-terminal tag. RpoB is a loading control.
E *E. coli* periplasmic toxicity assay showing the requirement of the metallopeptidase catalytic triad. VgrG2b metallopeptidase constructs (residues 847-1019) were targeted to the periplasm by an N-terminal PelB signal peptide. Strains were serially diluted on solid media under inducing conditions. Image is representative of three independent experiments.
F Plating efficiency of *E. coli* harbouring pTat, pTat-*vgrG2b*$_{\text{C-ter}}$ or pTat-*vgrG2b*$_{\text{C-ter}}$ (E936A) in hypertonic conditions (LB agar with 500 mM sucrose). Expression from the plasmid is repressed with 0.2% glucose or induced with 0.2% arabinose.
G Targeting the *A. baylyi* VgrG2b$_{\text{C-ter}}$ metallopeptidase homologue ACIAD0053 to the *E. coli* periplasm using the TorA Tat-dependent signal peptide of the pTat vector. ACIAD0053* is the catalytic mutant of the effector where the HEMGH motif (residues 102-106) has been mutated to AAMGA. Plating efficiency of ten-fold serially diluted *E. coli* cultures on LB agar or low-salt LB agar (LB-LS) containing 0.2% arabinose inducer are shown. Image is representative of three independent experiments.
H Immunoblot detecting the FLAG-tagged ACIAD0053 toxin and its inactive AAMGA variant (designated by the asterisk). RpoB is a loading control.

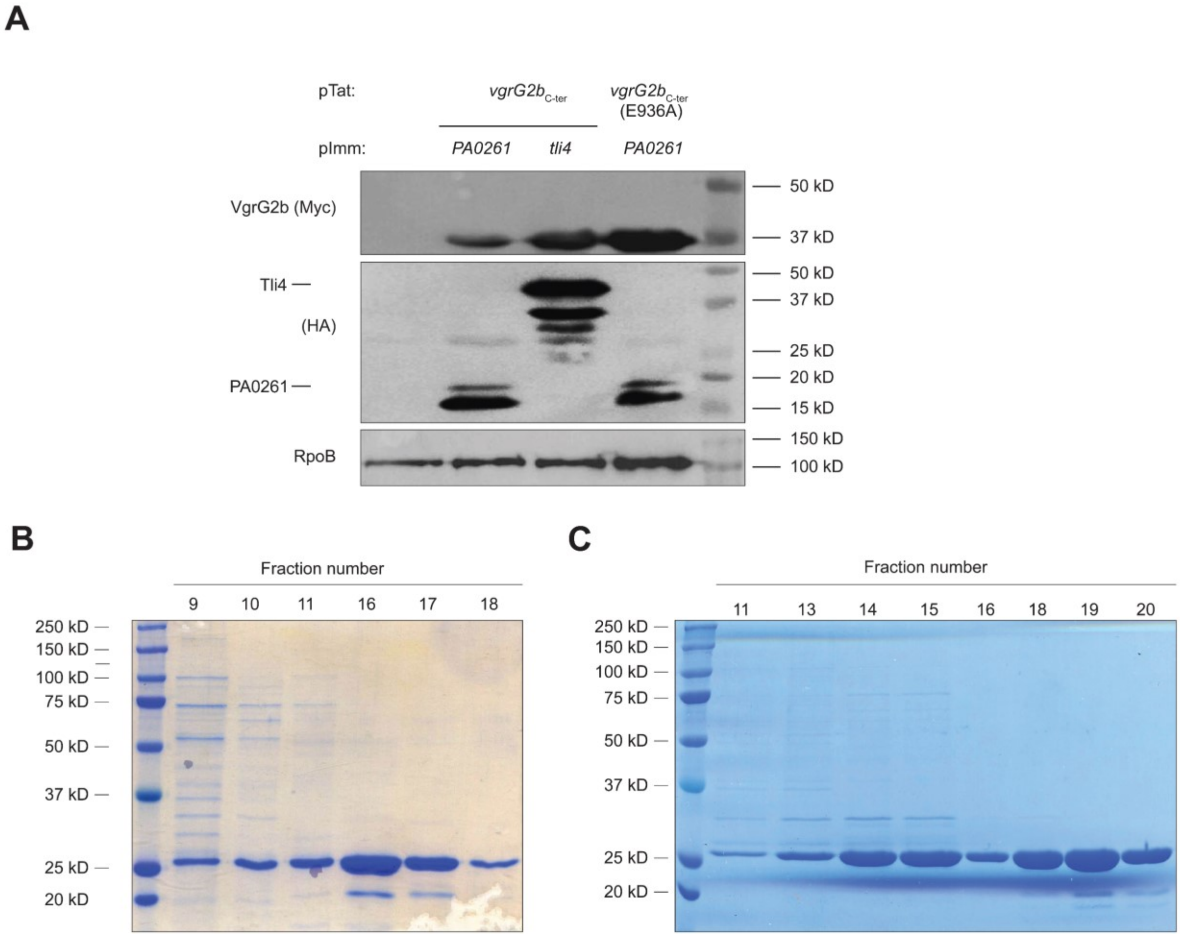

Figure S3: Production of immunity proteins and purification of VgrG2b$_{\text{C-ter}}$. Related to Figure 5.
A Immunoblot showing the production of the Myc-tagged VgrG2b$_{\text{C-ter}}$ effector and HA-tagged immunity proteins expressed in *E. coli*. RpoB acts as a loading control.
B and **C** SDS-PAGE analysis of recombinant VgrG2b$_{\text{C-ter}}$ (**B**) and VgrG2b$_{\text{C-ter}}$ (E936A) (**C**) elution after size-exclusion chromatography following Ni^{2+}-NTA affinity chromatography. Proteins were visualised by Coomassie staining. Fraction numbers are shown for each lane.

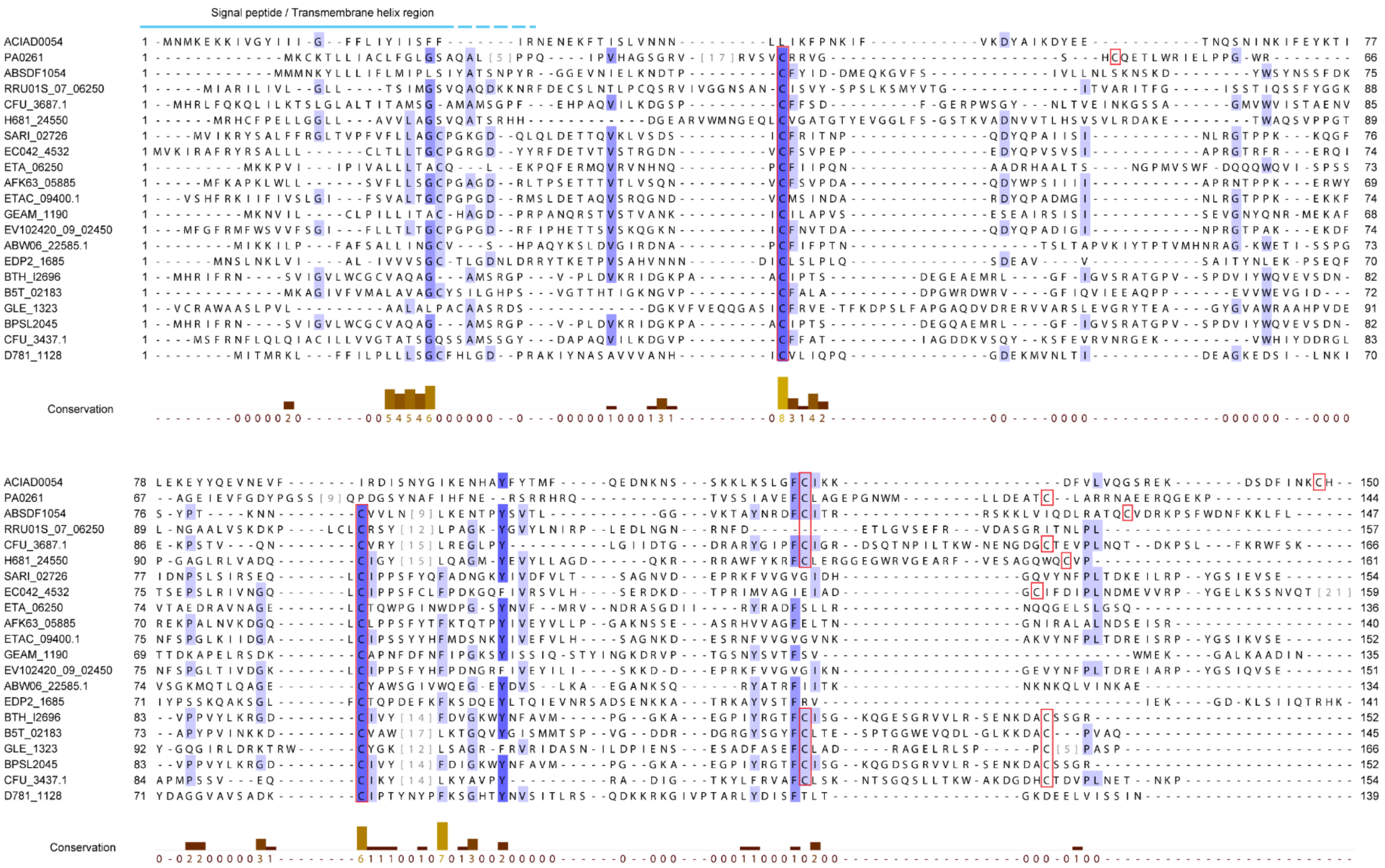

Figure S4: Alignment of immunity proteins in the PA0261 family. Related to Figure 5.

Multiple sequence alignment of putative immunity proteins for metallopeptidase effectors of the VgrG2b$_{C\text{-}ter}$ family. Due to low sequence homology, the alignment was manually curated after initial alignment by MAFFT. Omitted residues are shown in grey and cysteine residues are highlighted with red boxes. Darker coloration of the residues reflects the increasing conservation of identity, with the scores for each position shown below the alignment.

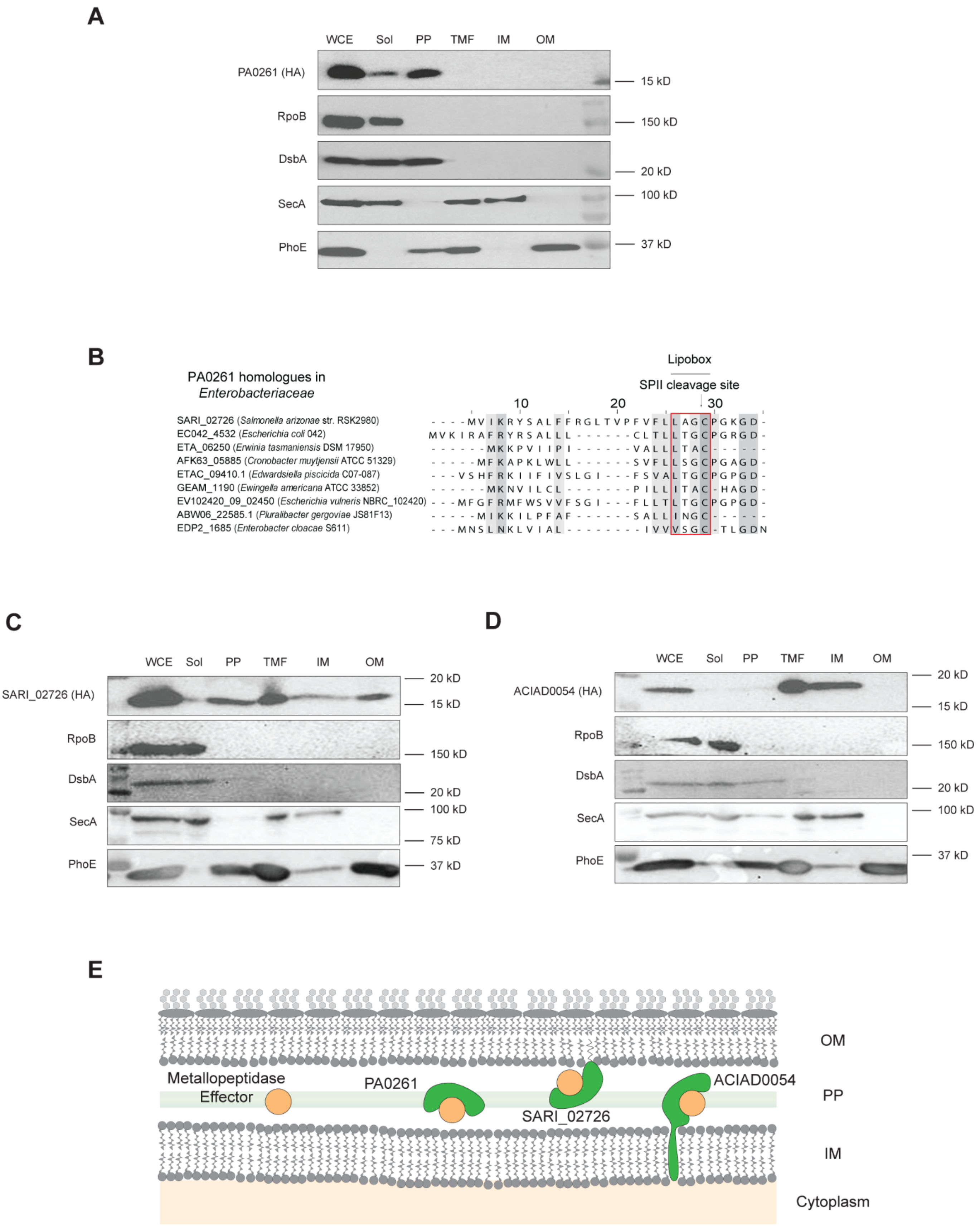

A
WCE Sol PP TMF IM OM
PA0261 (HA)
RpoB
DsbA
SecA
PhoE
15 kD
150 kD
20 kD
100 kD
37 kD
B
PA0261 homologues in *Enterobacteriaceae*
Lipobox
SPII cleavage site
10 20 30
SARI_02726 (*Salmonella arizonae* str. RSK2980) ----MVIKRYSALFFRGLTVPFVFLLAGCPGKGD-
EC042_4532 (*Escherichia coli* 042) MVKIRAFRYRSALLL-------CLTLLTGCPGRGD-
ETA_06250 (*Erwinia tasmaniensis* DSM 17950) ------MKKPVIIPI-------VALLLTAC------
AFK63_05885 (*Cronobacter muytjensii* ATCC 51329) -----MFKAPKLWLL-------SVFLLSGCPGAGD-
ETAC_09410.1 (*Edwardsiella piscicida* C07-087) ---VSHFRKIIFIVSLGI---FSVALTGCPGPGD-
GEAM_1190 (*Ewingella americana* ATCC 33852) ------MKNVILCL--------PILLITAC-HAGD-
EV102420_09_02450 (*Escherichia vulneris* NBRC_102420) ---MFGFRMFWSVVFSGI---FLLTLTGCPGPGD-
ABW06_22585.1 (*Pluralibacter gergoviae* JS81F13) -----MIKKILPFAF-------SALLINGC------
EDP2_1685 (*Enterobacter cloacae* S611) ---MNSLNKLVIAL---------IVVVSGC-TLGDN
C
WCE Sol PP TMF IM OM
SARI_02726 (HA)
RpoB
DsbA
SecA
PhoE
20 kD
15 kD
150 kD
20 kD
100 kD
75 kD
37 kD
D
WCE Sol PP TMF IM OM
ACIAD0054 (HA)
RpoB
DsbA
SecA
PhoE
20 kD
15 kD
150 kD
20 kD
100 kD
37 kD
E
OM
Metallopeptidase Effector
PA0261
SARI_02726
ACIAD0054
PP
IM
Cytoplasm

Figure S5: Members of the PA0261 immunity protein family utilise diverse mechanisms to access the periplasmic compartment. Related to Figure 5.

A Immunoblot analysis of the subcellular localisation of HA-tagged PA0261. RpoB is used as a marker of the soluble fraction (Sol), DsbA for the periplasm (PP), SecA for the inner membrane (IM) and PhoE for the outer membrane (OM). WCE: whole cell extract; TMF: total membrane fraction. Immunoblots are representative of three independent experiments.

B Multiple sequence alignment of the N-terminal region of PA0261 orthologues within *Enterobacteriaceae*. The red box demarcates a putative lipobox sequence and the predicted signal peptidase II (SPII) cleavage site is marked. Light to dark grey shading indicates an increasing level of residue conservation.

C and **D** Immunoblot of fractionated *E. coli* cells producing HA-tagged PA0261 orthologues from *S. arizonae* (SARI_02726; **C**) and *A. baylyi* (ACIAD0054; **D**). See panel **A** for details of fraction markers.

E Model showing the subcellular localisation of the three metallopeptidase immunity proteins PA0261, SARI_02726 and ACIAD0054. These immunity determinants, depicted in green, are a soluble periplasmic protein, an outer membrane lipoprotein and an inner membrane protein, respectively. The periplasmic VgrG2b$_{C\text{-}ter}$-family metallopeptidase is represented as an orange circle. Immunoblots are representative of three independent experiments.

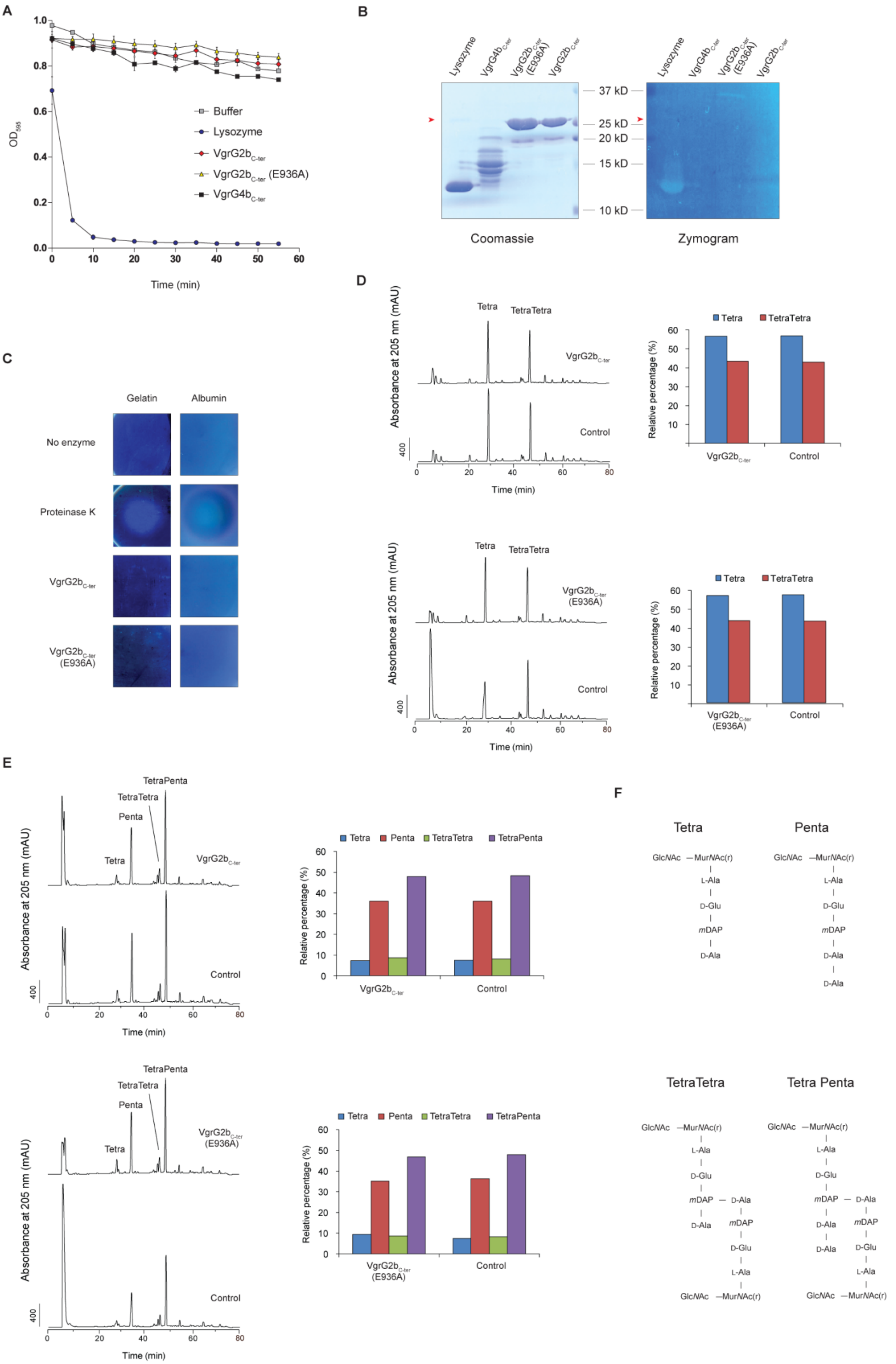

A
OD595
Time (min)
Buffer
Lysozyme
VgrG2bC-ter
VgrG2bC-ter (E936A)
VgrG4bC-ter
B
Lysozyme
VgrG4bC-ter
VgrG2bC-ter (E936A)
VgrG2bC-ter
37 kD
25 kD
20 kD
15 kD
10 kD
Coomassie
Zymogram
C
Gelatin
Albumin
No enzyme
Proteinase K
VgrG2bC-ter
VgrG2bC-ter (E936A)
D
Absorbance at 205 nm (mAU)
Tetra
TetraTetra
VgrG2bC-ter
Control
VgrG2bC-ter (E936A)
Time (min)
Relative percentage (%)
E
TetraPenta
Penta
F
Tetra
Penta
TetraTetra
Tetra Penta
GlcNAc
MurNAc(r)
L-Ala
D-Glu
mDAP
D-Ala

Figure S6: VgrG2b$_{C\text{-}ter}$ displays no detectable peptidoglycan hydrolase or generic protease activity. Related to Figure 6.

A Recombinant VgrG2b$_{C\text{-}ter}$ does not cause lysis of *M. lysodeikticus* as measured by turbidometry. Incubation with lysozyme acts as the positive control, while the buffer-only condition and addition of recombinant VgrG4b$_{C\text{-}ter}$ act as the negative controls. Points and error bars represent the mean ± SEM (n = 3 technical replicates).

B Recombinant VgrG2b$_{C\text{-}ter}$, VgrG2b$_{C\text{-}ter}$ (E936A), VgrG4b$_{C\text{-}ter}$ and lysozyme were assessed for peptidoglycan hydrolytic activity by zymography with a purified *E. coli* peptidoglycan substrate. Samples were run on a 12% SDS-PAGE gel containing 0.1% peptidoglycan and stained with a methylene blue solution to visualise zones of clearing, indicative of peptidoglycan hydrolysis. A Coomassie-stained gel acts as a loading control for the purified proteins. The arrows highlight the location of the band corresponding to VgrG2b$_{C\text{-}ter}$.

C 10 µg VgrG2b$_{C\text{-}ter}$, VgrG2b$_{C\text{-}ter}$ (E936A) or proteinase K was spotted onto bacteriological agar plates containing 1% bovine serum albumin or gelatin. After incubation at 37 °C for 24 h, amido black staining was used to visualise zones of clearing indicative of proteolytic activity.

D and **E** HPLC chromatograms showing the profiles of the major muropeptides (*left panels*) released from tetrapeptide-rich *E. coli* MC1061 (**D**) and pentapeptide-rich CS703/1 (**E**) sacculi after incubation with VgrG2b$_{C\text{-}ter}$, VgrG2b$_{C\text{-}ter}$ (E936A) or a buffer-only control. The composition of muropeptide species in control and assay reactions is also shown (*right panels*).

F Structures of the muropeptides shown in panels **D** and **E**. Abbreviations: Glc*N*Ac, *N*-acetylglucosamine; Mur*N*Ac(r), *N*-acetylmuramitol; L-Ala, L-alanine; D-Glu, D-glutamic acid; *m*DAP, *meso*-diaminopimelic acid; D-Ala, D-alanine.

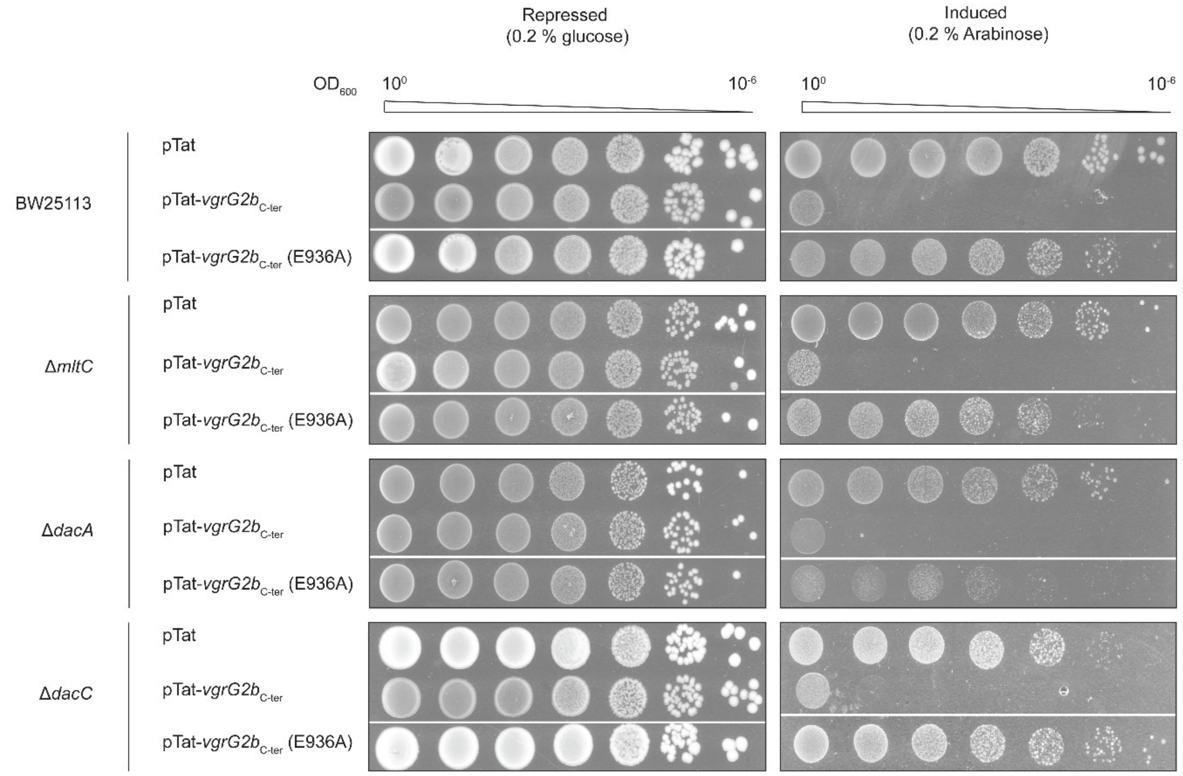

Figure S7: MltC, PBP5 and PBP6a individually dispensable for VgrG2b$_{\text{C-ter}}$-mediated toxicity. Related to Figure 7.

Plating efficiency of *E. coli* strains harbouring pTat vector, pTat-*vgrG2b*$_{\text{C-ter}}$ or pTat-*vgrG2b*$_{\text{C-ter}}$ (E936A). Strains are wild type BW25113, *mltC*, *dacA* (encoding PBP5) and *dacC* (encoding PBP6a) mutants. Serial dilutions assessed toxicity in repressive (0.2% glucose) and inducing (0.2% arabinose) conditions. Images are representative of three biological replicates.

Table S1

	VgrG2b (833-1013)
Data processing	
Wavelength (Å)	0.9795
Resolution Range (Å)	43.9 - 3.2 (3.42 - 3.2)[a]
Space Group	$P3_1$
Unit Cell	
a, *b*, *c* (Å)	78.3, 78.3, 115.3
α, *β*, *γ* (°)	90, 90, 120
Total Reflections	45339
Unique Reflections	12782
Multiplicity	3.5 (3.6)[a]
Completeness (%)	99.6 (99.9)[a]
$I/\sigma I$	12.5 (2.0)[a]
d_{min} for I/sig(I) > 1.0 (Å)	3.1
Wilson B-factor	123.5
R_{sym} (%)	5.7 (46.2)[a]
Model refinement	
R value	0.223
R_{free}	0.232
Number of Atoms	2745
Protein Residues	360
Water Molecules	0
Root-mean-square Deviations	
Bond lengths (Å)	0.009
Bond angles (°)	1.01
Ramachandran Favoured (%)	95.5%
Ramachandran Outliers (%)	0
Clash Score	4

a. Values within parentheses denote the resolution of the highest resolution shell

Table S1: Data collection and refinement statistics for the crystal structure of the VgrG2b metallopeptidase domain. Related to Figure 2.

Table S2

	Pos 1	Pos 4	Pos 5	Pos 6	All data
**Data collection**					
Wavelength (Å)	0.97949				
Oscillation width (°)	0.15				
Number of images	2400				9600
**Data processing**					
Space group	P 4 2 2				
Unit cell parameters	168.8, 79.37	169.4, 79.37	169.4, 79.20	168.7, 79.15	168.8, 79.37
Resolution range (Å)	40-2.7 (2.86-2.7)[a]	40-2.8 (2.97-2.8)[a]	40-2.9 (3.07-2.9)[a]	40-2.8 (2.97-2.8)[a]	40-2.7 (2.85-2.7)[a]
Unique reflections	60251 (9706)[a]	54432 (8760)[a]	48913 (7849)[a]	53864 (8712)[a]	60184 (8967)[a]
Multiplicity	14.0 (13.5)[a]	13.6 (12.1)[a]	13.7 (13.6)[a]	13.5 (12.2)[a]	49.3 (20.6)[a]
R_{meas} (lowest resolution)	0.073	0.061	0.071	0.079	0.095
ISa	14.7	16.7	21.8	14.2	N/A
R_{meas}	0.323 (6.17)[a]	0.304 (9.26)[a]	0.413 (8.15)[a]	0.345 (6.53)[a]	0.381 (7.60)[a]
Completeness	0.999 (0.998)[a]	0.999 (0.999)[a]	0.999 (0.997)[a]	1.000 (1.000)[a]	0.999 (0.999)[a]
$\langle I/\sigma(I)\rangle$	6.66 (0.13)[a]	7.80 (0.16)[a]	7.38 (0.22)[a]	6.65 (0.23)[a]	9.91 (0.13)[a]
$CC_{1/2}$	0.997 (0.277)[a]	0.997 (0.124)[a]	0.996 (0.201)[a]	0.997 (0.256)[a]	0.999 (0.251)[a]
d_{min} for I/sig(I) > 1.0 (Å)	3.0	3.1	3.2	3.1	3.0
d_{min} for SigAno[b] > 1.0 (Å)	4.65	4.82	3.88	4.19	4.27

a. Values within parentheses denote the resolution of the highest resolution shell
b. SigAno = $|F^{+}-F^{-}|/\sigma(F)$

Table S2: Data collection and processing statistics for crystals of selenomethionine-derivatised VgrG2b metallopeptidase domain. Related to Figure 2.

Table S3

UniProt Accession	Protein[a]	Replicate 1				Replicate 2					Replicate 3				
		Coverage (%)	Unique Peptides	MASCOT Score (a)	MASCOT Score (b)	Coverage (%)	Unique Peptides	MASCOT Score (a)	MASCOT Score (b)	MASCOT Score (c)	Coverage (%)	Unique Peptides	MASCOT Score (a)	MASCOT Score (b)	MASCOT Score (c)
Cell envelope															
Q9I6M7	VgrG2b$_{C\text{-ter}}$ (E936A)	88	26		14949	95	29	2014	24367	4989	92	26	298	8024	192
P0C066	MltC[d]	62	12		489						35	6		103	
P76537	YfeY[d]	37	4		155	36	4		159		29	4	0	379	26
P08506	PBP6a					58	14	46	455		44	15	30	172	
P0AEB4	PBP5					58	18	215	417		29	9	23	156	23
P18392	RstB					13	4		237		17	5		203	
P0A938	BamE[d]	76	3		199						76	3	0	166	
P69778	Lpp[d]	33	2	28	131	33	2	53	129		33	2	16	283	20
A7ZKY3	LolB[d]	54	9		227						46	8		113	
P0AC04	BamD[d]					33	6	119	250		39	7		152	
P77774	BamB[d]					47	10	77	189		35	9	14	157	
P0A903	BamC[d]	22	5	14	153						46	9	45	129	
P0AAS1	YlaC					33	4	31	195		33	5		134	
Cytoplasmic															
P0ACZ8	CusR	52	11	326	824	45	7	72	251	211					
P13035	GlpD					69	28	56	1063		33	13	44	169	28
P26616	MaeA	32	9	20	287	52	19	287	695						
P67095	YfcE					36	4	39	154	35	28	3	19	100	25
P0AGK8	IscR	38	3	35	108	54	4	34	220	0					
P0AEZ1	MetF	23	5	58	117	20	4	40	133	61					
P00561	ThrA					30	13	49	224		13	8	34	136	40
Cell envelope (below threshold)															
P69412	RcsF[d]	26	3		158						10	1		33	

a: BL21 pET22b-*vgrG2b*$_{C\text{-ter}}$ (E936A)

b: BL21 pET22b-*vgrG2b*$_{C\text{-ter}}$ (E936A)-*strepII*

c: BL21 pET22b-*vgrG2a*$_{C\text{-ter}}$ -*strepII*

d: Lipoprotein

Table S3: Interactome of VgrG2b$_{C\text{-}ter}$ (E936A) in the *E. coli* cell envelope. Related to Figure 7.
The MASCOT scores indicate the enrichment of the proteins pulled down within the three independent replicates, presenting the interactome of periplasmic VgrG2b$_{C\text{-}ter}$ (E936A) in *E. coli*. Proteins localised in the cytoplasm were discounted as relevant interaction partners. The UniProt accession number, defining identity of interacting partners, is also shown alongside the coverage and the number of unique peptides found in each replicate. The MASCOT score corresponds to -10log(*P*), where *P* is the calculated probability that the observed match between the experimental data and the database sequence is a random event and serves as a statistical representation of the positive identification of the protein in the sample, with higher scores being more significant. Note that replicates were entirely independent so quantitative comparisons may not be conducted.
